# Effects and Safety of the Tripterygium Glycoside Adjuvant Methotrexate Therapy in Rheumatoid Arthritis: A Systematic Review and Meta-Analysis

**DOI:** 10.1155/2022/1251478

**Published:** 2022-03-24

**Authors:** Qi Geng, Bin Liu, Yanfang Ma, Huizhen Li, Nannan Shi, Guilin Ouyang, Zhixing Nie, Jianfeng Yi, Yaolong Chen, Yanping Wang, Cheng Lu

**Affiliations:** ^1^Institute of Basic Research in Clinical Medicine, China Academy of Chinese Medical Sciences, Beijing 100700, China; ^2^Chemical and Biological Engineering, Yichun University, Yichun 336000, China; ^3^Evidence Based Medicine Center, School of Basic Medical Sciences, Lanzhou University, Lanzhou 730000, China; ^4^WHO Collaborating Centre for Guideline Implementation and Knowledge Translation, Lanzhou University, Lanzhou 730000, China; ^5^Chinese GRADE Center, Lanzhou 730000, China; ^6^Shanghai Guanghua Hospital of Integreted Tranditional Chinese and Western Medicine, Shanghai 200050, China; ^7^Research Center for Differentiation and Development of Traditional Chinese Medicine Basic Theory, Jiangxi Uiniversity of Traditional Chinese Medicine, Nanchang, China

## Abstract

**Objective:**

This study aimed to systematically review the efficacy and clinical safety of different courses and doses of tripterygium glycoside (TG) adjuvant methotrexate (MTX) therapy in the treatment of rheumatoid arthritis (RA).

**Methods:**

Randomized controlled trials (RCTs) of TG adjuvant MTX therapy in patients with RA were retrieved from SinoMed, China Network Knowledge Infrastructure, WanFang Data, PubMed, Cochrane Library, and Embase from inception to September 30, 2021. The effects and clinical safety evaluations were conducted using RevMan 5.3 software.

**Results:**

A total of 9 RCTs and 892 patients with RA were included in this study. In the meta-analysis, a total of 463 and 429 patients were enrolled into the TG adjuvant MTX therapy group and MTX monotherapy group, respectively. In comparison with MTX monotherapy, the results of the analyzed effects showed that the TG adjuvant MTX therapy can achieve 20%, 50%, and 70% improvements in American College of Rheumatology (ACR) criteria ACR20, ACR50, and ACR70 at *P* = 0.005, *P* = 0.0001, and *P* = 0.004, respectively. Simultaneously, the efficacy of the TG adjuvant MTX therapy was improved at either 30 or 60 mg/day over a six-month course compared to MTX monotherapy (*P* < 0.0001). There was no statistical difference in the effects between the doses of 30 and 60 mg/day after three months (*P* = 0.82). TG adjuvant MTX also reduced the expression rate of the swollen joint count, tender joint count, erythrocyte sedimentation rate, rheumatoid factor, and C-reactive protein in subgroup analyses with different courses and doses. In terms of hepatic adverse effects (*P* = 0.28), leukopenia (*P* = 0.78), gastrointestinal adverse effects (*P* *=* 0.17), cutaneous adverse effects (*P* = 0.94), and irregular menstruation adverse effects (*P* = 0.29), there was no statistically significant difference with TG adjuvant MTX therapy and MTX monotherapy with different courses and doses.

**Conclusions:**

TG adjuvant MTX therapy is more effective than MTX monotherapy and is a safe strategy for RA treatment in doses of 30 or 60 mg/day over a treatment course of six months. However, high-quality multicenter RCT studies with large sample sizes are still needed to confirm the effects and clinical safety of different courses and doses of TG adjuvant MTX therapy.

## 1. Introduction

Rheumatoid arthritis (RA) is a complex, inflammatory, and systemic autoimmune disease. It is associated with progressive disability, and it primarily affects the lining of the synovial joints [1, 2]. Patients with RA typically present with symmetrical polyarthritis of the small joints of the hands and feet with early morning stiffness and occasional constitutional symptoms [3]. Methotrexate (MTX) is the first-line treatment given to patients with RA. MTX can reduce the level of inflammation and prevent joint erosion and functional damage. The clinical effect of using MTX monotherapy is only 60% to 70%, whereas adjuvant therapy has positive significance in improving clinical effects [4]. Therefore, the clinical treatment of RA often uses MTX and adjuvant drugs, such as sinomenine and iguratimod, to enhance effects [5, 6].

Traditional Chinese herbal medicine has achieved considerable progress in treating RA [7, 8]. Tripterygium glycosides (TGs), which are the extracts of *Tripterygium wilfordii* Hook F, have been widely used as anti-inflammatory drugs and immunosuppressants for treating RA. The effective parts of TGs mainly include diterpenoids, alkaloids, triterpenoids, and glycosides. Most of these active constituents of TGs are effective in anti-inflammation and immunosuppression [9]. In the United States, several clinical trials have shown that TG has good effects in patients with RA because of its anti-inflammatory and immunosuppressive activities [10–12].

During RA treatment, adjuvant therapeutics, which involve several drugs that interact with multiple targets in the molecular networks of RA, may achieve better effects compared with monotherapy [13, 14]. TG has been empirically applied in adjuvant therapy with MTX for RA treatment [15, 16]. Previous studies have shown that TG adjuvant MTX therapy is a more effective strategy than MTX monotherapy for RA treatment and adverse reactions were not aggravated [17–19]. However, the effects and safety of different courses and doses of TG adjuvant MTX therapy in RA need to be further explored.

## 2. Methods

This study was investigated according to the preferred reporting items for systematic review and meta-analysis (PRISMA) 2020 checklist (Supplementary Information 1).

### 2.1. Protocol Registration

This meta-analysis study and its protocol were registered in PROSPERO of the Centre for Reviews and Dissemination (NO. CRD42021224095).

### 2.2. Eligibility Criteria

The eligibility criteria for the enrollment of studies into meta-analysis are as follows: (1) randomized controlled trials (RCTs) comparing TG adjuvant MTX therapy and MTX monotherapy, (2) patients with a diagnosis of RA based on the criteria revised by the American College of Rheumatology (ACR) in 1987 or the ACR/European Association of Anti-Rheumatology Annual in 2010 [20, 21], (3) primary outcomes with 20% improvement in ACR criteria (ACR20), 50% improvement in ACR criteria (ACR50), and 70% improvement in ACR criteria (ACR70), (4) secondary outcomes of swollen joint count (SJC), tender joint count (TJC), erythrocyte sedimentation rate (ESR), rheumatoid factor (RF), C-reactive protein (CRP), hepatic adverse effects, leukopenia, gastrointestinal adverse effects, cutaneous adverse effects, irregular menstruation adverse effects before and after treatment, and (5) studies published in English or Chinese.

The exclusion criteria are as follows: (1) studies that focused only secondary outcomes or safety outcomes without primary outcomes, (2) patients in studies with additional treatment factors in TG adjuvant MTX therapy group and/or the MTX monotherapy group, and (3) incomplete or duplicate data.

### 2.3. Search Strategy

An electronic search of databases, including SinoMed, China Network Knowledge Infrastructure (CNKI), WanFang Data, PubMed, Cochrane Library and Embase, was performed and completed on September 30, 2021. The references of all retrieved articles were also reviewed for potentially relevant studies. The search strategy involved the use of the following keywords: “Tripterygium^*∗*^,” “Tripterygium hypoglaucum,” “Tripterygium hypoglaucums,” “Tripterygiums,” “hypoglaucums, Tripterygium,” “Tripterygium wilfordii,” “Tripterygium wilfordius,” “wilfordius, Tripterygium,” “Leigong Teng,” “Leigong Tengs,” “Teng, Leigong,” “Tengs, Leigong,” “Thundergod Vine,” “Thundergod Vines,” “Vine, Thundergod,” “Vines, Thundergod,” “Arthritis, Rheumatoid^*∗*^,” “Caplan Syndrome^*∗*^,” “Felty Syndrome^*∗*^,” “Rheumatoid Nodule^*∗*^,” “Rheumatoid Vasculitis^*∗*^,” “Sjogren's Syndrome^*∗*^,” “Still's Disease, Adult-Onset^*∗*^,” “Rheumatoid Arthritis,” “Caplan Syndromes,” “Caplan's Syndrome,” “Caplans Syndrome,” “Syndrome, Felty,” “Felty's Syndrome,” “Feltys Syndrome,” “Syndrome, Felty's,” “Familial Felty's Syndrome,” “Familial Feltys Syndrome,” “Felty's Syndrome, Familial,” “Syndrome, Familial Felty's,” “Rheumatoid Arthritis,” “Splenomegaly and Neutropenia,” “Familial Felty Syndrome,” “Felty Syndrome, Familial,” “Syndrome, Familial Felty,” “Nodule, Rheumatoid,” “Nodules, Rheumatoid,” “Rheumatoid Nodules,” “Rheumatoid Nodulosis,” “Rheumatoid Noduloses,” “Rheumatoid Vasculitides,” “Vasculitides, Rheumatoid,” “Vasculitis, Rheumatoid,” “Sjogrens Syndrome,” “Syndrome, Sjogren's,” “Sjogren Syndrome,” “Sicca Syndrome,” “Syndrome, Sicca,” “Still's Disease, Adult-Onset,” “Stills Disease, Adult-Onset,” “Adult-Onset Still's Disease,” “Adult-Onset Still's Disease,” “Adult-Onset Stills Disease,” “Still Disease, Adult-Onset,” “Still Disease, Adult-Onset,” “Adult-Onset Still Disease,” “Adult-Onset Still Disease,” “Methotrexate^*∗*^,” “Amethopterin,” “Methotrexate, (D)-Isomer,” “Methotrexate, (DL)-Isomer,” “Mexate,” “Methotrexate Sodium,” “Sodium, Methotrexate,” “Methotrexate, Sodium Salt,” “Methotrexate, Disodium Salt,” “Methotrexate Hydrate,” “Hydrate, Methotrexate,” “Methotrexate, Dicesium Salt,” and “Dicesium Salt Methotrexate.” Supplementary Information 2 presents the detailed search strategies employed in this study. Unpublished studies and clinical trial registries were also obtained from the databases of GreyNet International, Open Grey, Cochrane Library, and Chinese Clinical Trial Registry.

### 2.4. Study Selection and Data Extractions

The titles and abstracts of the searched results were independently assessed by two investigators (Q. Geng and B. Liu). The full texts of the potentially eligible studies were then screened for final inclusion in the current study. Disagreements were resolved by a third opinion (C. Lu). The extracted data included the study characteristics (authors, title, etc.), patient characteristics (number of patients, age, gender, etc.), intervention, control, and outcomes.

### 2.5. Risk of Bias in Individual Studies

The two investigators (Q. Geng and B. Liu) used the Cochrane Collaboration [22] “Risk of Bias” tool to assess the methodological quality of the each included studies. Seven items including random sequence generation, allocation concealment, blinding of participants and personnel, blinding of outcome assessment, incomplete outcome data, selective reporting, and other biases were assessed as low risk, high risk, or unclear risk. Each potential source of bias was graded as high, low, or unclear.

### 2.6. Statistical Analysis

Statistical analyses were performed using RevMan 5.3 software from the Cochrane Collaboration. The data were summarized using risk ratio (RR) at 95% confidence intervals (CIs) for dichotomous data. For continuous variables, the mean difference (MD) was used if the outcome measurement units of each study were the same, however, standardized MD (SMD) was used if the measurement units and methods were different among the studies. Statistical heterogeneity was tested by examining both the chi-square test and the *I*^*2*^ statistic. The *I*^*2*^ values ranged from 0 to 100% and were categorized as follows: *I*^*2*^ <40%, might not be important; 50% < *I*^*2*^ < 90%, moderate heterogeneity; 75% < *I*^*2*^ < 100%, considerable heterogeneity [23]. A fixed-effect model was used to pool the estimates using the fixed effects model when *I*^*2*^ ≤ 50%,*P* ≥ 0.1. *I*^*2*^ > 50% and *P* < 0.1 indicated the possibility of statistical heterogeneity, and random-effects model was adopted. Potential sources of heterogeneity were explored using subgroup and sensitivity analyses. We conducted a subgroup analysis of different courses and doses (three-month course of 30 mg/day, three-month course of 60 mg/day, six-month course of 30 mg/day, and six-month course of 60 mg/day). The results are presented as forest plots. Sensitivity analyses were also performed to test the stability of the results via the leave-one-out method. Funnel plots and Egger's test were used to assess for publication bias when there are at least 10 studies included in the meta-analysis [22].

### 2.7. Evidence Quality Evaluation

The results of the meta-analysis were evaluated using the GRADE method [24], and degradation was considered in terms of the risk of bias, inconsistency, indirectness, imprecision, and publication bias. The evidence quality was classified as “high quality,” “moderate quality,” “low quality,” and “very low quality.”

## 3. Results

### 3.1. Search Results

**Figure 1:**
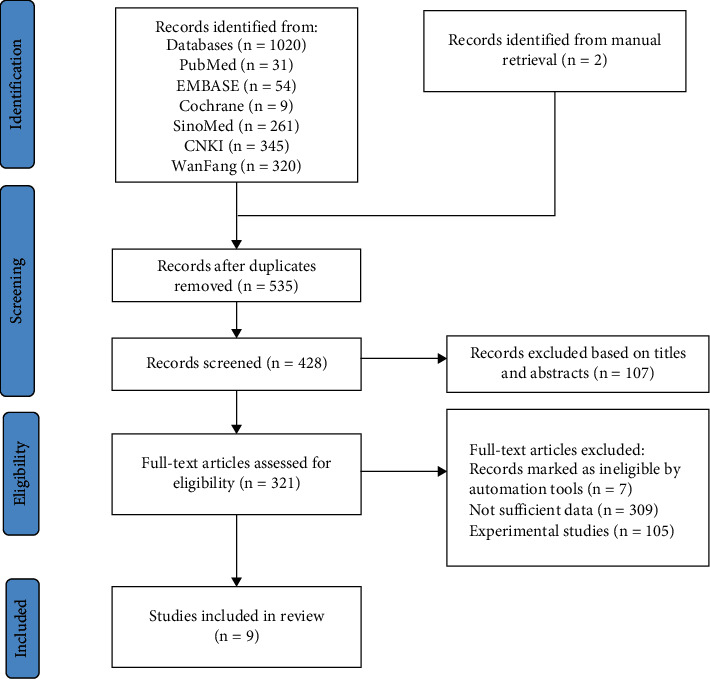
Flow diagram illustrating the process of identifying articles for selection study characteristics.

A total of 1020 articles were identified by literature search: SinoMed (*n* = 258), CNKI (*n* = 330), WanFang Data (*n* = 352), PubMed (*n* = 19), Cochrane Library (*n* = 9), and Embase (*n* = 52). Duplicate checking was conducted using NoteExpress 3.5.0, and 485 papers were ultimately selected. Furthermore, there were 16 reviews and 91 irrelevant studies. After reading through the full articles, the following were excluded from this review: Chinese and English language papers (*n* = 7), duplicate non-TG studies (*n* = 201), studies with primary effect points not meeting ACR20, ACR50, or ACR70 (*n* = 101), experimental studies (*n* = 105), and non-RA studies (*n* = 7). Two records were identified by manual searching. A total of nine RCTs [19, 25–32] were included in the meta-analysis (Figure 1). Supplementary Information 3 shows the list of excluded studies.

The nine trials involving 892 RA participants were all conducted in mainland China. These trials were published from 2013 to 2018. A total of 463 and 429 patients were enrolled in the TG adjuvant MTX therapy and MTX monotherapy groups, respectively. In terms of outcomes, two studies [26, 28] assessed ACR20, one study [32] focused on ACR50, three studies [25, 27, 30] focused on ACR20 and ACR50, and three studies [19, 29, 31] concentrated on ACR20, ACR50, and ACR70. For secondary outcomes, nine studies [19, 25–32] assessed SJC, TJC, ESR, and CRP, and four studies [25, 27, 29, 30] focused on RF (Table 1). TG is available in three- and six-month courses at doses of 30 and 60 mg/day. One study [32] focused on a three-month course at 30 mg/day, and four studies [25, 27, 30] focused on a three-month course at 60 mg/day. At six months of treatment, two studies [26, 28] assessed a dose of 30 mg/day, and two other studies (26, 28) concentrated on a dose of 60 mg/day.

### 3.2. Risk of Bias of the Included RCTs

**Figure 2:**
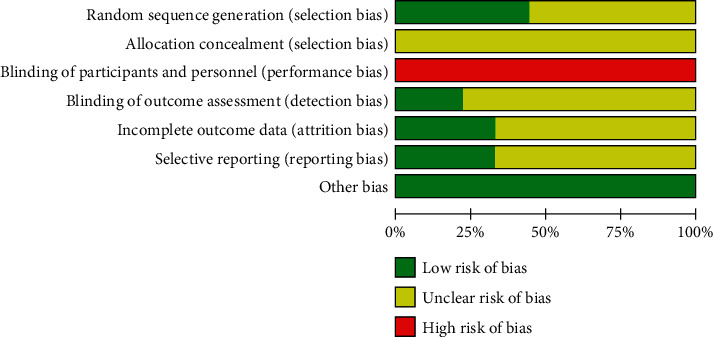
Risk of bias graph.

**Figure 3:**
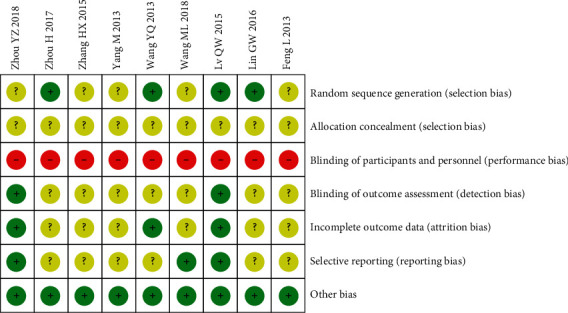
Risk of bias summary.

Four studies [19, 25, 29, 30] had a low risk of bias for random sequence generation because their random number generation method uses a random number table or centralized randomization. The other studies were at unclear risk of random sequence generation because the method was not mentioned in any of these studies. All studies were rated as having an unclear risk for allocation concealment because it was unclear whether the allocation concealment researchers were third-party personnel. With regard to attrition bias, three studies [19, 25, 26] reported the results according to preset outcomes. Thus, these studies were rated as having low risk. The remaining studies failed to clarify whether the outcomes were established in advance. Thus, these studies were rated as having an unclear risk. Two studies [19, 31] were considered at low risk of detection bias, and the remaining studies did not provide blinding information and were considered at unclear risk. Due to the specificity of the intervention, all included studies were considered to be at high risk for performance bias. For reporting bias, the current study had to check the Methods and Results sections of all trials on the bias of the information in the Methods section because of the unavailability of protocols in the included trials. After a rigorous assessment, there was a low risk of selective reporting and other biases in all studies (Figures 2 and 3).

### 3.3. Effects of Interventions with Different Courses and Doses of TG

**Figure 4:**
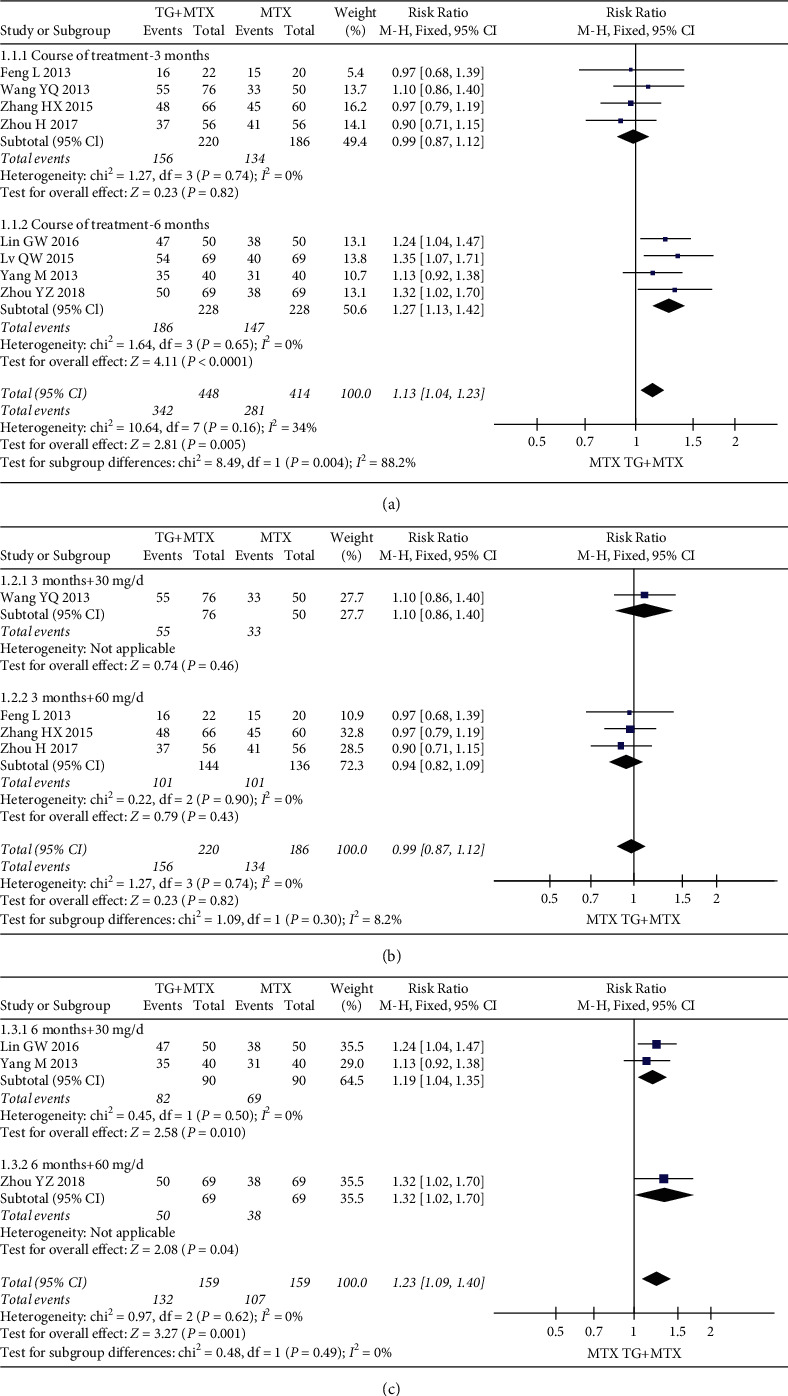
Forest plots for the ACR20 of the different courses and doses of TG adjuvant MTX therapy.

**Figure 5:**
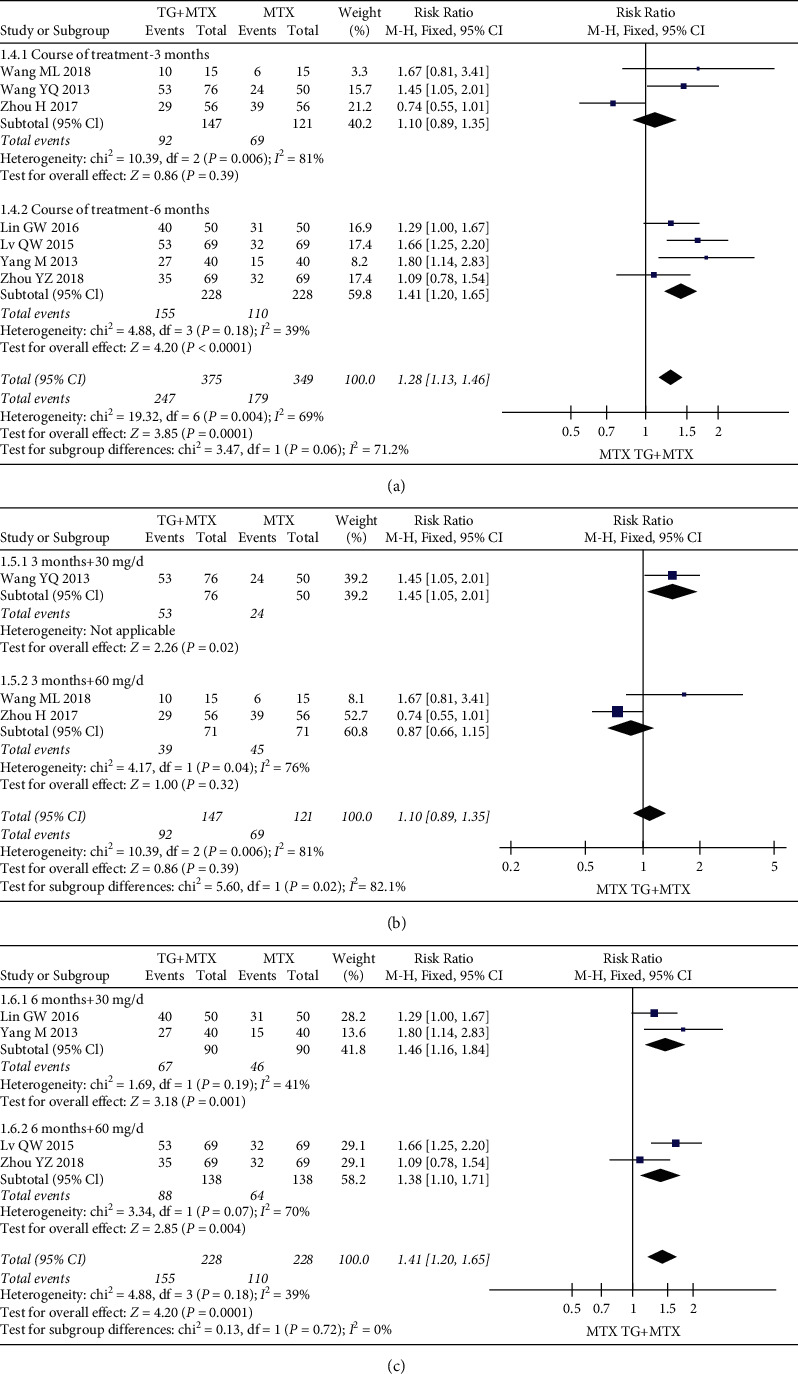
Forest plots for the ACR50 of the different courses and doses of TG adjuvant MTX therapy.

**Figure 6:**
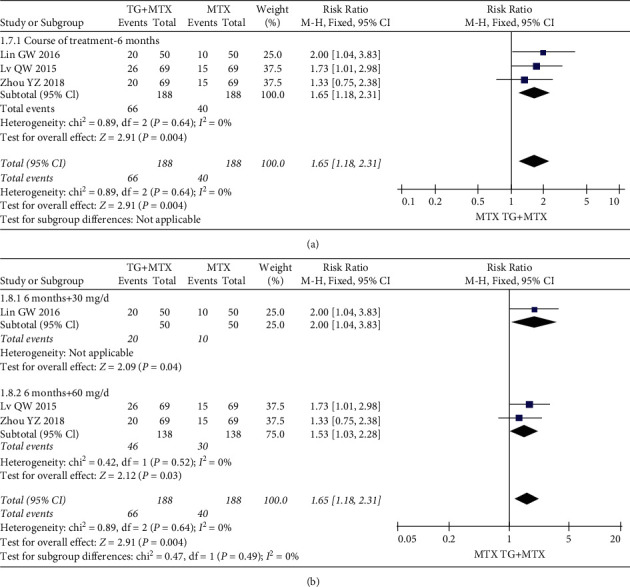
Forest plots for the ACR70 of the different courses and doses of TG adjuvant MTX therapy.

Nine studies evaluated the effects and clinical safety of 892 patients in the TG adjuvant MTX therapy groups and MTX monotherapy therapy groups. For primary outcomes, our analysis revealed that TG adjuvant MTX therapy increased ACR20 (RR = 1.13; 95% CI: [1.04, 1.23]; *P* = 0.005) (figure 4(a)), ACR50 (RR = 1.28; 95% CI: [1.13, 1.46]; *P* = 0.0001) (figure 5(a)), and ACR70 (RR = 1.65; 95% CI: [1.18, 2.31]; *P* = 0.004) (figure 6(a)) responder rates compared with MTX monotherapy.

The response of the TG subgroups for different courses and doses showed that there was no significant improvement in ACR20 for a three-month course (RR = 0.99; 95% CI: [0.87, 1.12]; *P* = 0.82). The forest plot (figure 4(b)) results for a three-month course of 30 mg/day (RR = 1.10; 95% CI: [0.86, 1.40];*P* = 0.46) or 60 mg/day (RR = 0.94; 95% CI: [0.82, 1.09]; *P* = 0.43) showed no statistically significant difference. The results showed that the difference in ACR50 improvement was not statistically significant (RR = 1.10; 95% CI: [0.89, 1.35];*P* = 0.39) in a three-month course, and the results of the 60 mg dose were consistent with the above results (RR = 0.87; 95% CI: [0.66, 1.15]; *P* = 0.32). However, the difference was statistically significant when using a dose of 30 mg (RR = 1.45; 95% CI: [1.05, 2.01]; *P* = 0.02) (figure 5(b)).

Overall, ACR20 improvement after a six-month course of TG treatment (RR = 1.23; 95% CI: [1.09, 1.40];*P* = 0.001) was observed at doses of 30 (RR = 1.19; 95% CI: [1.04, 1.35]; *P* = 0.010) and 60 mg/day (RR = 1.32; 95% CI: [1.02, 1.70];*P* = 0.04) (figure 4(c)).

The forest plot (figure 5(c)) results show the overall ACR50 efficacies (RR = 1.41; 95% CI: [1.20, 1.65]; *P* < 0.0001) for doses of 30 (RR = 1.46; 95% CI: [1.16, 1.84]; *P* = 0.001) and 60 mg/day (RR = 1.38; 95% CI: [1.10, 1.71]; *P* = 0.004). The results were all statistically significant in the overall ACR70 efficacies (RR = 1.65; 95% CI: [1.18, 2.31]; *P* = 0.004) of doses of 30 (RR = 2.00; 95% CI: [1.04, 3.83]; *P* = 0.04) and 60 mg/day (RR = 1.53; 95% CI: [1.03, 2.28]; *P* = 0.03) (figure 6(b)).

For secondary outcomes, it was found that the TG adjuvant MTX therapy reduced the SJC (MD = -2.74; 95% CI: [−3.95, −1.54]; *P* ˂ 0.00001), TJC (MD = −2.63; 95% CI: [−3.56, −1.69]; *P* ˂  0.00001), ESR (MD = -15.71; 95% CI: [−21.40, −10.01]; *P* ˂  0.00001), CRP (SMD = -1.00; 95% CI: [−1.58, −0.42]; *P* = 0.0007), and RF (MD = -45.72; 95% CI: [-74.86, -16.58]; *P* = 0.002) (Supplementary Figure 1).

The overall reductions in SJC (*P* ˂  0.00001), TJC (*P* ˂  0.00001), ESR (*P* ˂  0.00001), CRP (*P* = 0.009), and RF (*P* = 0.0002) were statistically significant in a three-month course of TG treatment. Subgroup analysis showed statistically significant differences regardless of whether the dose was 30 or 60 mg/day (Supplementary Figure 2). After six months, SJC (*P* = 0.004), TJC (*P* = 0.02), ESR (*P* = 0.0005), CRP (*P* = 0.005), and RF (*P* < 0.0001) significantly improved, and the difference was statistically significant (Supplementary Figure 3).

### 3.4. Safety of Interventions

The forest plot (Supplementary Figure 4) showed that there was no significant difference between the safety of MTX monotherapy and TG adjuvant MTX in the occurrence of hepatic adverse effects (RR = 0.71; 95% CI: [0.38, 1.33]; *P* = 0.28), leukopenia (RR = 1.11; 95% CI: [0.55, 2.24]; *P* = 0.78), gastrointestinal adverse effects (RR = 0.83; 95% CI: [0.64, 1.08]; *P* *=* 0.*17*), cutaneous adverse effects (RR = 1.02; 95% CI: [0.57, 1.84]; *P* = 0.94), and irregular menstruation adverse effects (RR = 1.56; 95% CI: [0.69, 3.51]; *P* = 0.29). A heterogeneity test showed that the *I*^*2*^ of each index was less than 50. Hence, the fixed-effects model was adopted.

After three months of treatment, the 30 mg/day dose was not statistically different in terms of safety, including hepatic adverse effects (RR = 0.44; 95% CI: [0.08, 2.53]; *P* = 0.36), leukopenia (RR = 0.33; 95% CI: [0.06, 1.73]; *P* = 0.19), gastrointestinal adverse effects (RR = 0.66; 95% CI: [0.04, 10.28]; *P* *=* 0.77), cutaneous adverse effects (RR = 3.31; 95% CI: [0.16, 67.57]; *P* = 0.44), 60 mg/day dose in terms of leukopenia (RR = 4.56; 95% CI: [0.55, 37.96]; *P* = 0.16), and gastrointestinal adverse effects (RR = 1.52; 95% CI: [0.21, 11.13]; *P* *=* 0.68) (Supplementary Figure 5).

There was no statistical difference in hepatic adverse effects (RR = 1.25; 95% CI: [0.35, 4.50]; *P* = 0.73), leukopenia (RR = 1.00; 95% CI: [0.26, 3.87]; *P* = 1.00), gastrointestinal adverse effects (RR = 0.73; 95% CI: [0.26, 2.10]; *P* *=* 0.*56*), cutaneous adverse effects (RR = 0.75; 95% CI: [0.17, 3.25]; *P* = 0.70), and irregular menstruation adverse effects (RR = 4.00; 95% CI: [0.46, 34.54]; *P* = 0.21) between the six-month course and the 30 mg/day dose. Similar to the result for the 30 mg dose, the result for the 60 mg dose showed that there was no statistical difference in hepatic adverse effects (RR = 0.62; 95% CI: [0.27, 1.39]; *P* = 0.24), leukopenia (RR = 1.25; 95% CI: [0.35, 4.46]; *P* = 0.73), gastrointestinal adverse effects (RR = 0.83; 95% CI: [0.63, 1.09]; *P* *=* 0.*18*), cutaneous adverse effects (RR = 1.00; 95% CI: [0.52, 1.94]; *P* = 1.00), and irregular menstruation adverse effects (RR = 1.25; 95% CI: [0.51, 3.07]; *P* = 0.63) (Supplementary Figure 6).

### 3.5. Sensitivity Analysis

Sensitivity analysis was performed to evaluate the results of different studies. The secondary outcomes of SJC (*I*^*2*^ = 86%), CRP (*I*^*2*^ = 94%), ESR (*I*^*2*^ = 95%), and RF (*I*^*2*^ = 97%) heterogeneity were high. The robustness and variance of SJC, CRP, ESR, and RF were between 73% and 87%, 90% and 94%, 86% and 95%, and 97% and 98%, respectively. All results remained relatively stable according to the leave-one-out sensitivity analysis.

### 3.6. Publication Bias

Egger's tests were performed to evaluate the publication bias of the studies on the primary outcomes of ACR20 (Egger's test: *P* = 0.513) and ACR50 (Egger's test: *P* = 0.539) and on the secondary outcomes of SJC (Egger's test: *P* = 0.555), TJC (Egger's test: *P* = 0.834), CRP (Egger's test: *P* = 0.217), ESR (Egger's test: *P* = 0.05), leukopenia (Egger's test: *P* = 0.250), and gastrointestinal adverse reaction (Egger's test: *P* = 0.844). The symmetrical funnel plot indicated that there was no significant publication bias in this study. These results suggested that there was no significant publication bias in this meta-analysis. In addition, the publication bias could not be assessed for other outcomes because of the small number of studies.

### 3.7. Evidence Quality Evaluation

We used the GRADE Pro system to evaluate the quality of evidence for the primary outcomes of different courses and doses. The RCT was preset to the highest level of evidence in the GRADE evidence quality assessment and was processed according to five degradation factors. The results suggested that the quality of the evidence was low (Supplementary Information 4). The main reason for this result is that the study design is not rigorous, and the sample size is not sufficient.

## 4. Discussion

This study focused on 9 RCTs with 892 RA participants to evaluate the effects and clinical safety of different courses and doses of TG adjuvant MTX therapy in the treatment of RA in comparison with MTX monotherapy. The test results show that there were no significant differences in the baseline of patients and interventions in all evaluated studies. Most system baselines also showed no significant differences, thus conforming to the principle of meta-analysis. The results of this study showed that TG adjuvant MTX therapy is more effective than MTX monotherapy and is a safe strategy for RA treatment with different courses and doses. For effects, a six-month course of TG adjuvant MTX for RA increased the primary outcomes of ACR20, ACR50, and ACR70, which are the gold standard composite measures used in clinical trials of patients with RA [32]. For secondary outcomes, TG adjuvant MTX also reduced the expressions of SJC, TJC, ESR, CRP, and RF. SJC and TJC are indices for evaluating disease activity, severity, and curative effect in patients with RA [33]. ESR and CRP are often used in the clinical diagnosis of RA [34]. RF is a nonspecific detection indicator of RA with high sensitivity [35]. In terms of safety, TG adjuvant MTX therapy did not increase the incidence of adverse effects for three or six months compared with MTX monotherapy. Therefore, these clinical data suggest that a six-month course of treatment at 30 or 60 mg/day of TG adjuvant MTX therapy may be a more effective and safer strategy for RA treatment.

Currently, although the etiology and pathological mechanism of RA are not clear, a large number of studies have shown that the abnormality and interaction of cytokines play important roles in the pathogenesis of RA [36, 37]. Interleukin-6 (IL-6) is a type of proinflammatory cytokine that can promote the proliferation of B cells in RA disease, increase the biological effect of tumor necrosis factor-alpha (TNF-*α*), and promote the development of RA [38]. TNF-*α* is involved in the pathogenesis of RA by activating endothelial cells and promoting the synthesis and release of inflammatory cytokines [39]. Several studies have shown that both MTX monotherapy and TG adjuvant MTX therapy can reduce the expressions of IL-6 and TNF-*α*, however, the expressions of IL-6 and TNF-*α* decrease more significantly in the adjuvant therapy. It suggests that TG adjuvant MTX treatment can enhance the synergistic effect of the two drugs by inhibiting the activities of IL-6 and TNF-*α* and control the progression of RA [40]. This finding may explain the improvement in secondary outcomes at the three-month course even though no significant improvements were observed in ACR20, ACR50, and ACR70. It does not conflict with the absence of improvements in ACR20, ACR50, and ACR70, which is one of the secondary outcomes evaluated for ACR20, ACR50, and ACR70. Significant improvements in ACR20, ACR50, and ACR70 can only be evaluated if three additional indicators improve by more than 20%, 50%, and 70% on top of the improvement in SJC and TJC.

The heterogeneity of secondary outcomes was high. Although we performed subgroup analysis, we were unable to reduce the heterogeneity probably because of the fact that most secondary outcomes were laboratory measures with different reference ranges in different hospitals or probably because there was an uneven distribution among the subgroups in terms of the number of studies and cases.

This meta-analysis has some limitations. Firstly, random allocation principle, allocation concealment, and blinding were not described in detail in some of the included documents. Secondly, given that the sample of raw data in this study was small and because all involved trials were conducted in China, the results of this review might have introduced potential selection bias. It may have caused measurement bias in the implementation and outcome evaluation. Thirdly, the high heterogeneity of individual literature may be because of the low quality of the included literature, the difference in sample size, the difference in disease activity of patients, and the difference in the course of treatment. Although these limitations may undermine the level of evidence of this meta-analysis, the selected trials are highly comparable, and the documents were selected in strict accordance with the inclusion criteria.

## 5. Conclusion

According to the nine RCTs included, a six-month course of TG adjuvant MTX therapy at 30 or 60 mg/day is more effective than MTX monotherapy and is a safe strategy for treating RA. However, because of the low quality of GRADE evaluations and given the limitations of existing research, further high-quality multicenter RCT studies with large sample sizes are needed to confirm the clinical safety of TG combination therapy.

## Figures and Tables

**Table 1:** Characteristics of the included RCTs.

Study ID	Patients	Treatment					Control					Primary outcomes	Secondary outcomes	Course of treatment (months)
		Intervention	N	M/F	Age (Mean ± SD)	Dose(mg/d)	Intervention	N	M/F	Age(Mean ± SD)	Dose(mg/w)			
Wang 2013	Patients with RA	TG + MTX	76	—	43.4 ± 6.6	+TG (30)	MTX	50	—	43.4 ± 6.6	15	①②	④⑤⑥⑦⑧	3
Feng 2013	Patients with RA	TG + MTX	22	2/18	51 ± 4.2	+TG (60)	MTX	20	2/18	52 ± 3.8	10	①	④⑤⑥⑦	3
Yang 2013	Patients with EORA	TG + MTX	40	6/34	69.3 ± 6.9	+TG (30)	MTX	40	4/36	68.8 ± 7.4	10	①②	④⑤⑥⑦⑧	6
Zhang 2015	Patients with EORA	TG + MTX	66	6/54	65 ± 4.2	+TG (60)	MTX	60	6/54	65 ± 3.8	10	①	④⑤⑥⑦	3
Lin 2016	Patients with RA	TG + MTX	50	19/31	44.05 ± 4.9	+TG (30)	MTX	50	20/30	43.7 ± 5.3	10	①②③	④⑤⑥⑦⑧	6
Zhou 2017	Patients with RA	TG + MTX	56	—	45.7 ± 4.8	+TG (60)	MTX	56	—	45.7 ± 4.8	10	①②	④⑤⑥⑦⑧	3
Lv 2015	Patients with RA	TG + MTX	69	14/55	50.6	+TG (60)	MTX	69	10/59	51.0	7.5–12.5	①②③	④⑤⑥⑦	6
Zhou 2018	Patients with RA	TG + MTX	69	14/55	50.6	+TG (60)	MTX	69	10/59	51.0	7.5–12.5	①②③	④⑤⑥⑦	6
Wang 2018	Patients with RA	TG + MTX	15	7/8	55.5	+TG (60)	MTX	15	7/8	53.7	7.5–12.5	②	④⑤⑥⑦	3

Notes: EORA: elderly-onset rheumatoid arthritis ①: ACR20; ②: ACR50; ③: ACR70; ④: SJC; ⑤: TJC; ⑥: ESR; ⑦: CRP; ⑧: RF.

## Data Availability

The data used to support the findings of this study are available from the corresponding author upon request.

## Supplementary Materials

Supplementary Materials Supplementary 1. Supplementary Information 1: items regarding the PRISMA checklist for network meta-analysis. Supplementary Information 2: detailed search strategies. Supplementary Information 3: a list of all excluded papers. Supplementary 2Supplementary Information 4: quality assessment using the GRADE approach. Supplementary Figure 1: forest plots for the secondary outcomes of TG adjuvant MTX therapy. Supplementary Figure 2: forest plots for the secondary outcomes of a three-month course of TG adjuvant MTX therapy at a dose of 30 mg/day. Supplementary Figure 3: forest plots for the secondary outcomes of the different courses and doses of TG adjuvant MTX therapy. Supplementary Figure 4: forest plots for the safety of TG adjuvant MTX therapy. Supplementary Figure 5: forest plots for the safety of a three-month course of TG adjuvant MTX therapy at the dose of 30 mg/day. Supplementary Figure 6: forest plots for the safety of the different courses and doses of TG adjuvant MTX therapy.
