# Supplementary material for: Effects and Safety of the Tripterygium Glycoside Adjuvant Methotrexate Therapy in Rheumatoid Arthritis: A Systematic Review and Meta-Analysis
Source: Evid Based Complement Alternat Med. 2022 Mar 24;2022:1251478. doi: 10.1155/2022/1251478 (PMC8970871; doi:10.1155/2022/1251478)
Supplement: Supplementary Materials — Supplementary 1. Supplementary Information 1: items regarding the PRISMA checklist for network meta-analysis. Supplementary Information 2: detailed search strategies. Supplementary Information 3: a list of all excluded papers. Supplementary 2Supplementary Information 4: quality assessment using the GRADE approach. Supplementary Figure 1: forest plots for the secondary outcomes of TG adjuvant MTX therapy. Supplementary Figure 2: forest plots for the secondary outcomes of a three-month course of TG adjuvant MTX therapy at a dose of 30 mg/day. Supplementary Figure 3: forest plots for the secondary outcomes of the different courses and doses of TG adjuvant MTX therapy. Supplementary Figure 4: forest plots for the safety of TG adjuvant MTX therapy. Supplementary Figure 5: forest plots for the safety of a three-month course of TG adjuvant MTX therapy at the dose of 30 mg/day. Supplementary Figure 6: forest plots for the safety of the different courses and doses of TG adjuvant MTX therapy. [file 1251478.f1.zip › 1251478.f1/Supplementary Information 2 (3).docx]

**Supplementary Information 2.**

**The search strategy of SinoMed (n=261)**

1. “Arthritis, Rheumatoid” [Unweighted: extension]
2. “Caplan Syndrome” [Unweighted: extension]
3. “Felty Syndrome” [Unweighted: extension]
4. “Rheumatoid Nodule” [Unweighted: extension]
5. “Sjogren Syndrome” [Unweighted: extension]
6. “Adult onset still disease” [Unweighted: extension]
7. “Rheumatoid vasculitis” [Unweighted: extension]
8. “Rheumatoid Arthritis” [Common fields: intelligent]
9. “Caplan Syndrome” [Common fields: intelligent]
10. “Felty Syndrome” [Common fields: intelligent]
11. “Sjogren Syndrome” [Common fields: intelligent]
12. OR/1-11
13. “Tripterygium” [Unweighted: extension]
14. “Tripterygium hypoglaucum” [Common fields: intelligent]
15. “Tripterygium glycoside” [Common fields: intelligent]
16. “Tripterygium wilfordii” [Common fields: intelligent]
17. “Tripterygium glycosides” [Common fields: intelligent]
18. “Tripterygium wilfordius” [Common fields: intelligent]
19. OR/13-18
20. “Methotrexate” [Unweighted: extension]
21. “Amethopterin” [Common fields: intelligent]
22. OR/20-21
23. #12 AND #19 AND #22

**The search strategy of CNKI (n=345)**

1. “Arthritis, Rheumatoid” [Topic]
2. “Caplan Syndrome” [Topic]
3. “Felty Syndrome” [Topic]
4. “Rheumatoid Nodule” [Topic]
5. “Sjogren Syndrome” [Topic]
6. “Adult onset still disease” [Topic]
7. “Rheumatoid vasculitis” [Topic]
8. “Rheumatoid Arthritis” [Topic]
9. “Caplan Syndrome” [Topic]
10. “Felty Syndrome” [Topic]
11. “Sjogren Syndrome” [Topic]
12. OR/1-11
13. “Tripterygium” [Topic]
14. “Tripterygium hypoglaucum” [Topic]
15. “Tripterygium glycoside” [Topic]
16. “Tripterygium wilfordii” [Topic]
17. “Tripterygium glycosides” [Topic]
18. “Tripterygium wilfordius” [Topic]
19. OR/13-18
20. “Methotrexate” [Topic]
21. “Amethopterin” [Topic]
22. OR/20-21
23. #12 AND #19 AND #22

**The search strategy of WanFang Data (n=320)**

1. “Arthritis, Rheumatoid” [Topic]
2. “Caplan Syndrome” [Topic]
3. “Felty Syndrome” [Topic]
4. “Rheumatoid Nodule” [Topic]
5. “Sjogren Syndrome” [Topic]
6. “Adult onset still disease” [Topic]
7. “Rheumatoid vasculitis” [Topic]
8. “Rheumatoid Arthritis” [Topic]
9. “Caplan Syndrome” [Topic]
10. “Felty Syndrome” [Topic]
11. “Sjogren Syndrome” [Topic]
12. OR/1-11
13. “Tripterygium” [Topic]
14. “Tripterygium hypoglaucum” [Topic]
15. “Tripterygium glycoside” [Topic]
16. “Tripterygium wilfordii” [Topic]
17. “Tripterygium glycosides” [Topic]
18. “Tripterygium wilfordius” [Topic]
19. OR/13-18
20. “Methotrexate” [Topic]
21. “Amethopterin” [Topic]
22. OR/20-21
23. #12 AND #19 AND #22

**The search strategy of PubMed (n=31)**

1. “Arthritis, Rheumatoid” [MeSH]
2. “Caplan Syndrome” [MeSH]
3. “Felty Syndrome” [MeSH]
4. “Rheumatoid Nodule” [MeSH]
5. “Rheumatoid Vasculitis” [MeSH]
6. “Sjogren's Syndrome” [MeSH]
7. “Still's Disease, Adult-Onset” [MeSH]
8. “Rheumatoid Arthritis” [Title/Abstract]
9. “Caplan Syndrome” [Title/Abstract]
10. “Caplan's Syndrome” [Title/Abstract]
11. “Caplans Syndrome” [Title/Abstract]
12. “Syndrome, Felty” [Title/Abstract]
13. “Syndrome, Felty's” [Title/Abstract]
14. “Familial Felty's Syndrome” [Title/Abstract]
15. “Familial Feltys Syndrome” [Title/Abstract]
16. “Felty's Syndrome, Familial” [Title/Abstract]
17. “Syndrome, Familial Felty's” [Title/Abstract]
18. “Rheumatoid Arthritis, Splenomegaly and Neutropenia” [Title/Abstract]
19. “Familial Felty Syndrome” [Title/Abstract]
20. “Felty Syndrome, Familial” [Title/Abstract]
21. “Syndrome, Familial Felty” [Title/Abstract]
22. “Nodule, Rheumatoid” [Title/Abstract]
23. “Nodules, Rheumatoid” [Title/Abstract]
24. “Rheumatoid Nodules” [Title/Abstract]
25. “Rheumatoid Nodulosis” [Title/Abstract]
26. “Rheumatoid Noduloses” [Title/Abstract]
27. “Rheumatoid Vasculitides” [Title/Abstract]
28. “Vasculitides, Rheumatoid” [Title/Abstract]
29. “Vasculitis, Rheumatoid” [Title/Abstract]
30. “Sjogrens Syndrome” [Title/Abstract]
31. “Syndrome, Sjogren's” [Title/Abstract]
32. “Sjogren Syndrome” [Title/Abstract]
33. “Sicca Syndrome” [Title/Abstract]
34. “Syndrome, Sicca” [Title/Abstract]
35. “Still's Disease, Adult Onset” [Title/Abstract]
36. “Stills Disease, Adult-Onset” [Title/Abstract]
37. “Adult-Onset Still's Disease” [Title/Abstract]
38. “Adult Onset Still's Disease” [Title/Abstract]
39. “Adult-Onset Stills Disease” [Title/Abstract]
40. “Still Disease, Adult-Onset” [Title/Abstract]
41. “Still Disease, Adult Onset” [Title/Abstract]
42. “Adult-Onset Still Disease” [Title/Abstract]
43. “Adult Onset Still Disease” [Title/Abstract]
44. OR/1-43
45. “Tripterygium” [MeSH]
46. “Tripterygium hypoglaucum” [Title/Abstract]
47. “Tripterygium hypoglaucums” [Title/Abstract]
48. “hypoglaucums, Tripterygium” [Title/Abstract]
49. “Tripterygium wilfordii” [Title/Abstract]
50. “Tripterygium wilfordius” [Title/Abstract]
51. “wilfordius, Tripterygium” [Title/Abstract]
52. “Leigong Teng” [Title/Abstract]
53. “Leigong Tengs” [Title/Abstract]
54. “Teng, Leigong” [Title/Abstract]
55. “Tengs, Leigong” [Title/Abstract]
56. “Thundergod Vine” [Title/Abstract]
57. “Thundergod Vines” [Title/Abstract]
58. “Vine, Thundergod” [Title/Abstract]
59. “Vines, Thundergod” [Title/Abstract]
60. OR/45-59
61. “Methotrexate” [MeSH]
62. “Methotrexate, (D)-Isomer” [Title/Abstract]
63. “Methotrexate, (DL)-Isomer” [Title/Abstract]
64. “Mexate” [Title/Abstract]
65. “Methotrexate Sodium” [Title/Abstract]
66. “Sodium, Methotrexate” [Title/Abstract]
67. “Methotrexate, Sodium Salt” [Title/Abstract]
68. “Methotrexate, Disodium Salt” [Title/Abstract]
69. “Methotrexate Hydrate” [Title/Abstract]
70. “Hydrate, Methotrexate” [Title/Abstract]
71. “Methotrexate, Dicesium Salt” [Title/Abstract]
72. “Dicesium Salt Methotrexate” [Title/Abstract]
73. OR/61-72
74. #44 AND #60 AND #73

**The search strategy of Cochrane (n=9)**

1. “Arthritis, Rheumatoid” [MeSH]
2. “Caplan Syndrome” [MeSH]
3. “Felty Syndrome” [MeSH]
4. “Rheumatoid Nodule” [MeSH]
5. “Rheumatoid Vasculitis” [MeSH]
6. “Sjogren's Syndrome” [MeSH]
7. “Still's Disease, Adult-Onset” [MeSH]
8. “Rheumatoid Arthritis” [Title/Abstract]
9. “Caplan Syndrome”: ti,ab,kw
10. “Caplan's Syndrome”: ti,ab,kw
11. “Caplans Syndrome”: ti,ab,kw
12. “Syndrome, Felty”: ti,ab,kw
13. “Syndrome, Felty's”: ti,ab,kw
14. “Familial Felty's Syndrome”: ti,ab,kw
15. “Familial Feltys Syndrome”: ti,ab,kw
16. “Felty's Syndrome, Familial”: ti,ab,kw
17. “Syndrome, Familial Felty's”: ti,ab,kw
18. “Rheumatoid Arthritis, Splenomegaly and Neutropenia”: ti,ab,kw
19. “Familial Felty Syndrome”: ti,ab,kw
20. “Felty Syndrome, Familial”: ti,ab,kw
21. “Syndrome, Familial Felty”: ti,ab,kw
22. “Nodule, Rheumatoid”: ti,ab,kw
23. “Nodules, Rheumatoid”: ti,ab,kw
24. “Rheumatoid Nodules”: ti,ab,kw
25. “Rheumatoid Nodulosis”: ti,ab,kw
26. “Rheumatoid Noduloses”: ti,ab,kw
27. “Rheumatoid Vasculitides”: ti,ab,kw
28. “Vasculitides, Rheumatoid”: ti,ab,kw
29. “Vasculitis, Rheumatoid”: ti,ab,kw
30. “Sjogrens Syndrome”: ti,ab,kw
31. “Syndrome, Sjogren's”: ti,ab,kw
32. “Sjogren Syndrome”: ti,ab,kw
33. “Sicca Syndrome”: ti,ab,kw
34. “Syndrome, Sicca”: ti,ab,kw
35. “Still's Disease, Adult Onset”: ti,ab,kw
36. “Stills Disease, Adult-Onset”: ti,ab,kw
37. “Adult-Onset Still's Disease”: ti,ab,kw
38. “Adult Onset Still's Disease”: ti,ab,kw
39. “Adult-Onset Stills Disease”: ti,ab,kw
40. “Still Disease, Adult-Onset”: ti,ab,kw
41. “Still Disease, Adult Onset”: ti,ab,kw
42. “Adult-Onset Still Disease”: ti,ab,kw
43. “Adult Onset Still Disease”: ti,ab,kw
44. OR/1-43
45. “Tripterygium” [MeSH]
46. “Tripterygium hypoglaucum”: ti,ab,kw
47. “Tripterygium hypoglaucums”: ti,ab,kw
48. “hypoglaucums, Tripterygium”: ti,ab,kw
49. “Tripterygium wilfordii”: ti,ab,kw
50. “Tripterygium wilfordius”: ti,ab,kw
51. “wilfordius, Tripterygium”: ti,ab,kw
52. “Leigong Teng”: ti,ab,kw
53. “Leigong Tengs”: ti,ab,kw
54. “Teng, Leigong”: ti,ab,kw
55. “Tengs, Leigong”: ti,ab,kw
56. “Thundergod Vine”: ti,ab,kw
57. “Thundergod Vines”: ti,ab,kw
58. “Vine, Thundergod”: ti,ab,kw
59. “Vines, Thundergod”: ti,ab,kw
60. OR/45-59
61. “Methotrexate” [MeSH]
62. “Methotrexate, (D)-Isomer”: ti,ab,kw
63. “Methotrexate, (DL)-Isomer”: ti,ab,kw
64. “Mexate”: ti,ab,kw
65. “Methotrexate Sodium”: ti,ab,kw
66. “Sodium, Methotrexate”: ti,ab,kw
67. “Methotrexate, Sodium Salt”: ti,ab,kw
68. “Methotrexate, Disodium Salt”: ti,ab,kw
69. “Methotrexate Hydrate”: ti,ab,kw
70. “Hydrate, Methotrexate”: ti,ab,kw
71. “Methotrexate, Dicesium Salt”: ti,ab,kw
72. “Dicesium Salt Methotrexate”: ti,ab,kw
73. OR/61-72
74. #44 AND #60 AND #73

**The search strategy of Embase (n=54)**

1. “Arthritis, Rheumatoid” /exp
2. “Caplan Syndrome” /exp
3. “Felty Syndrome” /exp
4. “Rheumatoid Nodule” /exp
5. “Rheumatoid Vasculitis” /exp
6. “Sjogren's Syndrome” /exp
7. “Still's Disease, Adult-Onset” /exp
8. “Rheumatoid Arthritis”: ab,ti,kw
9. “Caplan Syndrome”: ab,ti,kw
10. “Caplan's Syndrome”: ab,ti,kw
11. “Caplans Syndrome”: ab,ti,kw
12. “Syndrome, Felty”: ab,ti,kw
13. “Syndrome, Felty's”: ab,ti,kw
14. “Familial Felty's Syndrome”: ab,ti,kw
15. “Familial Feltys Syndrome”: ab,ti,kw
16. “Felty's Syndrome, Familial”: ab,ti,kw
17. “Syndrome, Familial Felty's”: ab,ti,kw
18. “Rheumatoid Arthritis, Splenomegaly and Neutropenia”: ab,ti,kw
19. “Familial Felty Syndrome”: ab,ti,kw
20. “Felty Syndrome, Familial”: ab,ti,kw
21. “Syndrome, Familial Felty”: ab,ti,kw
22. “Nodule, Rheumatoid”: ab,ti,kw
23. “Nodules, Rheumatoid”: ab,ti,kw
24. “Rheumatoid Nodules”: ab,ti,kw
25. “Rheumatoid Nodulosis”: ab,ti,kw
26. “Rheumatoid Noduloses”: ab,ti,kw
27. “Rheumatoid Vasculitides”: ab,ti,kw
28. “Vasculitides, Rheumatoid”: ab,ti,kw
29. “Vasculitis, Rheumatoid”: ab,ti,kw
30. “Sjogrens Syndrome”: ab,ti,kw
31. “Syndrome, Sjogren's”: ab,ti,kw
32. “Sjogren Syndrome”: ab,ti,kw
33. “Sicca Syndrome”: ab,ti,kw
34. “Syndrome, Sicca”: ab,ti,kw
35. “Still's Disease, Adult Onset”: ab,ti,kw
36. “Stills Disease, Adult-Onset”: ab,ti,kw
37. “Adult-Onset Still's Disease”: ab,ti,kw
38. “Adult Onset Still's Disease”: ab,ti,kw
39. “Adult-Onset Stills Disease”: ab,ti,kw
40. “Still Disease, Adult-Onset”: ab,ti,kw
41. “Still Disease, Adult Onset”: ab,ti,kw
42. “Adult-Onset Still Disease”: ab,ti,kw
43. “Adult Onset Still Disease”: ab,ti,kw
44. OR/1-43
45. “Tripterygium” /exp
46. “Tripterygium hypoglaucum”: ab,ti,kw
47. “Tripterygium hypoglaucums”: ab,ti,kw
48. “hypoglaucums, Tripterygium”: ab,ti,kw
49. “Tripterygium wilfordii”: ab,ti,kw
50. “Tripterygium wilfordius”: ab,ti,kw
51. “wilfordius, Tripterygium”: ab,ti,kw
52. “Leigong Teng”: ab,ti,kw
53. “Leigong Tengs”: ab,ti,kw
54. “Teng, Leigong”: ab,ti,kw
55. “Tengs, Leigong”: ab,ti,kw
56. “Thundergod Vine”: ab,ti,kw
57. “Thundergod Vines”: ab,ti,kw
58. “Vine, Thundergod”: ab,ti,kw
59. “Vines, Thundergod”: ab,ti,kw
60. OR/45-59
61. “Methotrexate” /exp
62. “Methotrexate, (D)-Isomer”: ab,ti,kw
63. “Methotrexate, (DL)-Isomer”: ab,ti,kw
64. “Mexate”: ab,ti,kw
65. “Methotrexate Sodium”: ab,ti,kw
66. “Sodium, Methotrexate”: ab,ti,kw
67. “Methotrexate, Sodium Salt”: ab,ti,kw
68. “Methotrexate, Disodium Salt”: ab,ti,kw
69. “Methotrexate Hydrate”: ab,ti,kw
70. “Hydrate, Methotrexate”: ab,ti,kw
71. “Methotrexate, Dicesium Salt”: ab,ti,kw
72. “Dicesium Salt Methotrexate”: ab,ti,kw
73. OR/61-72
74. #44 AND #60 AND #73
