# Supplementary material for: Effects and Safety of the Tripterygium Glycoside Adjuvant Methotrexate Therapy in Rheumatoid Arthritis: A Systematic Review and Meta-Analysis
Source: Evid Based Complement Alternat Med. 2022 Mar 24;2022:1251478. doi: 10.1155/2022/1251478 (PMC8970871; doi:10.1155/2022/1251478)
Supplement: Supplementary Materials — Supplementary 1. Supplementary Information 1: items regarding the PRISMA checklist for network meta-analysis. Supplementary Information 2: detailed search strategies. Supplementary Information 3: a list of all excluded papers. Supplementary 2Supplementary Information 4: quality assessment using the GRADE approach. Supplementary Figure 1: forest plots for the secondary outcomes of TG adjuvant MTX therapy. Supplementary Figure 2: forest plots for the secondary outcomes of a three-month course of TG adjuvant MTX therapy at a dose of 30 mg/day. Supplementary Figure 3: forest plots for the secondary outcomes of the different courses and doses of TG adjuvant MTX therapy. Supplementary Figure 4: forest plots for the safety of TG adjuvant MTX therapy. Supplementary Figure 5: forest plots for the safety of a three-month course of TG adjuvant MTX therapy at the dose of 30 mg/day. Supplementary Figure 6: forest plots for the safety of the different courses and doses of TG adjuvant MTX therapy. [file 1251478.f1.zip › 1251478.f1/Supplementary Information 3 (2).docx]

**A citation list of all the excluded papers**

**485 repeat studies**

[1]. 吴庆军等, 5例抗丙氨酰tRNA合成酶抗体阳性患者临床特征. 中华临床免疫和变态反应杂志, 2014. 8(02): 第129-133页.

[2]. 龙武彬等, 99Tc-MDP治疗类风湿关节炎30例. 中国药业, 2002. 11(5): 第74-75页.

[3]. 李宗英, BLyS、MCP-1在类风湿关节炎中的表达及其与肺间质纤维化的关系研究, 2013, 河北医科大学.

[4]. Wu, Y.J., Z.Y. Lao and Z.L. Zhang, Clinical observation on small doses Tripterygium wilfordii polyglycoside combined with methotrexate in treating rheumatoid arthritis. Zhongguo Zhong xi yi jie he za zhi Zhongguo Zhongxiyi jiehe zazhi = Chinese journal of integrated traditional and Western medicine / Zhongguo Zhong xi yi jie he xue hui, Zhongguo Zhong yi yan jiu yuan zhu ban, 2001. 21(12): p. 895-896.

[5]. 田伟兰等, MTX联合不同中药对Ⅱ型胶原诱导关节炎大鼠血清TNF-α及RORγt mRNA表达的影响. 中华中医药杂志, 2016. 31(10): 第4184-4187页.

[6]. 刘汉顺, RA中医证型与影像学相关性的研究, 2008, 贵阳中医学院.

[7]. 钱瑾, 阿达木单抗联合雷公藤多甙对甲氨蝶呤治疗反应不佳活动性类风湿关节炎的效果及安全性分析. 中西医结合心血管病电子杂志, 2019. 7(35): 第33-34页.

[8]. 夏楠楠, 陈忠锋与张伟峰, 艾拉莫德与雷公藤多苷片治疗类风湿关节炎的效果观察. 实用中西医结合临床, 2020. 20(11): 第76-77页.

[9]. 夏楠楠, 陈忠锋与张伟峰, 艾拉莫德与雷公藤多苷片治疗类风湿关节炎的效果观察. 实用中西医结合临床, 2020. 20(11): 第76-77页.

[10]. 李兴锐, 陈茂红与王和融, 白芍总苷合雷公藤多苷治疗类风湿关节炎60例. 安徽中医学院学报, 2011. 30(03): 第16-18页.

[11]. 李兴锐, 陈茂红与王和融, 白芍总苷合雷公藤多苷治疗类风湿关节炎60例. 安徽中医学院学报, 2011. 30(3): 第16-18页.

[12]. 许武, 白芍总苷胶囊联合雷公藤片治疗类风湿关节炎疗效和安全性分析. 现代诊断与治疗, 2017. 28(13): 第2404-2406页.

[13]. 许武, 白芍总苷胶囊联合雷公藤片治疗类风湿关节炎疗效和安全性分析. 现代诊断与治疗, 2017. 28(13): 第2404-2406页.

[14]. 吕倩雯与张烜, 比较雷公藤多甙和甲氨蝶呤对治疗活动性类风湿关节炎的有效性与安全性. 中华内科杂志, 2015. 54(06): 第537页.

[15]. 吕倩雯与张烜, 比较雷公藤多甙和甲氨蝶呤对治疗活动性类风湿关节炎的有效性与安全性. 中华内科杂志, 2015. 54(6): 第537页.

[16]. 曾祯, 辨证治疗类风湿关节炎76例. 中医杂志, 2010. 51(S2): 第193-194页.

[17]. 曾祯, 辨证治疗类风湿关节炎76例. 中国民族民间医药, 2010. 19(15): 第171-171页.

[18]. 梁启迪, 段勇明与秦超, 补益肝肾汤联合常规疗法对类风湿性关节炎患者血清炎症因子及生活质量的影响. 四川中医, 2019. 37(07): 第143-146页.

[19]. 梁启迪, 段勇明与秦超, 补益肝肾汤联合常规疗法对类风湿性关节炎患者血清炎症因子及生活质量的影响. 四川中医, 2019. 37(7): 第143-146页.

[20]. 林伟耿, 林秋强与陈韧, 不同治疗方案对类风湿关节炎的效果观察. 中国当代医药, 2016. 23(16): 第4-7+11页.

[21]. 林伟耿, 林秋强与陈韧, 不同治疗方案对类风湿关节炎的效果观察. 中国当代医药, 2016. 23(16): 第4-7,11页.

[22]. 姜慧晶, 除痹汤联合膝关节围刺对寒热错杂型类风湿性关节炎疗效观察. 中国中医药现代远程教育, 2020. 18(04): 第319-321页.

[23]. 姜慧晶, 除痹汤联合膝关节围刺对寒热错杂型类风湿性关节炎疗效观察. 中国中医药现代远程教育, 2020. 18(4): 第后插143-后插145页.

[24]. 巴鑫, 陈哲与涂胜豪, 从表观遗传学角度看雷公藤治疗类风湿关节炎的研究进展. 中华风湿病学杂志, 2018. 22(11): 第785-788页.

[25]. 周腊梅等, 从病例分析RA慢作用药物治疗方案选择. 西藏科技, 2017(03): 第38-41页.

[26]. 周腊梅等, 从病例分析RA慢作用药物治疗方案选择. 西藏科技, 2017(3): 第38-41页.

[27]. 益西拉姆等, 从病例分析RA药物的临床应用. 西藏科技, 2017(02): 第53-56页.

[28]. 李玲与王莘智, 从寒热辨证探讨雷公藤片对类风湿性关节炎的临床疗效. 亚太传统医药, 2020. 16(03): 第140-143页.

[29]. 李玲与王莘智, 从寒热辨证探讨雷公藤片对类风湿性关节炎的临床疗效. 亚太传统医药, 2020. 16(3): 第140-143页.

[30]. 卢军, 断藤益母汤及其组方药物对胶原诱导性关节炎大鼠骨代谢的影响及其机制研究, 2016, 广州中医药大学.

[31]. 杨通宇等, 飞金止痛胶囊联合甲氨蝶呤和雷公藤多苷治疗类风湿关节炎的临床观察. 中国药房, 2016. 27(23): 第3218-3220页.

[32]. 杨通宇等, 飞金止痛胶囊联合甲氨蝶呤和雷公藤多苷治疗类风湿关节炎的临床观察. 中国药房, 2016. 27(23): 第3218-3220页.

[33]. 高泽林与王新宏, 肺间质病变32例临床分析. 陕西医学杂志, 2007(08): 第1033-1034页.

[34]. 高泽林与王新宏, 肺间质病变32例临床分析. 陕西医学杂志, 2007. 36(8): 第1033-1034页.

[35]. 张磊, 风湿骨痛贴方治疗类风湿关节炎临床研究, 2016, 河北大学.

[36]. 许建民等, 风湿性多肌痛住院患者38例临床诊治分析. 中华老年多器官疾病杂志, 2012. 11(12): 第893-896页.

[37]. 黄丽军等, 蜂针治疗类风湿关节炎Meta分析. 中华中医药学刊, 2017. 35(05): 第1211-1215页.

[38]. 黄丽军等, 蜂针治疗类风湿关节炎Meta分析. 中华中医药学刊, 2017. 35(5): 第1211-1215页.

[39]. 陈岳祥等, 复方粉背雷公藤治疗类风湿关节炎的临床观察. 华南国防医学杂志, 2009. 23(02): 第4-5+11页.

[40]. 陈岳祥等, 复方粉背雷公藤治疗类风湿关节炎的临床观察. 华南国防医学杂志, 2009. 23(2): 第4-5,11页.

[41]. 孟彪, 高立珍与赵和平, 复方雷公藤药酒治疗类风湿关节炎(寒湿痹阻证)的临床观察. 中医药信息, 2013. 30(01): 第94-95页.

[42]. 孟彪, 高立珍与赵和平, 复方雷公藤药酒治疗类风湿关节炎(寒湿痹阻证)的临床观察. 中医药信息, 2013. 30(1): 第94-95页.

[43]. 戴洁梅等, 复方雷公藤逐痛颗粒辅助治疗痰瘀互结型类风湿关节炎的临床效果观察. 现代生物医学进展, 2019. 19(04): 第743-746页.

[44]. 戴洁梅等, 复方雷公藤逐痛颗粒辅助治疗痰瘀互结型类风湿关节炎的临床效果观察. 现代生物医学进展, 2019. 19(4): 第743-746页.

[45]. 陈利锋等, 复方芪芎颗粒对佐剂性关节炎大鼠抗炎作用的实验研究. 华南国防医学杂志, 2015. 29(06): 第415-418页.

[46]. 陈利锋等, 复方芪芎颗粒对佐剂性关节炎大鼠抗炎作用的实验研究. 华南国防医学杂志, 2015. 29(6): 第415-418页.

[47]. 刘婧依等, 戈利木单抗联合雷公藤多甙对甲氨蝶呤治疗反应不佳活动性类风湿关节炎的效果及安全性分析. 现代生物医学进展, 2017. 17(09): 第1735-1738+1725页.

[48]. 刘婧依等, 戈利木单抗联合雷公藤多甙对甲氨蝶呤治疗反应不佳活动性类风湿关节炎的效果及安全性分析. 现代生物医学进展, 2017. 17(9): 第1735-1738,1725页.

[49]. 康尔恂等, 关节病性银屑病52例临床分析. 临床皮肤科杂志, 2004(11): 第665-667页.

[50]. 康尔恂等, 关节病性银屑病52例临床分析. 临床皮肤科杂志, 2004. 33(11): 第665-667页.

[51]. 金香花, 刘畅与雷蕾, 观察比较两种类风湿关节炎治疗方案的疗效及安全性. 中国实用医药, 2020. 15(26): 第153-154页.

[52]. 金香花, 刘畅与雷蕾, 观察比较两种类风湿关节炎治疗方案的疗效及安全性. 中国实用医药, 2020. 15(26): 第153-154页.

[53]. 陈霸琼, 桂枝芍药知母汤加减方治疗类风湿关节炎寒热错杂证的临床研究, 2012, 南京中医药大学.

[54]. 向永国, 桂枝芍药知母汤加味治疗类风湿性关节炎疗效观察. 四川中医, 2016. 34(07): 第111-113页.

[55]. 向永国, 桂枝芍药知母汤加味治疗类风湿性关节炎疗效观察. 四川中医, 2016. 34(7): 第111-113页.

[56]. 陈用军与段逸群. 桂枝芍药知母汤抗炎及免疫调节作用机制. in 中华中医药学会皮肤科分会第四次学术年会;全国中医、中西医结合皮肤病诊疗新进展高级研修班. 2007. 中国新疆乌鲁木齐.

[57]. 陈用军, 桂枝芍药知母汤抗炎及免疫调节作用机制的实验研究, 2007, 湖北中医药大学;湖北中医学院.

[58]. 赵文甲, 刘维与吴沅皞, 桂枝芍药知母汤治疗类风湿关节炎疗效及安全性的Meta分析. 中国老年学杂志, 2018. 38(18): 第4435-4440页.

[59]. 赵文甲, 刘维与吴沅皞, 桂枝芍药知母汤治疗类风湿关节炎疗效及安全性的Meta分析. 中国老年学杂志, 2018. 38(18): 第4435-4440页.

[60]. 郭春霞, 齐静与赵敏, 过量服用甲氨蝶呤致严重口腔溃疡并发真菌感染1例. 华北煤炭医学院学报, 2006. 8(1): 第3-3页.

[61]. 阎晓霞, 赵志强与仝允辉, 化瘀通痹熏蒸方联合西药治疗类风湿关节炎30例. 中医研究, 2015. 28(04): 第17-19页.

[62]. 阎晓霞, 赵志强与仝允辉, 化瘀通痹熏蒸方联合西药治疗类风湿关节炎30例. 中医研究, 2015(4): 第17-19页.

[63]. 达其伟等, 黄芪桂枝五物汤加减内服、熏蒸联合西药治疗类风湿关节炎30例. 中医研究, 2015. 28(09): 第10-13页.

[64]. 达其伟等, 黄芪桂枝五物汤加减内服、熏蒸联合西药治疗类风湿关节炎30例. 中医研究, 2015. 28(9): 第10-13页.

[65]. 陈利锋等, 黄芪注射液合川芎嗪注射液治疗类风湿关节炎的作用机理研究. 中国中医急症, 2012. 21(06): 第924-925+928页.

[66]. 陈利锋等, 黄芪注射液合川芎嗪注射液治疗类风湿关节炎的作用机理研究. 中国中医急症, 2012. 21(6): 第924-925,928页.

[67]. 徐婧, 石连杰与张学武, 肌内注射甲氨蝶呤治疗类风湿关节炎合并短肠综合征一例. 中华医学杂志, 2017. 97(17): 第1352-1353页.

[68]. 刘史佳等, 基于PK-PD模型研究雷公藤治疗类风湿关节炎生物靶标. 中国中药杂志, 2015. 40(02): 第334-338页.

[69]. 刘史佳等, 基于PK-PD模型研究雷公藤治疗类风湿关节炎生物靶标. 中国中药杂志, 2015. 40(2): 第334-338页.

[70]. 雷旭杰, 基于骨代谢水平对断藤益母汤治疗中老年类风湿关节炎的疗效观察, 2018, 广州中医药大学.

[71]. 韩玉凤, 基于关节超声评价断藤益母汤联合来氟米特治疗类风湿关节炎的临床疗效, 2017, 广州中医药大学.

[72]. 汪元与刘健, 基于神经内分泌免疫网络学说探讨新风胶囊治疗类风湿关节炎的量效关系. 时珍国医国药, 2010. 21(10): 第2622-2624页.

[73]. 汪元与刘健, 基于神经内分泌免疫网络学说探讨新风胶囊治疗类风湿关节炎的量效关系. 时珍国医国药, 2010. 21(10): 第2622-2624页.

[74]. 孟庆良等, 基于文本挖掘技术析甲氨蝶呤与中医治疗联合应用的规律. 中国中医基础医学杂志, 2014. 20(12): 第1665-1667页.

[75]. 孟庆良等, 基于文本挖掘技术析甲氨蝶呤与中医治疗联合应用的规律. 中国中医基础医学杂志, 2014. 20(12): 第1665-1667页.

[76]. 杨锦屏, 杜烨辉与安静思. 激素及环磷酰胺冲击联合治疗类风湿关节炎合并肾脏损害18例分析. in 第十届全国风湿病学学术会议. 2005.

[77]. 布文才, 加减桂枝芍药知母汤治疗类风湿关节炎临床研究. 亚太传统医药, 2015. 11(06): 第135-136页.

[78]. 布文才, 加减桂枝芍药知母汤治疗类风湿关节炎临床研究. 亚太传统医药, 2015. 11(6): 第135-136页.

[79]. 苏秀芳与赵利平, 加味桂枝汤治疗产后类风湿性关节炎临床观察. 山西中医, 2013. 29(08): 第12-13+19页.

[80]. 苏秀芳与赵利平, 加味桂枝汤治疗产后类风湿性关节炎临床观察. 山西中医, 2013. 29(8): 第12-13,19页.

[81]. 倪斐, 甲氨喋呤联合雷公藤多甙片治疗类风湿性关节炎的效果. 心理月刊, 2018(2): 第270页.

[82]. 欧秋娟, 黄存军与廖湘平, 甲氨蝶呤和雷公藤多甙联合对类风湿关节炎的治疗效果评估. 内蒙古中医药, 2016. 35(17): 第50-51页.

[83]. 欧秋娟, 黄存军与廖湘平, 甲氨蝶呤和雷公藤多甙联合对类风湿关节炎的治疗效果评估. 内蒙古中医药, 2016. 35(17): 第50-51页.

[84]. 李烨, 甲氨蝶呤和雷公藤多甙联合治疗类风湿关节炎的临床观察. 现代医药卫生, 2007(05): 第639-640页.

[85]. 李烨, 甲氨蝶呤和雷公藤多甙联合治疗类风湿关节炎的临床观察. 现代医药卫生, 2007. 23(5): 第639-640页.

[86]. 朱春来与罗观, 甲氨蝶呤和硫酸软骨素联合雷公藤小剂量治疗类风湿关节炎临床疗效观察. 中国社区医师(医学专业), 2012. 14(12): 第135页.

[87]. 朱春来与罗观, 甲氨蝶呤和硫酸软骨素联合雷公藤小剂量治疗类风湿关节炎临床疗效观察. 中国社区医师（医学专业）, 2012. 14(12): 第135页.

[88]. 和雅等, 甲氨蝶呤加雷公藤多甙片对类风湿关节炎的效果对比研究. 泰山医学院学报, 2019. 40(10): 第775-776页.

[89]. 和雅等, 甲氨蝶呤加雷公藤多甙片对类风湿关节炎的效果对比研究. 泰山医学院学报, 2019. 40(10): 第775-776页.

[90]. 刘星与张华银, 甲氨蝶呤加雷公藤多甙片对类风湿关节炎的治疗作用研究. 当代医学, 2017. 23(29): 第71-72页.

[91]. 廖子鸿, 周志华与戴冠东, 甲氨蝶呤加用雷公藤多甙片治疗类风湿性关节炎的临床效果探析. 中国生化药物杂志, 2016. 36(05): 第136-138页.

[92]. 廖子鸿, 周志华与戴冠东, 甲氨蝶呤加用雷公藤多甙片治疗类风湿性关节炎的临床效果探析. 中国生化药物杂志, 2016(5): 第136-138页.

[93]. 莫美丽等, 甲氨蝶呤联合艾拉莫德治疗活动性类风湿关节炎的随机对照试验. 福建医科大学学报, 2018. 52(04): 第245-248页.

[94]. 莫美丽等, 甲氨蝶呤联合艾拉莫德治疗活动性类风湿关节炎的随机对照试验. 福建医科大学学报, 2018. 52(4): 第245-248页.

[95]. 孙玮与刘秀梅, 甲氨蝶呤联合来氟米特或雷公藤多苷治疗抗环瓜氨酸肽抗体阳性的早期类风湿关节炎的疗效比较. 山西医药杂志, 2010. 39(01): 第59-60页.

[96]. 孙玮与刘秀梅, 甲氨蝶呤联合来氟米特或雷公藤多苷治疗抗环瓜氨酸肽抗体阳性的早期类风湿关节炎的疗效比较. 山西医药杂志, 2010. 39(1): 第59-60页.

[97]. 范俊, 甲氨蝶呤联合雷公藤多甙片治疗类风湿性关节炎的疗效观察. 浙江临床医学, 2018. 20(5): 第834-835,838页.

[98]. 李爽, 甲氨蝶呤联合雷公藤多甙片治疗类风湿性关节炎的疗效及安全性评价. 中国疗养医学, 2016. 25(05): 第527-529页.

[99]. 李爽, 甲氨蝶呤联合雷公藤多甙片治疗类风湿性关节炎的疗效及安全性评价. 中国疗养医学, 2016. 25(5): 第527-529页.

[100]. 闫琳毅与陆利, 甲氨蝶呤联合雷公藤多甙片治疗类风湿性关节炎疗效及安全性研究. 中国现代医生, 2015. 53(08): 第90-92页.

[101]. 闫琳毅与陆利, 甲氨蝶呤联合雷公藤多甙片治疗类风湿性关节炎疗效及安全性研究. 中国现代医生, 2015. 53(8): 第90-92页.

[102]. 雷尚文等, 甲氨蝶呤联合雷公藤多苷对中老年类风湿关节炎患者的疗效及对金属蛋白酶-3的影响. 甘肃医药, 2020. 39(6): 第513-516页.

[103]. 雷尚文等, 甲氨蝶呤联合雷公藤多苷对中老年类风湿关节炎患者的治疗效果. 中国实用医药, 2020. 15(13): 第141-143页.

[104]. 雷尚文等, 甲氨蝶呤联合雷公藤多苷对中老年类风湿关节炎患者的治疗效果. 中国实用医药, 2020. 15(13): 第141-143页.

[105]. 朱琳等, 甲氨蝶呤联合雷公藤多苷片治疗老年类风湿关节炎的临床疗效及随访分析. 世界中西医结合杂志, 2020. 15(02): 第339-343+347页.

[106]. 朱琳等, 甲氨蝶呤联合雷公藤多苷片治疗老年类风湿关节炎的临床疗效及随访分析. 世界中西医结合杂志, 2020. 15(2): 第339-343,347页.

[107]. 李燕青, 郭春连与卢家淇, 甲氨蝶呤联合雷公藤多苷片治疗类风湿性关节炎患者的疗效及不良反应分析. 内科, 2019. 14(04): 第424-426页.

[108]. 李燕青, 郭春连与卢家淇, 甲氨蝶呤联合雷公藤多苷片治疗类风湿性关节炎患者的疗效及不良反应分析. 内科, 2019. 14(4): 第424-426页.

[109]. 朱琳与陈鹏, 甲氨蝶呤联合雷公藤多苷与单用甲氨蝶呤治疗类风湿关节炎效果比较. 检验医学与临床, 2015. 12(23): 第3568-3570页.

[110]. 朱琳与陈鹏, 甲氨蝶呤联合雷公藤多苷与单用甲氨蝶呤治疗类风湿关节炎效果比较. 检验医学与临床, 2015(23): 第3568-3570页.

[111]. 秦理, 杨孝兵与蒋峰. 甲氨蝶呤联合雷公藤多苷治疗绝经后类风湿关节炎. in 二零一四年浙江省风湿病学学术年会. 2014. 中国浙江湖州.

[112]. 杨敏等, 甲氨蝶呤联合雷公藤多苷治疗老年类风湿关节炎. 中国实验方剂学杂志, 2013. 19(17): 第300-304页.

[113]. 杨敏等, 甲氨蝶呤联合雷公藤多苷治疗老年类风湿关节炎. 中国实验方剂学杂志, 2013. 19(17): 第300-304页.

[114]. 刘海燕, 甲氨蝶呤联合雷公藤多苷治疗类风湿关节炎的临床效果观察. 临床合理用药杂志, 2019. 12(02): 第13-14页.

[115]. 刘海燕, 甲氨蝶呤联合雷公藤多苷治疗类风湿关节炎的临床效果观察. 临床合理用药杂志, 2019. 12(2): 第13-14页.

[116]. 王雪凤与栾照家, 甲氨蝶呤联合雷公藤多苷治疗类风湿性关节炎的临床疗效观察. 国际医药卫生导报, 2015. 21(17): 第2598-2600页.

[117]. 罗义根等, 甲氨蝶呤联合柳氮磺胺吡啶、雷公藤多甙片治疗类风湿关节炎疗效观察. 长江大学学报(自科版), 2014. 11(33): 第39-42页.

[118]. 罗义根等, 甲氨蝶呤联合柳氮磺胺吡啶、雷公藤多甙片治疗类风湿关节炎疗效观察. 长江大学学报（自科版）医学下旬刊, 2014(11): 第39-42页.

[119]. 朱芳晓, 周润华与石宇红, 甲氨蝶呤联合羟氯喹或雷公藤多苷治疗抗环瓜氨酸肽抗体阳性的早期类风湿关节炎的临床研究. 中国医药指南, 2012. 10(18): 第416-418页.

[120]. 朱芳晓, 周润华与石宇红, 甲氨蝶呤联合羟氯喹或雷公藤多苷治疗抗环瓜氨酸肽抗体阳性的早期类风湿关节炎的临床研究. 中国医药指南, 2012. 10(18): 第416-418页.

[121]. 李念羊, 吴宁宁与张培, 甲氨蝶呤联合曲安奈德治疗类风湿关节炎的效果分析. 中国当代医药, 2013. 20(23): 第85-86+88页.

[122]. 李念羊, 吴宁宁与张培, 甲氨蝶呤联合曲安奈德治疗类风湿关节炎的效果分析. 中国当代医药, 2013. 20(23): 第85-86,88页.

[123]. 高登文, 甲氨蝶呤联合小剂量来氟米特、雷公藤多甙片治疗类风湿性关节炎的疗效及安全性. 泰山医学院学报, 2017. 38(05): 第524-525页.

[124]. 高登文, 甲氨蝶呤联合小剂量来氟米特、雷公藤多甙片治疗类风湿性关节炎的疗效及安全性. 泰山医学院学报, 2017. 38(5): 第524-525页.

[125]. 卜晋安, 甲氨蝶呤联合中药治疗类风湿性关节炎22例临床疗效分析. 中国厂矿医学, 2008(04): 第432-433页.

[126]. 卜晋安, 甲氨蝶呤联合中药治疗类风湿性关节炎22例临床疗效分析. 中国厂矿医学, 2008. 21(4): 第432-433页.

[127]. 崔毅佳, 王淑梅与金志国, 甲氨蝶呤通过抑制TLR2-NF-κB信号通路减轻类风湿关节炎大鼠滑膜炎的作用研究. 新疆医科大学学报, 2019. 42(02): 第211-216页.

[128]. 崔毅佳, 王淑梅与金志国, 甲氨蝶呤通过抑制TLR2-NF-κB信号通路减轻类风湿关节炎大鼠滑膜炎的作用研究. 新疆医科大学学报, 2019. 42(2): 第211-216页.

[129]. 黄静等, 甲氨蝶呤与雷公藤多苷分别联合来氟米特治疗类风湿关节炎的效果比较. 药物评价研究, 2020. 43(01): 第103-106页.

[130]. 黄静等, 甲氨蝶呤与雷公藤多苷分别联合来氟米特治疗类风湿关节炎的效果比较. 药物评价研究, 2020. 43(1): 第103-106页.

[131]. 铁宁与张桂芝, 甲氨蝶呤与雷公藤多苷片联用对类风湿性关节炎大鼠的治疗作用. 中国中医急症, 2016. 25(04): 第655-657+674页.

[132]. 铁宁与张桂芝, 甲氨蝶呤与雷公藤多苷片联用对类风湿性关节炎大鼠的治疗作用. 中国中医急症, 2016. 25(4): 第655-657,674页.

[133]. 李思吟与邓代华, 甲氨蝶呤治疗类风湿性关节炎的应用及最佳剂量分析. 内江科技, 2018. 39(9): 第65-66,48页.

[134]. 刘健, 李华与谌曦, 健脾化湿通络法治疗类风湿关节炎贫血的临床研究. 中西医结合学报, 2006(04): 第348-354页.

[135]. 刘健, 李华与谌曦, 健脾化湿通络法治疗类风湿关节炎贫血的临床研究. 中西医结合学报, 2006. 4(4): 第348-354页.

[136]. 高照猛等, 抗苗勒管激素水平评价生育期女性类风湿关节炎患者卵巢储备功能的临床意义. 中国现代医生, 2018. 56(14): 第21-26页.

[137]. 高照猛等, 抗苗勒管激素水平评价生育期女性类风湿关节炎患者卵巢储备功能的临床意义. 中国现代医生, 2018. 56(14): 第21-26页.

[138]. 王启芬, 昆明山海棠根茎不同投药部位对二藤通痹合剂抗炎药效的比较研究, 2015, 广州中医药大学.

[139]. 范仰钢与李国华, 昆明山海棠联合甲氨蝶呤治疗老年起病类风湿关节炎. 现代医药卫生, 2006(04): 第478-480页.

[140]. 范仰钢与李国华, 昆明山海棠联合甲氨蝶呤治疗老年起病类风湿关节炎. 现代医药卫生, 2006. 22(4): 第478-480页.

[141]. 唐志宇与梁江, 昆山合剂对类风湿关节炎合并贫血患者滑膜成纤维细胞增殖及MyD88、IL-6表达的研究. 世界科学技术-中医药现代化, 2014. 16(03): 第582-586页.

[142]. 唐志宇与梁江, 昆山合剂对类风湿关节炎合并贫血患者滑膜成纤维细胞增殖及MyD88、IL-6表达的研究. 世界科学技术-中医药现代化, 2014(3): 第582-586页.

[143]. 林昌松等, 昆仙胶囊联合甲氨蝶呤治疗类风湿关节炎疗效观察. 陕西中医, 2010. 31(08): 第987-990页.

[144]. 林昌松等, 昆仙胶囊联合甲氨蝶呤治疗类风湿关节炎疗效观察. 陕西中医, 2010. 31(8): 第987-990页.

[145]. 王笑丹, 昆仙胶囊治疗类风湿关节炎临床疗效评价及对CIA大鼠IL-8、γIP-10影响, 2011, 广州中医药大学.

[146]. 朱建琴与钱建东, 来氟米特联合甲氨蝶呤治疗类风湿关节炎的疗效观察. 医药前沿, 2016(4): 第192-193页.

[147]. 卢红平, 来氟米特联合甲氨蝶呤治疗难治性类风湿关节炎疗效观察. 现代中西医结合杂志, 2013. 22(18): 第1979-1980页.

[148]. 卢红平, 来氟米特联合甲氨蝶呤治疗难治性类风湿关节炎疗效观察. 现代中西医结合杂志, 2013. 22(18): 第1979-1980页.

[149]. 邓媛等, 雷公藤对类风湿关节炎疗效及IgA、IgG、RF变化研究. 中华中医药学刊, 2020. 38(02): 第234-236页.

[150]. 邓媛等, 雷公藤对类风湿关节炎疗效及IgA、IgG、RF变化研究. 中华中医药学刊, 2020. 38(2): 第234-236页.

[151]. 沈杰与张之澧, 雷公藤多甙合小剂量甲氨蝶呤治疗老年性类风湿性关节炎. 浙江中西医结合杂志, 2002(06): 第7-9页.

[152]. 沈杰与张之澧, 雷公藤多甙合小剂量甲氨蝶呤治疗老年性类风湿性关节炎. 浙江中西医结合杂志, 2002. 12(6): 第334-336页.

[153]. 赵玲, 雷公藤多甙联合丹参酮ⅡA对胶原诱导关节炎大鼠心血管损伤的保护作用及机制探讨, 2008, 河北医科大学.

[154]. 冯江江, 雷公藤多甙联合甲氨蝶呤治疗类风湿关节炎的疗效分析. 中西医结合心血管病电子杂志, 2019. 7(25): 第194,196页.

[155]. 李广科, 雷公藤多甙联合甲氨蝶呤治疗类风湿关节炎的疗效研究. 齐齐哈尔医学院学报, 2017. 38(17): 第2007-2008页.

[156]. 李广科, 雷公藤多甙联合甲氨蝶呤治疗类风湿关节炎的疗效研究. 齐齐哈尔医学院学报, 2017. 38(17): 第2007-2008页.

[157]. 周飞等, 雷公藤多甙联合甲氨蝶呤治疗类风湿关节炎的临床疗效及机制探讨. 中南医学科学杂志, 2018. 46(03): 第257-259+266页.

[158]. 周飞等, 雷公藤多甙联合甲氨蝶呤治疗类风湿关节炎的临床疗效及机制探讨. 中南医学科学杂志, 2018. 46(3): 第257-259,266页.

[159]. 杨春燕, 李梦霞与徐笑笑, 雷公藤多甙联合甲氨蝶呤治疗类风湿关节炎患者的临床效果观察. 临床合理用药杂志, 2019. 12(11): 第69-70页.

[160]. 杨春燕, 李梦霞与徐笑笑, 雷公藤多甙联合甲氨蝶呤治疗类风湿关节炎患者的临床效果观察. 临床合理用药杂志, 2019. 12(11): 第69-70页.

[161]. 谭晴心与肖琴, 雷公藤多甙联合甲氨蝶呤治疗类风湿关节炎疗效评价及对TNF-α、IL-6的影响. 中国中医药信息杂志, 2010. 17(09): 第7-9页.

[162]. 谭晴心与肖琴, 雷公藤多甙联合甲氨蝶呤治疗类风湿关节炎疗效评价及对TNF-α、IL-6的影响. 中国中医药信息杂志, 2010. 17(9): 第7-9页.

[163]. 陆栋, 雷公藤多甙联合甲氨蝶呤治疗类风湿性关节炎的疗效及安全性观察. 中国医学工程, 2016. 24(11): 第96-97页.

[164]. 陆栋, 雷公藤多甙联合甲氨蝶呤治疗类风湿性关节炎的疗效及安全性观察. 中国医学工程, 2016. 24(11): 第96-97页.

[165]. 赵法来与刘士同, 雷公藤多甙联合甲氨蝶呤治疗类风湿性关节炎临床疗效观察. 首都食品与医药, 2020. 27(07): 第83页.

[166]. 赵法来与刘士同, 雷公藤多甙联合甲氨蝶呤治疗类风湿性关节炎临床疗效观察. 首都食品与医药, 2020. 27(7): 第83页.

[167]. 张小翠, 雷公藤多甙联合来氟米特及甲氨蝶呤治疗类风湿关节炎疗效观察. 山西中医, 2017. 33(09): 第28-29+34页.

[168]. 张小翠, 雷公藤多甙联合来氟米特及甲氨蝶呤治疗类风湿关节炎疗效观察. 山西中医, 2017. 33(9): 第28-29,34页.

[169]. 吴敏与马英淳. 雷公藤多甙联合柳氮磺吡啶治疗老年类风湿关节炎的临床观察. in 第十届全国风湿病学学术会议. 2005.

[170]. 李凌汉, 麦培根与陈宝红, 雷公藤多甙联合免疫抑制剂治疗类风湿关节炎疗效及对炎性因子的影响. 现代中西医结合杂志, 2017. 26(10): 第1088-1090页.

[171]. 李凌汉, 麦培根与陈宝红, 雷公藤多甙联合免疫抑制剂治疗类风湿关节炎疗效及对炎性因子的影响. 现代中西医结合杂志, 2017. 26(10): 第1088-1090页.

[172]. 于得泓, 战艺与石红, 雷公藤多甙片联合甲氨喋呤治疗对类风湿关节炎患者红细胞沉降率、C反应蛋白及类风湿因子的影响. 中国医药科学, 2019. 9(16): 第90-92+157页.

[173]. 于得泓, 战艺与石红, 雷公藤多甙片联合甲氨喋呤治疗对类风湿关节炎患者红细胞沉降率、C反应蛋白及类风湿因子的影响. 中国医药科学, 2019. 9(16): 第90-92,157页.

[174]. 庄铭城, 王小燕与陈培嘉, 雷公藤多甙片联合甲氨喋呤治疗类风湿关节炎的临床疗效及对炎症因子的影响分析. 中国医药科学, 2018. 8(18): 第46-49页.

[175]. 庄铭城, 王小燕与陈培嘉, 雷公藤多甙片联合甲氨喋呤治疗类风湿关节炎的临床疗效及对炎症因子的影响分析. 中国医药科学, 2018. 8(18): 第46-49页.

[176]. 徐光平, 徐海燕与于久秀, 雷公藤多甙片联合甲氨蝶呤治疗31例类风湿性关节炎患者的临床疗效观察. 中国农村卫生, 2017(18): 第93-94页.

[177]. 徐光平, 徐海燕与于久秀, 雷公藤多甙片联合甲氨蝶呤治疗31例类风湿性关节炎患者的临床疗效观察. 中国农村卫生, 2017(18): 第93-94页.

[178]. 侯宏理与马琳琳, 雷公藤多甙片联合甲氨蝶呤治疗活动性类风湿性关节炎的临床研究. 内蒙古中医药, 2017. 36(17): 第43页.

[179]. 王淼, 雷公藤多甙片联合甲氨蝶呤治疗类风湿性关节炎临床研究. 医药论坛杂志, 2016. 37(07): 第144-145页.

[180]. 王淼, 雷公藤多甙片联合甲氨蝶呤治疗类风湿性关节炎临床研究. 医药论坛杂志, 2016. 37(7): 第144-145页.

[181]. 陈芍等, 雷公藤多甙片与甲氨喋呤联合治疗类风湿关节炎的临床疗效及对炎症因子的影响. 现代生物医学进展, 2017. 17(04): 第713-716页.

[182]. 陈芍等, 雷公藤多甙片与甲氨喋呤联合治疗类风湿关节炎的临床疗效及对炎症因子的影响. 现代生物医学进展, 2017. 17(4): 第713-716页.

[183]. 王晓月等, 雷公藤多苷(甙)片单用或联合甲氨蝶呤对类风湿关节炎临床表现改善作用的Meta分析. 中国中药杂志, 2019. 44(16): 第3533-3541页.

[184]. 王晓月等, 雷公藤多苷(甙)片单用或联合甲氨蝶呤对类风湿关节炎临床表现改善作用的Meta分析. 中国中药杂志, 2019. 44(16): 第3533-3541页.

[185]. 李泰贤等, 雷公藤多苷(甙)片单用或联合甲氨蝶呤治疗类风湿关节炎实验室指标改善作用的Meta分析. 中国中药杂志, 2019. 44(16): 第3542-3550页.

[186]. 李泰贤等, 雷公藤多苷(甙)片单用或联合甲氨蝶呤治疗类风湿关节炎实验室指标改善作用的Meta分析. 中国中药杂志, 2019. 44(16): 第3542-3550页.

[187]. 陈文佳等, 雷公藤多苷(甙)片单用或联用甲氨蝶呤治疗类风湿关节炎临床疗效RCT研究的Meta分析. 中国中药杂志, 2020. 45(4): 第791-797页.

[188]. 杨俊等, 雷公藤多苷(甙)片对类风湿关节炎促炎细胞因子影响的系统评价. 中国中药杂志, 2020. 45(4): 第764-774页.

[189]. 李逸群等, 雷公藤多苷(甙)片治疗类风湿关节炎的安全性系统评价. 中国中药杂志, 2020. 45(4): 第775-790页.

[190]. 谢春花, 雷公藤多苷+甲氨蝶呤治疗类风湿性关节炎的效果及不良反应发生率影响评价. 临床医药文献电子杂志, 2019. 6(80): 第164,171页.

[191]. 王志强等, 雷公藤多苷不同时间给药联合甲氨蝶呤治疗难治性类风湿关节炎疗效观察. 现代中西医结合杂志, 2020. 29(01): 第11-14+54页.

[192]. 王志强等, 雷公藤多苷不同时间给药联合甲氨蝶呤治疗难治性类风湿关节炎疗效观察. 现代中西医结合杂志, 2020. 29(1): 第11-14,54页.

[193]. 杨宏伟, 涂胜豪与常栋, 雷公藤多苷对胶原诱导性关节炎大鼠中高迁移率族蛋白B1影响的研究. 中华风湿病学杂志, 2011(08): 第550-552+585页.

[194]. 杨宏伟, 涂胜豪与常栋, 雷公藤多苷对胶原诱导性关节炎大鼠中高迁移率族蛋白B1影响的研究. 中华风湿病学杂志, 2011. 15(8): 第550-552,后插1页.

[195]. 刘巍与张艳艳, 雷公藤多苷对类风湿关节炎患者成纤维样滑膜细胞α7nAChR及炎症因子的作用. 山东中医杂志, 2019. 38(12): 第1166-1170+1197页.

[196]. 刘巍与张艳艳, 雷公藤多苷对类风湿关节炎患者成纤维样滑膜细胞α7nAChR及炎症因子的作用. 山东中医杂志, 2019. 38(12): 第1166-1170,1197页.

[197]. 孙凤艳等, 雷公藤多苷对类风湿关节炎患者滤泡辅助性T细胞及IL-21的影响. 医学综述, 2016. 22(03): 第566-569页.

[198]. 罗波等, 雷公藤多苷对佐剂性关节炎模型大鼠关节中核因子κB受体激活剂配基表达的影响. 医药导报, 2006(05): 第395-397页.

[199]. 罗波等, 雷公藤多苷对佐剂性关节炎模型大鼠关节中核因子κB受体激活剂配基表达的影响. 医药导报, 2006. 25(5): 第395-397页.

[200]. 李媛, 雷公藤多苷和甲氨蝶呤联合治疗类风湿性关节炎的临床研究. 中国处方药, 2019. 17(11): 第107-109页.

[201]. 李英, 雷公藤多苷联合氨甲蝶呤与来氟米特治疗类风湿关节炎患者的效果. 中国民康医学, 2020. 32(10): 第85-86页.

[202]. 李英, 雷公藤多苷联合氨甲蝶呤与来氟米特治疗类风湿关节炎患者的效果. 中国民康医学, 2020. 32(10): 第85-86页.

[203]. 胡旭君与宋欣伟, 雷公藤多苷联合甲氨蝶呤对干燥综合征NOD小鼠治疗作用及TNF-α、IL-1β、AQP-5的表达. 中华中医药杂志, 2014. 29(07): 第2362-2366页.

[204]. 胡旭君与宋欣伟, 雷公藤多苷联合甲氨蝶呤对干燥综合征NOD小鼠治疗作用及TNF-α、IL-1β、AQP-5的表达. 中华中医药杂志, 2014. 29(7): 第2362-2366页.

[205]. 刘翠莲与蔡文虹, 雷公藤多苷联合甲氨蝶呤对老年类风湿关节炎患者血清中细胞因子水平的影响. 中国处方药, 2019. 17(06): 第93-94页.

[206]. 刘翠莲与蔡文虹, 雷公藤多苷联合甲氨蝶呤对老年类风湿关节炎患者血清中细胞因子水平的影响. 中国处方药, 2019. 17(6): 第93-94页.

[207]. 冯艳广与王晓寒, 雷公藤多苷联合甲氨蝶呤对类风湿关节炎患者TfH细胞及IL-21的影响. 慢性病学杂志, 2017. 18(11): 第1296-1298页.

[208]. 冯艳广与王晓寒, 雷公藤多苷联合甲氨蝶呤对类风湿关节炎患者TfH细胞及IL-21的影响. 慢性病学杂志, 2017(011).

[209]. 胡文娟, 雷公藤多苷联合甲氨蝶呤对类风湿性关节炎患者血清CRP ESR RF水平的影响. 基层医学论坛, 2020. 24(32): 第4647-4648页.

[210]. 高利霞, 雷公藤多苷联合甲氨蝶呤片治疗类风湿关节炎肺间质病变效果观察. 白求恩医学杂志, 2020. 18(02): 第143-145页.

[211]. 高利霞, 雷公藤多苷联合甲氨蝶呤片治疗类风湿关节炎肺间质病变效果观察. 白求恩医学杂志, 2020. 18(2): 第143-145页.

[212]. 陈明雁, 雷公藤多苷联合甲氨蝶呤治疗RA效果更好. 中华医学信息导报, 2014. 29(08): 第7页.

[213]. 尹聪等, 雷公藤多苷联合甲氨蝶呤治疗类风湿关节炎的Meta分析. 中国组织工程研究, 2019. 23(35): 第5710-5717页.

[214]. 尹聪等, 雷公藤多苷联合甲氨蝶呤治疗类风湿关节炎的Meta分析. 中国组织工程研究, 2019. 23(35): 第5710-5717页.

[215]. 张跃军, 雷公藤多苷联合甲氨蝶呤治疗类风湿关节炎的临床分析. 中国卫生标准管理, 2020. 11(12): 第101-103页.

[216]. 刘君勇与罗莉容, 雷公藤多苷联合甲氨蝶呤治疗类风湿关节炎的临床价值研究. 中国处方药, 2019. 17(03): 第67-68页.

[217]. 刘君勇与罗莉容, 雷公藤多苷联合甲氨蝶呤治疗类风湿关节炎的临床价值研究. 中国处方药, 2019. 17(3): 第67-68页.

[218]. 毕丹艳等, 雷公藤多苷联合甲氨蝶呤治疗类风湿关节炎的临床疗效研究. 中国临床药理学杂志, 2016. 32(10): 第880-882页.

[219]. 毕丹艳等, 雷公藤多苷联合甲氨蝶呤治疗类风湿关节炎的临床疗效研究. 中国临床药理学杂志, 2016. 32(10): 第880-882页.

[220]. 陈鹏等, 雷公藤多苷联合甲氨蝶呤治疗类风湿关节炎的随机对照试验. 安徽中医学院学报, 2011. 30(06): 第28-32页.

[221]. 陈鹏等, 雷公藤多苷联合甲氨蝶呤治疗类风湿关节炎的随机对照试验. 安徽中医学院学报, 2011. 30(6): 第28-32页.

[222]. 张卫华等, 雷公藤多苷联合甲氨蝶呤治疗类风湿关节炎的效果分析. 临床医学, 2019. 39(06): 第92-93页.

[223]. 张卫华等, 雷公藤多苷联合甲氨蝶呤治疗类风湿关节炎的效果分析. 临床医学, 2019. 39(6): 第92-93页.

[224]. 潘祝平, 林顺平与林旋, 雷公藤多苷联合甲氨蝶呤治疗类风湿关节炎短期疗效观察. 风湿病与关节炎, 2014. 3(03): 第17-20页.

[225]. 潘祝平, 林顺平与林旋, 雷公藤多苷联合甲氨蝶呤治疗类风湿关节炎短期疗效观察. 风湿病与关节炎, 2014(3): 第17-20页.

[226]. 王在红, 杨金玲与王露, 雷公藤多苷联合甲氨蝶呤治疗类风湿关节炎患者的效果. 中国民康医学, 2020. 32(20): 第74-76页.

[227]. 陈曾凤等, 雷公藤多苷联合甲氨蝶呤治疗类风湿关节炎活动期患者的疗效及对血清CD62p、CD41的影响. 现代生物医学进展, 2018. 18(20): 第3909-3912+3921页.

[228]. 陈曾凤等, 雷公藤多苷联合甲氨蝶呤治疗类风湿关节炎活动期患者的疗效及对血清CD62p、CD41的影响. 现代生物医学进展, 2018. 18(20): 第3909-3912,3921页.

[229]. 林顺平. 雷公藤多苷联合甲氨蝶呤治疗类风湿关节炎疗效评价. in 第17次全国风湿病学学术会议. 2012. 中国云南昆明.

[230]. 林顺平. 雷公藤多苷联合甲氨蝶呤治疗类风湿关节炎疗效评价. in 第17次全国风湿病学学术会议. 2012. 昆明.

[231]. 袁毅, 雷公藤多苷联合甲氨蝶呤治疗类风湿关节炎效果观察. 临床医学, 2018. 38(01): 第100-101页.

[232]. 袁毅, 雷公藤多苷联合甲氨蝶呤治疗类风湿关节炎效果观察. 临床医学, 2018. 38(1): 第100-101页.

[233]. 高鹏, 霍爱鑫与刘宇宏, 雷公藤多苷联合甲氨蝶呤治疗类风湿性关节炎的疗效. 西部医学, 2017. 29(11): 第1511-1515页.

[234]. 高鹏, 霍爱鑫与刘宇宏, 雷公藤多苷联合甲氨蝶呤治疗类风湿性关节炎的疗效. 西部医学, 2017. 29(11): 第1511-1515页.

[235]. 王露, 雷公藤多苷联合甲氨蝶呤治疗类风湿性关节炎的临床疗效. 中国实用医刊, 2018. 45(10): 第117-119页.

[236]. 王露, 雷公藤多苷联合甲氨蝶呤治疗类风湿性关节炎的临床疗效. 中国实用医刊, 2018. 45(10): 第117-119页.

[237]. 张文娟, 雷公藤多苷联合甲氨蝶呤治疗类风湿性关节炎短期疗效及对患者外周血炎性因子的影响. 世界最新医学信息文摘, 2019. 19(51): 第129-130页.

[238]. 张文娟, 雷公藤多苷联合甲氨蝶呤治疗类风湿性关节炎短期疗效及对患者外周血炎性因子的影响. 世界最新医学信息文摘（连续型电子期刊）, 2019. 19(51): 第129-130页.

[239]. 张红与薛中柱, 雷公藤多苷联合甲氨蝶呤治疗类风湿性关节炎短期疗效研究. 吉林中医药, 2018. 38(06): 第660-663页.

[240]. 赵旭颖等, 雷公藤多苷联合硫酸羟氯喹治疗类风湿关节炎致急性粒细胞缺乏后的治疗探讨. 中国医刊, 2020. 55(10): 第1135-1138页.

[241]. 陈继红, 劳志英与何东仪, 雷公藤多苷联合青藤碱治疗类风湿关节炎49例. 上海中医药杂志, 2011. 45(10): 第64-65页.

[242]. 陈继红, 劳志英与何东仪, 雷公藤多苷联合青藤碱治疗类风湿关节炎49例. 上海中医药杂志, 2011. 45(10): 第64-65页.

[243]. 辛立波, 雷公藤多苷联合人参皂苷对巨噬细胞移动抑制因子诱导大鼠成纤维样滑膜细胞增殖及RANKL/OPG表达的影响, 2011, 河北医科大学.

[244]. 马华, 雷公藤多苷联合人参皂苷对佐剂性关节炎大鼠的骨保护作用及机制探讨, 2010, 河北医科大学.

[245]. 马华等, 雷公藤多苷联合双醋瑞因对类风湿关节炎中IL-1、TNF-α、OPG、RANKL的影响. 中国继续医学教育, 2016. 8(36): 第137-139页.

[246]. 马华等, 雷公藤多苷联合双醋瑞因对类风湿关节炎中IL-1、TNF-α、OPG、RANKL的影响. 中国继续医学教育, 2016. 8(36): 第137-139页.

[247]. 沈杰与张之澧. 雷公藤多苷联合小剂量甲氨蝶呤治疗老年性类风湿关节炎临床观察. in 第四次全国雷公藤学术会议. 2004. 上海.

[248]. 莫菁莲, 王政与张瑞城, 雷公藤多苷联合依那西普治疗老年类风湿关节炎及其对血清炎性因子和骨代谢因子的影响. 中国老年学杂志, 2020. 40(13): 第2810-2812页.

[249]. 莫菁莲, 王政与张瑞城, 雷公藤多苷联合依那西普治疗老年类风湿关节炎及其对血清炎性因子和骨代谢因子的影响. 中国老年学杂志, 2020. 40(13): 第2810-2812页.

[250]. 王丽华, 雷公藤多苷配伍甲氨蝶呤及来氟米特治疗类风湿关节炎的临床研究. 世界最新医学信息文摘, 2019. 19(62): 第209+211页.

[251]. 王丽华, 雷公藤多苷配伍甲氨蝶呤及来氟米特治疗类风湿关节炎的临床研究. 世界最新医学信息文摘（连续型电子期刊）, 2019. 19(62): 第209,211页.

[252]. 张婉, 雷公藤多苷配伍甲氨蝶呤及来氟米特治疗类风湿性关节炎的临床效果分析. 国际免疫学杂志, 2018. 41(02): 第243-246页.

[253]. 张婉, 雷公藤多苷配伍甲氨蝶呤及来氟米特治疗类风湿性关节炎的临床效果分析. 国际免疫学杂志, 2018. 41(2): 第243-246页.

[254]. 陈伏宇, 于广莹与王慧, 雷公藤多苷片、新癀片合用治疗湿热瘀阻型类风湿关节炎80例. 中医临床研究, 2010. 02(22): 第86-87页.

[255]. 谷敬欣等, 雷公藤多苷片联合甲氨蝶呤对类风湿关节炎合并骨质疏松患者血清骨代谢标志物水平及炎症因子的影响. 现代中西医结合杂志, 2020. 29(22): 第2424-2428页.

[256]. 谷敬欣等, 雷公藤多苷片联合甲氨蝶呤对类风湿关节炎合并骨质疏松患者血清骨代谢标志物水平及炎症因子的影响. 现代中西医结合杂志, 2020. 29(22): 第2424-2428页.

[257]. 王海波, 崔永虹与刘杰, 雷公藤多苷片联合甲氨蝶呤片治疗类风湿性关节炎临床分析. 临床合理用药杂志, 2016. 9(35): 第52-53页.

[258]. 王海波, 崔永虹与刘杰, 雷公藤多苷片联合甲氨蝶呤片治疗类风湿性关节炎临床分析. 临床合理用药杂志, 2016. 9(35): 第52-53页.

[259]. 郇稳等, 雷公藤多苷片联合甲氨蝶呤治疗类风湿关节炎对患者骨代谢及血清炎性因子水平的影响. 中国实用医刊, 2020. 47(3): 第90-93页.

[260]. 王慧娟, 雷公藤多苷片联合甲氨蝶呤治疗类风湿性关节炎的临床价值分析. 四川解剖学杂志, 2018. 26(04): 第92-93页.

[261]. 王慧娟, 雷公藤多苷片联合甲氨蝶呤治疗类风湿性关节炎的临床价值分析. 四川解剖学杂志, 2018. 26(4): 第92-93页.

[262]. 龙洁等, 雷公藤多苷片联合甲氨蝶呤治疗类风湿性关节炎的效果. 中国医药导报, 2019. 16(07): 第71-75页.

[263]. 龙洁等, 雷公藤多苷片联合甲氨蝶呤治疗类风湿性关节炎的效果. 中国医药导报, 2019. 16(7): 第71-75页.

[264]. 杜金万, 杜川与郑利强, 雷公藤多苷片治疗类风湿关节炎对患者免疫蛋白的影响. 临床合理用药杂志, 2019. 12(13): 第35-37页.

[265]. 杜金万, 杜川与郑利强, 雷公藤多苷片治疗类风湿关节炎对患者免疫蛋白的影响. 临床合理用药杂志, 2019. 12(13): 第35-37页.

[266]. 朱光昭等, 雷公藤多苷片治疗类风湿关节炎骨破坏的系统评价和Meta分析. 中国中药杂志, 2019. 44(15): 第3358-3364页.

[267]. 朱光昭等, 雷公藤多苷片治疗类风湿关节炎骨破坏的系统评价和Meta分析. 中国中药杂志, 2019. 44(15): 第3358-3364页.

[268]. 龙朝阳等, 雷公藤多苷片治疗类风湿关节炎老年患者的临床疗效. 内蒙古中医药, 2019. 38(9): 第72-73页.

[269]. 周红, 雷公藤多苷与甲氨蝶呤联合治疗类风湿关节炎的疗效分析. 中国医药指南, 2017. 15(06): 第191页.

[270]. 周红, 雷公藤多苷与甲氨蝶呤联合治疗类风湿关节炎的疗效分析. 中国医药指南, 2017. 15(6): 第191页.

[271]. 郑冰, 雷公藤多苷与甲氨蝶呤联合治疗类风湿关节炎临床疗效和安全性分析. 医药前沿, 2017. 7(15): 第360-361页.

[272]. 李松伟等, 雷公藤多苷治疗类风湿关节炎肺间质病变的临床研究. 中华中医药学刊, 2017. 35(07): 第1662-1664页.

[273]. 李松伟等, 雷公藤多苷治疗类风湿关节炎肺间质病变的临床研究. 中华中医药学刊, 2017. 35(7): 第1662-1664页.

[274]. 刘敏等, 雷公藤多苷治疗类风湿性关节炎的临床观察及对血清VEGF、VEGFR2表达水平的影响研究. 陕西中医, 2016. 37(01): 第72-74页.

[275]. 刘敏等, 雷公藤多苷治疗类风湿性关节炎的临床观察及对血清VEGF、VEGFR2表达水平的影响研究. 陕西中医, 2016. 37(1): 第72-74页.

[276]. 宋绍亮与马宏洋, 雷公藤复方预防类风湿关节炎患者依那西普诱导缓解后复发效果观察. 山东医药, 2014. 54(36): 第97-98页.

[277]. 宋绍亮与马宏洋, 雷公藤复方预防类风湿关节炎患者依那西普诱导缓解后复发效果观察. 山东医药, 2014(36): 第97-98页.

[278]. 赵钟文等. 雷公藤合剂配合手关节操治疗活动期RA临床研究. in 全国第十二届中西医结合风湿病学术会议. 2014. 天津.

[279]. 赵钟文等, 雷公藤合剂配合手关节操治疗活动期类风湿关节炎临床研究. 中医药通报, 2014. 13(4): 第40-44页.

[280]. 罗波等, 雷公藤甲素对佐剂性关节炎大鼠外周血单个核细胞核因子κB受体激活剂配基表达的影响. 华中科技大学学报(医学版), 2006(02): 第265-267页.

[281]. 罗波等, 雷公藤甲素对佐剂性关节炎大鼠外周血单个核细胞核因子κB受体激活剂配基表达的影响. 华中科技大学学报（医学版）, 2006. 35(2): 第265-267页.

[282]. 范文强等, 雷公藤甲素治疗类风湿关节炎的作用机制及安全性分析. 中草药, 2019. 50(16): 第3866-3871页.

[283]. 范文强等, 雷公藤甲素治疗类风湿关节炎的作用机制及安全性分析. 中草药, 2019. 50(16): 第3866-3871页.

[284]. 王永强, 雷公藤联合甲氨蝶呤治疗类风湿关节炎的疗效观察. 中国基层医药, 2013. 20(11): 第1678-1680页.

[285]. 王永强, 雷公藤联合甲氨蝶呤治疗类风湿关节炎的疗效观察. 中国基层医药, 2013. 20(11): 第1678-1680页.

[286]. 王萍, 雷公藤联合甲氨蝶呤治疗类风湿性关节炎的效果比较. 中国医药指南, 2019. 17(05): 第2-3页.

[287]. 王萍, 雷公藤联合甲氨蝶呤治疗类风湿性关节炎的效果比较. 中国医药指南, 2019. 17(5): 第2-3页.

[288]. 马俊福等, 雷公藤内酯醇对胶原诱导性关节炎大鼠γ-干扰素和白细胞介素-17A表达的影响. 北京中医药大学学报, 2020. 43(7): 第592-598页.

[289]. 王玉等, 雷公藤内酯醇对佐剂关节炎模型大鼠关节中核因子-κB受体激活剂配基表达的影响. 安徽中医学院学报, 2007(03): 第28-30页.

[290]. 王玉等, 雷公藤内酯醇对佐剂关节炎模型大鼠关节中核因子-κB受体激活剂配基表达的影响. 安徽中医学院学报, 2007. 26(3): 第28-30页.

[291]. 李萍, 雷公藤炮制后在临床中的应用. 中国医药指南, 2019. 17(04): 第160页.

[292]. 李萍, 雷公藤炮制后在临床中的应用. 中国医药指南, 2019. 17(4): 第160页.

[293]. 于守杰与贾倩, 雷公藤片联合甲氨蝶呤对类风湿关节炎的疗效观察. 基层医学论坛, 2020. 24(22): 第3155-3157页.

[294]. 于守杰与贾倩, 雷公藤片联合甲氨蝶呤对类风湿关节炎的疗效观察. 基层医学论坛, 2020. 24(22): 第3155-3157页.

[295]. 何小宇等, 雷公藤片治疗类风湿关节炎的疗效分析. 慢性病学杂志, 2017. 18(05): 第575-576页.

[296]. 杨竹, 雷公藤片治疗类风湿性关节炎74例. 中国药业, 2011. 20(14): 第76-77页.

[297]. 杨竹, 雷公藤片治疗类风湿性关节炎74例. 中国药业, 2011. 20(14): 第76-77页.

[298]. 余效福与余培红, 雷公藤药酒治疗类风湿性关节炎148例临床研究. 医学信息, 2015(27): 第317-317页.

[299]. 余效福, 雷公藤药酒治疗难治性类风湿性关节炎48例临床研究. 宁夏医学杂志, 2020. 42(03): 第281-283页.

[300]. 余效福, 雷公藤药酒治疗难治性类风湿性关节炎48例临床研究. 宁夏医学杂志, 2020. 42(3): 第281-283页.

[301]. 王妍, 雷公藤与甲氨蝶呤、黄芪与当归联用治疗RA机制的生物信息学分析, 2012, 中国中医科学院.

[302]. 杨荣, 张姝与王永福, 雷公藤在常见结缔组织病中的应用研究进展. 包头医学院学报, 2015(8): 第154-156页.

[303]. 李芯, 雷公藤治疗类风湿关节炎的疗效及安全性评估, 2012, 清华大学医学部;北京协和医学院;中国医学科学院.

[304]. 王娟芳, 雷公藤治疗类风湿性关节炎的疗效观察. 内蒙古中医药, 2017. 36(04): 第31页.

[305]. 王娟芳, 雷公藤治疗类风湿性关节炎的疗效观察. 内蒙古中医药, 2017. 36(4): 第31页.

[306]. 吴厅与宋欣伟, 类风湿Ⅰ号联合西药治疗湿热阻络型类风湿关节炎临床研究. 新中医, 2019. 51(07): 第163-166页.

[307]. 吴厅与宋欣伟, 类风湿Ⅰ号联合西药治疗湿热阻络型类风湿关节炎临床研究. 新中医, 2019. 51(7): 第163-166页.

[308]. 任璇璇等, 类风湿Ⅰ号丸增加高度活动类风湿关节炎脾气亏虚、湿热阻络证疗效的临床观察. 中华中医药学刊, 2014. 32(03): 第578-581页.

[309]. 任璇璇等, 类风湿Ⅰ号丸增加高度活动类风湿关节炎脾气亏虚、湿热阻络证疗效的临床观察. 中华中医药学刊, 2014. 32(3): 第578-581页.

[310]. 闫磊, 戚务芳与李媛, 类风湿关节炎伴血管炎合并神经损害1例. 中国免疫学杂志, 2017. 33(12): 第1874-1876页.

[311]. 闫磊, 戚务芳与李媛, 类风湿关节炎伴血管炎合并神经损害1例. 中国免疫学杂志, 2017. 33(12): 第1874-1876页.

[312]. 姜林娣, 梅振武与倪立清, 类风湿关节炎二线药物用药决策分析. 中国药物与临床, 2001(01): 第20-24页.

[313]. 姜林娣, 梅振武与倪立清, 类风湿关节炎二线药物用药决策分析. 中国药物与临床, 2001. 1(1): 第20-24页.

[314]. 郭颖, 逄崇杰与巩路, 类风湿关节炎合并重症肌无力一例. 中华风湿病学杂志, 2012. 16(10): 第718页.

[315]. 余步云等, 类风湿关节炎疗效的追踪观察. 中国药物与临床, 2001(01): 第13-15页.

[316]. 余步云等, 类风湿关节炎疗效的追踪观察. 中国药物与临床, 2001. 1(1): 第13-15页.

[317]. 陈锦然, 类风湿性关节炎采用雷公藤多苷与甲氨蝶呤联合治疗的效果. 实用中西医结合临床, 2019. 19(7): 第145-146页.

[318]. 陈小庄与吴苗青, 类风湿性关节炎联合药物治疗体会. 中国热带医学, 2006(03): 第472-473页.

[319]. 陈小庄与吴苗青, 类风湿性关节炎联合药物治疗体会. 中国热带医学, 2006. 6(3): 第472-473页.

[320]. 许海艳等, 类风湿性关节炎治疗方法的研究进展. 南昌大学学报（医学版）, 2020. 60(5): 第97-102页.

[321]. 许丹, 类叶牡丹有效部位干预胶原诱导型关节炎大鼠的代谢组学研究, 2018, 黑龙江中医药大学.

[322]. 徐卫东等, 利用文本挖掘技术分析治疗类风湿关节炎中成药和西药使用基本规律. 辽宁中医杂志, 2012. 39(03): 第425-427页.

[323]. 徐卫东等, 利用文本挖掘技术分析治疗类风湿关节炎中成药和西药使用基本规律. 辽宁中医杂志, 2012. 39(3): 第425-427页.

[324]. 赵钟文等, 联合雷公藤合剂治疗湿热痹阻型类风湿关节炎的临床疗效评价. 风湿病与关节炎, 2012. 1(02): 第19-24页.

[325]. 赵钟文等, 联合雷公藤合剂治疗湿热痹阻型类风湿关节炎的临床疗效评价. 风湿病与关节炎, 2012. 1(2): 第19-24页.

[326]. 邬丽娜等, 良性溃疡并发胃结肠瘘一例. 中华消化内镜杂志, 2010. 27(7): 第385-386页.

[327]. 王虹蕾与魏琴, 两种类风湿关节炎治疗方案的疗效观察. 中国误诊学杂志, 2007(09): 第1964-1965页.

[328]. 王虹蕾与魏琴, 两种类风湿关节炎治疗方案的疗效观察. 中国误诊学杂志, 2007. 7(9): 第1964-1965页.

[329]. 周玮等, 两种类风湿关节炎治疗方案的疗效及安全性比较. 实用临床医药杂志, 2017. 21(03): 第35-37页.

[330]. 周玮等, 两种类风湿关节炎治疗方案的疗效及安全性比较. 实用临床医药杂志, 2017. 21(3): 第35-37页.

[331]. 孙冬红与史群, 两种慢作用抗风湿药联合治疗类风湿关节炎临床观察. 中国药物与临床, 2003(03): 第203-204页.

[332]. 孙冬红与史群, 两种慢作用抗风湿药联合治疗类风湿关节炎临床观察. 中国药物与临床, 2003. 3(3): 第203-204页.

[333]. 郭雪松, 陈肖依与顾永军, 两组联合用药治疗类风湿关节炎的临床观察. 中国现代药物应用, 2008(20): 第4-5页.

[334]. 郭雪松, 陈肖依与顾永军, 两组联合用药治疗类风湿关节炎的临床观察. 中国现代药物应用, 2008. 2(20): 第4-5页.

[335]. 苏暄与陈明雁, 临床研究如何曲折前进?——访雷公藤多甙比较甲氨蝶呤临床试验主要设计者张烜教授. 中国医药科学, 2014. 4(08): 第4-6页.

[336]. 白雪峰与结小, 蒙药那如-3味丸治疗类风湿性关节炎疗效观察. 北方药学, 2014. 11(02): 第36-37页.

[337]. 白雪峰与结小, 蒙药那如-3味丸治疗类风湿性关节炎疗效观察. 北方药学, 2014(2): 第36-36,37页.

[338]. 魏海艳, 免疫抑制剂结合雷公藤多甙用于类风湿关节炎患者治疗临床研究. 临床医药文献电子杂志, 2018. 5(A4): 第172-173页.

[339]. 黄颖等, 苗药五藤散外敷联合西药治疗急性期寒湿阻络型类风湿关节炎随机对照临床研究. 实用中医内科杂志, 2012. 26(10): 第1-2,12页.

[340]. 陈琳, 马淑云与王新华. 帕夫林白芍总苷与雷公藤多甙治疗类风湿性关节炎的疗效比较. in 首届全国中青年风湿病学学术大会. 2004. 中国合肥.

[341]. 赵伟伟, 评价雷公藤多苷联合甲氨蝶呤治疗类风湿关节炎的疗效及安全性. 首都食品与医药, 2018. 25(22): 第38页.

[342]. 赵伟伟, 评价雷公藤多苷联合甲氨蝶呤治疗类风湿关节炎的疗效及安全性. 首都食品与医药, 2018. 25(22): 第38页.

[343]. 樊亚红等, 秦息痛片联合雷公藤多苷片、甲氨蝶呤片治疗类风湿关节炎的效果及对CXCL13、miRNA-146a、DNMTs表达水平的影响. 临床医学研究与实践, 2020. 5(35): 第21-23页.

[344]. 王永敏, 青蒿琥酯联合甲氨蝶呤、雷公藤多苷对大鼠佐剂性关节炎及滑膜PANKL、OPG表达的影响, 2009, 河北医科大学.

[345]. 李振彬等. 青蒿琥酯配伍雷公藤多甙对佐剂性关节炎大鼠血清TNF-α、IL-β表达的影响. in 海峡两岸中医药发展大会. 2009. 中国北京.

[346]. 胡晓斌等, 青霉素辅助治疗类风湿关节炎500例临床观察. 医学综述, 2011. 17(16): 第2535-2536页.

[347]. 胡晓斌等, 青霉素辅助治疗类风湿关节炎500例临床观察. 医学综述, 2011. 17(16): 第2535-2536页.

[348]. 李伟与王家祎, 曲安奈德辅以甲氨蝶呤治疗类风湿关节炎的临床疗效评价. 世界最新医学信息文摘, 2019. 19(14): 第133页.

[349]. 李伟与王家祎, 曲安奈德辅以甲氨蝶呤治疗类风湿关节炎的临床疗效评价. 世界最新医学信息文摘（连续型电子期刊）, 2019. 19(14): 第133页.

[350]. 王京利, 祛湿方熏蒸联合推拿治疗类风湿关节炎随机平行对照研究. 实用中医内科杂志, 2015. 29(03): 第151-153页.

[351]. 王京利, 祛湿方熏蒸联合推拿治疗类风湿关节炎随机平行对照研究. 实用中医内科杂志, 2015. 29(3): 第151-153页.

[352]. 焦爱军, 李振彬与宋士辉, 三七总皂苷联合雷公藤多苷对胶原诱导关节炎大鼠血管内皮生长因子表达的影响. 解放军医药杂志, 2016. 28(11): 第37-41页.

[353]. 焦爱军, 李振彬与宋士辉, 三七总皂苷联合雷公藤多苷对胶原诱导关节炎大鼠血管内皮生长因子表达的影响. 解放军医药杂志, 2016. 28(11): 第37-41页.

[354]. 柴万彪等, 桑桂通痹剂对胶原诱导关节炎小鼠病理形态学及血管内皮生长因子、基质金属蛋白酶3的影响. 中国中医药信息杂志, 2011. 18(5): 第33-35页.

[355]. 蒋昕钰与殷凯生, 沙美特罗替卡松粉吸入剂致关节痛1例. 药物流行病学杂志, 2015. 24(10): 第633-634页.

[356]. 蒋昕钰与殷凯生, 沙美特罗替卡松粉吸入剂致关节痛1例. 药物流行病学杂志, 2015. 24(10): 第633-634页.

[357]. 刘小平等, 芍甘附子汤加味对CIA寒证大鼠Egr2/Egr3及其信号通路表达的影响. 中华中医药杂志, 2018. 33(07): 第2811-2816页.

[358]. 刘小平等, 芍甘附子汤加味对CIA寒证大鼠Egr2/Egr3及其信号通路表达的影响. 中华中医药杂志, 2018. 33(7): 第2811-2816页.

[359]. 张灵与王振亮, 石藤胶囊配合甲氨蝶呤对类风湿性关节35例. 中国中医药现代远程教育, 2010. 8(13): 第174-175页.

[360]. 张灵与王振亮, 石藤胶囊配合甲氨蝶呤对类风湿性关节35例. 中国中医药现代远程教育, 2010. 08(13): 第174-175页.

[361]. 游志祥, 使用青霉素辅助治疗类风湿关节炎的效果分析. 当代医药论丛, 2015. 13(10): 第144-145页.

[362]. 游志祥, 使用青霉素辅助治疗类风湿关节炎的效果分析. 当代医药论丛, 2015(10): 第144-145页.

[363]. 刘岳, 王芳与黄慈波, 嗜酸性粒细胞增多症并高滴度类风湿因子的类风湿关节炎一例. 中华风湿病学杂志, 2015(7): 第483-484页.

[364]. 曾润铭, 刘梦璋与林菁, 四种常用抗类风湿性药物对成纤维样滑膜细胞体外增殖的影响. 中国临床康复, 2005(18): 第67-69页.

[365]. 曾润铭, 刘梦璋与林菁, 四种常用抗类风湿性药物对成纤维样滑膜细胞体外增殖的影响. 中国临床康复, 2005. 9(18): 第67-69页.

[366]. 翟相举, 探究甲氨蝶呤联合雷公藤多甙片应用于类风湿关节炎的治疗效果. 中国实用医药, 2019. 14(21): 第107-108页.

[367]. 翟相举, 探究甲氨蝶呤联合雷公藤多甙片应用于类风湿关节炎的治疗效果. 中国实用医药, 2019. 14(21): 第107-108页.

[368]. 刘浩等, 通痹合剂2号对胶原诱导型关节炎大鼠外周血CD28和CD152表达以及肿瘤坏死因子α含量的影响. 中西医结合学报, 2008(07): 第744-747页.

[369]. 刘浩等, 通痹合剂2号对胶原诱导型关节炎大鼠外周血CD28和CD152表达以及肿瘤坏死因子α含量的影响. 中西医结合学报, 2008. 6(7): 第744-747页.

[370]. 王文锋, 通痹活络汤加减联合甲氨蝶呤治疗类风湿关节炎67例临床观察. 光明中医, 2009. 24(11): 第2143-2144页.

[371]. 陈纪藩等, 通痹灵对CIA大鼠软骨细胞凋亡及其调控基因p53、Bcl-2表达作用的比较研究. 北京中医药大学学报, 2005(05): 第44-47页.

[372]. 陈纪藩等, 通痹灵对CIA大鼠软骨细胞凋亡及其调控基因p53、Bcl-2表达作用的比较研究. 北京中医药大学学报, 2005. 28(5): 第44-47页.

[373]. 吴军伟与申涛, 尪痹片治疗类风湿关节炎临床研究. 辽宁中医杂志, 2011. 38(12): 第2392-2393页.

[374]. 吴军伟与申涛, 尪痹片治疗类风湿关节炎临床研究. 辽宁中医杂志, 2011. 38(12): 第2392-2393页.

[375]. 魏红涛与郭会卿, 乌头合剂治疗类风湿性关节炎26例. 中国民族民间医药, 2017. 26(05): 第95-96页.

[376]. 魏红涛与郭会卿, 乌头合剂治疗类风湿性关节炎26例. 中国民族民间医药, 2017. 26(5): 第95-96页.

[377]. 陈世康与庞甄, 乌头汤联合西药治疗类风湿性关节炎随机平行对照研究. 实用中医内科杂志, 2015. 29(06): 第31-33页.

[378]. 陈世康与庞甄, 乌头汤联合西药治疗类风湿性关节炎随机平行对照研究. 实用中医内科杂志, 2015. 29(6): 第31-33页.

[379]. 杨利娜, 小剂量激素联合甲氨蝶呤及雷公藤多甙治疗老年类风湿关节炎的疗效观察. 中国老年保健医学, 2018. 16(01): 第79-80页.

[380]. 杨利娜, 小剂量激素联合甲氨蝶呤及雷公藤多甙治疗老年类风湿关节炎的疗效观察. 中国老年保健医学, 2018. 16(1): 第79-80页.

[381]. 陶铁铮. 小剂量来氟米特和氨甲蝶呤联合治疗类风湿关节炎. in 第六届中国中西医结合风湿病学术会议. 2006. 中国浙江义乌.

[382]. 邬亚军, 劳志英与张之澧, 小剂量雷公藤多甙和氨甲蝶呤联合治疗类风湿性关节炎的临床观察. 中国中西医结合杂志, 2001(12): 第895-896页.

[383]. 邬亚军, 劳志英与张之澧, 小剂量雷公藤多甙和氨甲蝶呤联合治疗类风湿性关节炎的临床观察. 中国中西医结合杂志, 2001. 21(12): 第895-896页.

[384]. 陈士军, 朱卫民与田培军, 小剂量雷公藤总苷联合甲氨蝶呤对类风湿关节炎患者临床症状、炎性因子及关节功能的影响. 临床误诊误治, 2020. 33(03): 第52-56页.

[385]. 陈士军, 朱卫民与田培军, 小剂量雷公藤总苷联合甲氨蝶呤对类风湿关节炎患者临床症状、炎性因子及关节功能的影响. 临床误诊误治, 2020. 33(3): 第52-56页.

[386]. 岑忠稳, 小剂量强的松+MTX+雷公藤多苷治疗RA. 医学信息（下旬刊）, 2013. 26(12): 第256页.

[387]. 张晓军等, 新风胶囊对佐剂关节炎大鼠滑膜组织缺氧诱导因子-1α、血管内皮生长因子的影响. 中医杂志, 2014. 55(05): 第416-419页.

[388]. 张晓军等, 新风胶囊对佐剂关节炎大鼠滑膜组织缺氧诱导因子-1α、血管内皮生长因子的影响. 中医杂志, 2014. 55(5): 第416-419页.

[389]. 张晓军等, 新风胶囊对佐剂性关节炎大鼠关节滑膜组织中VEGF-A mRNA表达的影响. 世界中西医结合杂志, 2013. 8(12): 第1204-1207页.

[390]. 何伟珍等, 依那西普联合雷公藤多苷治疗老年类风湿关节炎的临床观察. 中国中西医结合杂志, 2014. 34(03): 第267-271页.

[391]. 赵亚利, 依那西普联合雷公藤多苷治疗类风湿关节炎的增效减毒作用效果观察. 中国血液流变学杂志, 2017. 27(2): 第170-171页.

[392]. 郭静波等, 依那西普联合雷公藤饮片治疗类风湿关节炎的临床观察. 解放军医药杂志, 2011. 23(4): 第31-33页.

[393]. 马喜喜等, 依那西普治疗炎性关节病无效转换为英夫利西单抗后有效二例. 中华临床医师杂志（电子版）, 2014(9): 第1800-1802页.

[394]. 高建华等. 益赛普联合雷公藤多苷治疗老年类风湿关节炎的临床研究. in 全国第十一届中西医结合风湿病学术会议. 2013. 西安.

[395]. 高建华等. 益赛普联合雷公藤多苷治疗老年类风湿关节炎的临床研究. in 全国第十一届中西医结合风湿病学术会议. 2013. 中国陕西西安.

[396]. 贾宁, 原发性干燥综合征合并非霍奇金淋巴瘤, 2008, 清华大学医学部;北京协和医学院;中国医学科学院.

[397]. 温慧芬与武丽娟, 云克治疗类风湿关节炎的临床观察. 中国现代药物应用, 2016. 10(14): 第142-143页.

[398]. 温慧芬与武丽娟, 云克治疗类风湿关节炎的临床观察. 中国现代药物应用, 2016. 10(14): 第142-143页.

[399]. 李玲等, 云克治疗类风湿性关节炎——附20例分析. 天津医药, 2002. 30(5): 第310-311页.

[400]. 谷文光, 杨成林与巴智文, 镇痛活络酊结合西药治疗类风湿性关节炎临床疗效观察. 中外医疗, 2008(12): 第58-59页.

[401]. 谷文光, 杨成林与巴智文, 镇痛活络酊结合西药治疗类风湿性关节炎临床疗效观察. 中外医疗, 2008. 27(12): 第58-59页.

[402]. 张舸等, 正清风痛宁对类风湿关节炎RANTES表达的影响. 临床医药实践, 2005(12): 第893-895页.

[403]. 张舸等, 正清风痛宁对类风湿关节炎RANTES表达的影响. 临床医药实践, 2005. 14(12): 第893-895页.

[404]. 张舸与孙丽娜, 正清风痛宁对类风湿关节炎患者RANTES表达的研究. 中医药导报, 2005(11): 第23-25页.

[405]. 张舸与孙丽娜, 正清风痛宁对类风湿关节炎患者RANTES表达的研究. 中医药导报, 2005. 11(11): 第17-19页.

[406]. 刘旭东等, 正清风痛宁片联合雷公藤多苷片治疗老年活动性类风湿关节炎52例. 中国中医药现代远程教育, 2015. 13(3): 第53-54页.

[407]. 陈兴等, 治疗类风湿性关节炎的民间偏方. 世界最新医学信息文摘（连续型电子期刊）, 2015(56): 第6-8页.

[408]. 中成药或将改变类风湿治疗策略. 河北中医, 2014. 36(04): 第564页.

[409]. 韩曼, 中国类风湿关节炎患者报告的临床结局量表优化及应用研究, 2017, 中国中医科学院.

[410]. 陶锡东与陆红日, 中西药联合强化治疗重度活动性类风湿关节炎60例. 风湿病与关节炎, 2013. 2(09): 第41-42页.

[411]. 陶锡东与陆红日, 中西药联合强化治疗重度活动性类风湿关节炎60例. 风湿病与关节炎, 2013(9): 第41-42页.

[412]. 陈哲等, 中西药治疗类风湿关节炎随机对照的临床研究. 中国康复, 2011. 26(06): 第415-417页.

[413]. 陈哲等, 中西药治疗类风湿关节炎随机对照的临床研究. 中国康复, 2011. 18(6): 第415-417页.

[414]. 董焱与曾雅萍, 中药蜡疗联合雷公藤多甙及甲氨蝶呤治疗活动性类风湿关节炎的效果观察. 中国实用医刊, 2020(01): 第109-110-111页.

[415]. 董焱与曾雅萍, 中药蜡疗联合雷公藤多甙及甲氨蝶呤治疗活动性类风湿关节炎的效果观察. 中国实用医刊, 2020. 47(1): 第109-111页.

[416]. 章可谓, 中药内服外贴联合西药治疗类风湿关节炎49例. 中医药学报, 2008. 36(06): 第59-60页.

[417]. 章可谓, 中药内服外贴联合西药治疗类风湿关节炎49例. 中医药学报, 2008. 36(6): 第59-60页.

[418]. 陈晓君, 中药熏蒸辅助治疗活动性类风湿关节炎的临床研究, 2016, 大连医科大学.

[419]. 任世元, 中药熏蒸治疗类风湿性关节炎活动期79例临床观察. 湖南中医杂志, 2018. 34(10): 第64-66页.

[420]. 任世元, 中药熏蒸治疗类风湿性关节炎活动期79例临床观察. 湖南中医杂志, 2018. 34(10): 第64-66页.

[421]. 吴方真等. 中医药优化方案治疗湿热痹阻型活动期RA的临床观察. in 全国第十二届中西医结合风湿病学术会议. 2014. 天津.

[422]. 吴方真等, 中医药优化方案治疗湿热痹阻型活动期类风湿关节炎临床观察. 中医药通报, 2014. 13(06): 第40-42+39页.

[423]. 吴方真等, 中医药优化方案治疗湿热痹阻型活动期类风湿关节炎临床观察. 中医药通报, 2014(6): 第40-42,39页.

[424]. 郭燕芬等, 中医药优化方案治疗湿热痹阻型类风湿关节炎的不良反应观察. 风湿病与关节炎, 2014. 3(11): 第15-19页.

[425]. 郭燕芬等, 中医药优化方案治疗湿热痹阻型类风湿关节炎的不良反应观察. 风湿病与关节炎, 2014(11): 第15-19页.

[426]. 顾镭等, 重组人Ⅱ型肿瘤坏死因子受体-抗体融合蛋白治疗类风湿关节炎出现巩膜炎一例. 中华风湿病学杂志, 2009. 13(10): 第723-724,插页10-2页.

[427]. 顾镭等, 重组人Ⅱ型肿瘤坏死因子受体-抗体融合蛋白治疗类风湿关节炎出现巩膜炎一例. 中华风湿病学杂志, 2009. 13(10): 第723-724,插页10-2页.

[428]. 向永国, 桂枝芍药知母汤加味治疗类风湿性关节炎疗效观察. 四川中医, 2016. 34(7): 第111-113页.

[429]. 向永国, 桂枝芍药知母汤加味治疗类风湿性关节炎疗效观察. 四川中医, 2016. 34(07): 第111-113页.

[430]. 向永国, 桂枝芍药知母汤加味治疗类风湿性关节炎疗效观察. 四川中医, 2016. 34(7): 第111-113页.

[431]. 陈用军与段逸群. 桂枝芍药知母汤抗炎及免疫调节作用机制. in 中华中医药学会皮肤科分会第四次学术年会;全国中医、中西医结合皮肤病诊疗新进展高级研修班. 2007. 中国新疆乌鲁木齐.

[432]. 陈用军与段逸群. 桂枝芍药知母汤抗炎及免疫调节作用机制. in 中华中医药学会皮肤科分会第四次学术年会;全国中医、中西医结合皮肤病诊疗新进展高级研修班. 2007. 中国新疆乌鲁木齐.

[433]. 杨锦屏, 杜烨辉与安静思. 激素及环磷酰胺冲击联合治疗类风湿关节炎合并肾脏损害18例分析. in 第十届全国风湿病学学术会议. 2005.

[434]. 杨锦屏, 杜烨辉与安静思. 激素及环磷酰胺冲击联合治疗类风湿关节炎合并肾脏损害18例分析. in 第十届全国风湿病学学术会议. 2005.

[435]. 布文才, 加减桂枝芍药知母汤治疗类风湿关节炎临床研究. 亚太传统医药, 2015. 11(06): 第135-136页.

[436]. 布文才, 加减桂枝芍药知母汤治疗类风湿关节炎临床研究. 亚太传统医药, 2015. 11(6): 第135-136页.

[437]. 陈鹏等, 雷公藤多苷联合甲氨蝶呤治疗类风湿关节炎的随机对照试验. 安徽中医学院学报, 2011. 30(6): 第28-32页.

[438]. 陈鹏等, 雷公藤多苷联合甲氨蝶呤治疗类风湿关节炎的随机对照试验. 安徽中医学院学报, 2011. 30(06): 第28-32页.

[439]. 陈鹏等, 雷公藤多苷联合甲氨蝶呤治疗类风湿关节炎的随机对照试验. 安徽中医学院学报, 2011. 30(6): 第28-32页.

[440]. 王在红, 杨金玲与王露, 雷公藤多苷联合甲氨蝶呤治疗类风湿关节炎患者的效果. 中国民康医学, 2020. 32(20): 第74-76页.

[441]. 王在红, 杨金玲与王露, 雷公藤多苷联合甲氨蝶呤治疗类风湿关节炎患者的效果. 中国民康医学, 2020. 32(20): 第74-76页.

[442]. 陈伏宇, 于广莹与王慧, 雷公藤多苷片、新癀片合用治疗湿热瘀阻型类风湿关节炎80例. 中医临床研究, 2010. 2(22): 第86-87页.

[443]. 陈伏宇, 于广莹与王慧, 雷公藤多苷片、新癀片合用治疗湿热瘀阻型类风湿关节炎80例. 中医临床研究, 2010. 02(22): 第86-87页.

[444]. 谷敬欣等, 雷公藤多苷片联合甲氨蝶呤对类风湿关节炎合并骨质疏松患者血清骨代谢标志物水平及炎症因子的影响. 现代中西医结合杂志, 2020. 29(22): 第2424-2428页.

[445]. 谷敬欣等, 雷公藤多苷片联合甲氨蝶呤对类风湿关节炎合并骨质疏松患者血清骨代谢标志物水平及炎症因子的影响. 现代中西医结合杂志, 2020. 29(22): 第2424-2428页.

[446]. 谷敬欣等, 雷公藤多苷片联合甲氨蝶呤对类风湿关节炎合并骨质疏松患者血清骨代谢标志物水平及炎症因子的影响. 现代中西医结合杂志, 2020. 29(22): 第2424-2428页.

[447]. 罗波等, 雷公藤甲素对佐剂性关节炎大鼠外周血单个核细胞核因子κB受体激活剂配基表达的影响. 华中科技大学学报·医学版, 2006. 35(2): 第265-267页.

[448]. 罗波等, 雷公藤甲素对佐剂性关节炎大鼠外周血单个核细胞核因子κB受体激活剂配基表达的影响. 华中科技大学学报(医学版), 2006(02): 第265-267页.

[449]. 罗波等, 雷公藤甲素对佐剂性关节炎大鼠外周血单个核细胞核因子κB受体激活剂配基表达的影响. 华中科技大学学报（医学版）, 2006. 35(2): 第265-267页.

[450]. 王虹蕾与魏琴, 两种类风湿关节炎治疗方案的疗效观察. 中国误诊学杂志, 2007. 7(9): 第1964-1965页.

[451]. 王虹蕾与魏琴, 两种类风湿关节炎治疗方案的疗效观察. 中国误诊学杂志, 2007(09): 第1964-1965页.

[452]. 王虹蕾与魏琴, 两种类风湿关节炎治疗方案的疗效观察. 中国误诊学杂志, 2007. 7(9): 第1964-1965页.

[453]. 孙冬红与史群, 两种慢作用抗风湿药联合治疗类风湿关节炎临床观察. 中国药物与临床, 2003. 3(3): 第203-204页.

[454]. 孙冬红与史群, 两种慢作用抗风湿药联合治疗类风湿关节炎临床观察. 中国药物与临床, 2003(03): 第203-204页.

[455]. 孙冬红与史群, 两种慢作用抗风湿药联合治疗类风湿关节炎临床观察. 中国药物与临床, 2003. 3(3): 第203-204页.

[456]. 魏海艳, 免疫抑制剂结合雷公藤多甙用于类风湿关节炎患者治疗临床研究. 临床医药文献电子杂志, 2018. 5(A4): 第172-173页.

[457]. 魏海艳, 免疫抑制剂结合雷公藤多甙用于类风湿关节炎患者治疗临床研究. 临床医药文献电子杂志, 2018. 5(A4): 第172-173页.

[458]. 黄颖等, 苗药五藤散外敷联合西药治疗急性期寒湿阻络型类风湿关节炎随机对照临床研究. 实用中医内科杂志, 2012. 26(14): 第1-2+12页.

[459]. 黄颖等, 苗药五藤散外敷联合西药治疗急性期寒湿阻络型类风湿关节炎随机对照临床研究. 实用中医内科杂志, 2012. 26(10): 第1-2,12页.

[460]. 赵伟伟, 评价雷公藤多苷联合甲氨蝶呤治疗类风湿关节炎的疗效及安全性. 首都食品与医药, 2018. 25(22): 第38页.

[461]. 赵伟伟, 评价雷公藤多苷联合甲氨蝶呤治疗类风湿关节炎的疗效及安全性. 首都食品与医药, 2018. 25(22): 第38页.

[462]. 赵伟伟, 评价雷公藤多苷联合甲氨蝶呤治疗类风湿关节炎的疗效及安全性. 首都食品与医药, 2018. 25(22): 第38页.

[463]. 王永敏, 青蒿琥酯联合甲氨蝶呤、雷公藤多苷对大鼠佐剂性关节炎及滑膜PANKL、OPG表达的影响, 2009, 河北医科大学.

[464]. 王永敏, 青蒿琥酯联合甲氨蝶呤、雷公藤多苷对大鼠佐剂性关节炎及滑膜PANKL、OPG表达的影响, 2009, 河北医科大学.

[465]. 李振彬等. 青蒿琥酯配伍雷公藤多甙对佐剂性关节炎大鼠血清TNF-α、IL-β表达的影响. in 海峡两岸中医药发展大会. 2009. 中国北京.

[466]. 李振彬等. 青蒿琥酯配伍雷公藤多甙对佐剂性关节炎大鼠血清TNF-α、IL-β表达的影响. in 海峡两岸中医药发展大会. 2009. 中国北京.

[467]. 胡晓斌等, 青霉素辅助治疗类风湿关节炎500例临床观察. 医学综述, 2011. 17(16): 第2535-2536页.

[468]. 胡晓斌等, 青霉素辅助治疗类风湿关节炎500例临床观察. 医学综述, 2011. 17(16): 第2535-2536页.

[469]. 胡晓斌等, 青霉素辅助治疗类风湿关节炎500例临床观察. 医学综述, 2011. 17(16): 第2535-2536页.

[470]. 李伟与王家祎, 曲安奈德辅以甲氨蝶呤治疗类风湿关节炎的临床疗效评价. 世界最新医学信息文摘, 2019(14): 第133页.

[471]. 李伟与王家祎, 曲安奈德辅以甲氨蝶呤治疗类风湿关节炎的临床疗效评价. 世界最新医学信息文摘, 2019. 19(14): 第133页.

[472]. 李伟与王家祎, 曲安奈德辅以甲氨蝶呤治疗类风湿关节炎的临床疗效评价. 世界最新医学信息文摘（连续型电子期刊）, 2019. 19(14): 第133页.

[473]. 刘岳, 王芳与黄慈波, 嗜酸性粒细胞增多症并高滴度类风湿因子的类风湿关节炎一例. 中华风湿病学杂志, 2015. 19(07): 第483-484+506页.

[474]. 刘岳, 王芳与黄慈波, 嗜酸性粒细胞增多症并高滴度类风湿因子的类风湿关节炎一例. 中华风湿病学杂志, 2015(7): 第483-484页.

[475]. 陶铁铮. 小剂量来氟米特和氨甲蝶呤联合治疗类风湿关节炎. in 第六届中国中西医结合风湿病学术会议. 2006. 中国浙江义乌.

[476]. 陶铁铮. 小剂量来氟米特和氨甲蝶呤联合治疗类风湿关节炎. in 第六届中国中西医结合风湿病学术会议. 2006. 中国浙江义乌.

[477]. 陈哲等, 中西药治疗类风湿关节炎随机对照的临床研究. 中国康复, 2011. 26(6): 第415-417页.

[478]. 陈哲等, 中西药治疗类风湿关节炎随机对照的临床研究. 中国康复, 2011. 26(06): 第415-417页.

[479]. 陈哲等, 中西药治疗类风湿关节炎随机对照的临床研究. 中国康复, 2011. 18(6): 第415-417页.

[480]. 章可谓, 中药内服外贴联合西药治疗类风湿关节炎49例. 中医药学报, 2008. 36(6): 第59-60页.

[481]. 章可谓, 中药内服外贴联合西药治疗类风湿关节炎49例. 中医药学报, 2008. 36(06): 第59-60页.

[482]. 陈晓君, 中药熏蒸辅助治疗活动性类风湿关节炎的临床研究, 2016, 大连医科大学.

[483]. 陈晓君, 中药熏蒸辅助治疗活动性类风湿关节炎的临床研究, 2016, 大连医科大学.

[484]. 顾镭等, 重组人Ⅱ型肿瘤坏死因子受体-抗体融合蛋白治疗类风湿关节炎出现巩膜炎一例. 中华风湿病学杂志, 2009(10): 第723-724+730页.

[485]. 顾镭等, 重组人Ⅱ型肿瘤坏死因子受体-抗体融合蛋白治疗类风湿关节炎出现巩膜炎一例. 中华风湿病学杂志, 2009(10): 第723-724+730页.

**201 non-TG**

[1]. 龙武彬等, ~(99)Tc-MDP治疗类风湿关节炎30例. 中国药业, 2002(05): 第74-75页.

[2]. 薛光彦, ~(99)Tc-亚甲基二磷酸盐在类风湿关节炎治疗中的临床观察. 吉林医学, 2011. 32(12): 第360页.

[3]. 龙武彬等, 99Tc-MDP治疗类风湿关节炎30例. 中国药业, 2002. 11(5): 第74-75页.

[4]. Cibere, J., et al., A randomized double blind, placebo controlled trial of topical Tripterygium wilfordii in rheumatoid arthritis: Reanalysis using logistic regression analysis. Journal of Rheumatology, 2003. 30(3): p. 465-467.

[5]. Glickman-Simon, R. and J. Steurich, Acupuncture for Ischemic Stroke, Music for Anxiety in Mechanical Ventilation, Essential Fatty Acids for Depression, Mindfulness Meditation for Sleep in Older Adults, Tripterygium wilfordii for Rheumatoid Arthritis. Explore: The Journal of Science and Healing, 2016. 12(1): p. 71-75.

[6]. Ye, Z., et al., Clinical study on etanercept combined with tripterygium wilfordii polyglycoside for treatment of eldly rheumatoid arthritis. Annals of the Rheumatic Diseases, 2013. 72.

[7]. Ye, Z., et al., Clinical study on etanercept combined with Tripterygium wilfordii polyglycoside for treatment of eldly rheumatoid arthritis. International Journal of Rheumatic Diseases, 2013. 16: p. 58.

[8]. Landewé, R.B. and D. van der Heijde, Comment on: 'comparison of Tripterygium wilfordii Hook F with methotrexate in the treatment of active rheumatoid arthritis (TRIFRA): a randomised, controlled clinical trial' by Qian-wen et al. Ann Rheum Dis, 2014. 73(10): p. e62.

[9]. Lü, A.P., et al., Correlations of clinical symptoms and treatment efficacy in patients with rheumatoid arthritis treated with Chinese herbal drugs or Western medicine. 2005. 3(6): p. 432‐437.

[10]. NCT, Effectiveness and Safety of Yisaipu Combined With Tripterygium Wilfordii for Active RA. 2018.

[11]. NCT, Efficacy and Safety of Tripterygium Wilfordii in Patients With Rheumatoid Arthritis. 2012.

[12]. NCT, Efficacy and Safety of TwHF and MTX in Patients With Rheumatoid Arthritis. 2017.

[13]. Macfarlane, G.J., et al., Evidence for the efficacy of complementary and alternative medicines in the management of rheumatoid arthritis: A systematic review. Rheumatology, 2011. 50(9): p. 1672-1683.

[14]. Yang, C. and F. Huang, Management of spondyloarthropathy in Asian countries. Current Rheumatology Reviews, 2008. 4(2): p. 131-134.

[15]. Yuan, K., et al., Paying attention to the preciseness of conclusion. Ann Rheum Dis, 2014. 73(11): p. e66.

[16]. Zha, Q.L., et al., Predictive role of diagnostic information in treatment efficacy of rheumatoid arthritis based on neural network model analysis. Journal of Chinese Integrative Medicine, 2007. 5(1): p. 32-38.

[17]. Lv, Q.W., et al., Response to D.M. Marcus's comment on the TRIFRA study (comparison of Tripterygium wilfordii Hook F vs methotrexate in the treatment of active rheumatoid arthritis). Ann Rheum Dis, 2014. 73(9): p. e57.

[18]. Liu, Y.F., et al., The Derivative of Tripterygium wilfordii Hook F - Kunxian Capsule, Attenuated Rheumatoid Arthritis: A Systematic Review and Meta-Analysis. Evidence-based Complementary and Alternative Medicine, 2020. 2020.

[19]. Lv, Q.W., et al., The TRIFRA trial: efforts employed to minimise expectation bias. Ann Rheum Dis, 2014. 73(11): p. e67.

[20]. Luo, J., et al., Total glucosides of paeony for rheumatoid arthritis: A systematic review of randomized controlled trials. Complementary Therapies in Medicine, 2017. 34: p. 46-56.

[21]. Lian, F., et al., Tripterygium WILFORDII multiglycoside combined regimen as long-term maintenance therapy in chinese rheumatoid arthritis patients. Annals of the Rheumatic Disease, 2013. 71.

[22]. 钱瑾, 阿达木单抗联合雷公藤多甙对甲氨蝶呤治疗反应不佳活动性类风湿关节炎的效果及安全性分析. 中西医结合心血管病电子杂志, 2019. 7(35): 第33-34页.

[23]. 李娟等, 艾拉莫德与甲氨蝶呤配伍治疗老年类风湿关节炎84例. 陕西医学杂志, 2016. 45(1): 第120-121页.

[24]. 夏楠楠, 陈忠锋与张伟峰, 艾拉莫德与雷公藤多苷片治疗类风湿关节炎的效果观察. 实用中西医结合临床, 2020. 20(11): 第76-77页.

[25]. 王轶等, 白芍总苷、甲氨喋呤及雷公藤治疗类风湿性关节炎150例体会. 郑州大学学报·医学版, 2006. 41(5): 第1002-1003页.

[26]. 李兴锐, 陈茂红与王和融, 白芍总苷合雷公藤多苷治疗类风湿关节炎60例. 安徽中医学院学报, 2011. 30(3): 第16-18页.

[27]. 许武, 白芍总苷胶囊联合雷公藤片治疗类风湿关节炎疗效和安全性分析. 现代诊断与治疗, 2017. 28(13): 第2404-2406页.

[28]. 曾祯, 辨证治疗类风湿关节炎76例. 中国民族民间医药杂志, 2010. 19(15): 第171页.

[29]. 彭剑虹与叶雪英, 补肾养阴通络方联合甲氨喋呤治疗类风湿关节炎20例. 中医研究, 2010. 23(3): 第33-35页.

[30]. 梁启迪, 段勇明与秦超, 补益肝肾汤联合常规疗法对类风湿性关节炎患者血清炎症因子及生活质量的影响. 四川中医, 2019. 37(7): 第143-146页.

[31]. 吴启富等. 不同联合治疗方案对类风湿关节炎远期疗效的追踪观察与分析. in 第六届中国中西医结合风湿病学术会议. 2006. 中国浙江义乌.

[32]. 姜慧晶, 除痹汤联合膝关节围刺对寒热错杂型类风湿性关节炎疗效观察. 中国中医药现代远程教育, 2020. 18(4): 第J0143-J0145页.

[33]. 周腊梅等, 从病例分析RA慢作用药物治疗方案选择. 西藏科技, 2017(3): 第38-41页.

[34]. 李玲与王莘智, 从寒热辨证探讨雷公藤片对类风湿性关节炎的临床疗效. 亚太传统医药, 2020. 16(3): 第140-143页.

[35]. 钟洁珠, 大剂量甲基强的松龙治疗类风湿关节炎的疗效观察. 临床合理用药杂志, 2013. 6(5): 第43-44页.

[36]. 温旭升, 低剂量雷公藤多苷在早期类风湿关节炎的疗效研究. 维吾尔医药(上半月), 2013(4): 第245-246页.

[37]. 刘清平等, 断藤益母汤对育龄期女性类风湿关节炎患者卵巢功能的影响. 中医杂志, 2015. 56(20): 第1746-1749页.

[38]. 杨通宇等, 飞金止痛胶囊联合甲氨蝶呤和雷公藤多苷治疗类风湿关节炎的临床观察. 中国药房, 2016. 27(23): 第3218-3220页.

[39]. 高泽林与王新宏, 肺间质病变32例临床分析. 陕西医学杂志, 2007. 36(8): 第1033-1034页.

[40]. 张磊, 风湿骨痛贴方治疗类风湿关节炎临床研究, 2016, 河北大学.

[41]. 周颖芳, 黄丽军与刘步平. 蜂针治疗类风湿关节炎Meta分析. in 中国民族医药学会蜂疗分会第一届中医、民族医蜂疗学术交流大会(2016年). 2016. 北京.

[42]. 张海霞, 附子汤联合甲氨蝶呤治疗寒湿痹阻型类风湿关节炎的临床研究, 2018, 内蒙古医科大学.

[43]. 陈岳祥等, 复方粉背雷公藤治疗类风湿关节炎的临床观察. 华南国防医学杂志, 2009. 23(2): 第4-5,11页.

[44]. 孟彪, 复方雷公藤药酒治疗类风湿关节炎(寒湿痹阻证)的临床研究.

[45]. 戴洁梅等, 复方雷公藤逐痛颗粒辅助治疗痰瘀互结型类风湿关节炎的临床效果观察. 现代生物医学进展, 2019. 19(4): 第743-746页.

[46]. 马艳, 复方雪莲胶囊治疗类风湿关节炎寒湿痹阻证的临床观察, 2016, 新疆医科大学.

[47]. 陈小玉, 复方追风伞治疗老年类风湿关节炎的临床观察及其实验研究, 2016, 贵阳中医学院.

[48]. 刘婧依等, 戈利木单抗联合雷公藤多甙对甲氨蝶呤治疗反应不佳活动性类风湿关节炎的效果及安全性分析. 现代生物医学进展, 2017. 17(9): 第1735-1738,1725页.

[49]. 康尔恂等, 关节病性银屑病52例临床分析. 临床皮肤科杂志, 2004. 33(11): 第665-667页.

[50]. 金香花, 刘畅与雷蕾, 观察比较两种类风湿关节炎治疗方案的疗效及安全性. 中国实用医药, 2020. 15(26): 第153-154页.

[51]. 陈霸琼, 桂枝芍药知母汤加减方治疗类风湿关节炎寒热错杂证的临床研究, 2012, 南京中医药大学.

[52]. 向永国, 桂枝芍药知母汤加味治疗类风湿性关节炎疗效观察. 四川中医, 2016. 34(7): 第111-113页.

[53]. 阎晓霞, 赵志强与仝允辉, 化瘀通痹熏蒸方联合西药治疗类风湿关节炎30例. 中医研究, 2015. 28(4): 第17-19页.

[54]. 达其伟等, 黄芪桂枝五物汤加减内服、熏蒸联合西药治疗类风湿关节炎30例. 中医研究, 2015. 28(9): 第10-13页.

[55]. 韩玉凤, 基于关节超声评价断藤益母汤联合来氟米特治疗类风湿关节炎的临床疗效, 2017, 广州中医药大学.

[56]. 杨锦屏, 杜烨辉与安静思. 激素及环磷酰胺冲击联合治疗类风湿关节炎合并肾脏损害18例分析. in 第十届全国风湿病学学术会议. 2005.

[57]. 汉辉传, 加减桂枝芍药知母汤治疗类风湿关节炎疗效观察及生存质量的临床研究, 2013, 南京中医药大学.

[58]. 王晋英, 加减桂枝芍药知母汤治疗类风湿关节炎临床观察. 医药前沿, 2018. 8(33): 第337-338页.

[59]. 布文才, 加减桂枝芍药知母汤治疗类风湿关节炎临床研究. 亚太传统医药, 2015. 11(6): 第135-136页.

[60]. 苏秀芳与赵利平, 加味桂枝汤治疗产后类风湿性关节炎临床观察. 山西中医, 2013. 29(8): 第12-13,19页.

[61]. 刘泉, 魏成义与李肾荣, 甲氨喋呤、雷公藤治疗类风湿性关节炎61例临床分析. 中华医学写作杂志, 2000. 7(8): 第870-871页.

[62]. 倪斐, 甲氨喋呤联合雷公藤多甙片治疗类风湿性关节炎的效果. 心理月刊, 2018(02): 第270页.

[63]. 肖峰, 甲氨喋呤联合雷公藤多甙片治疗类风湿性关节炎效果及药物副反应分析. 中外医学研究, 2018. 16(10): 第28-29页.

[64]. 谭玉珍, 吴彩玲与游运辉, 甲氨喋呤联合青霉胺治疗类风湿性关节炎临床分析. 中国现代医学杂志, 2000. 10(12): 第71-72页.

[65]. 贾雪阳, 甲氨蝶呤、来氟米特、环磷酰胺两两联合疗效比较研究, 2011, 山西医科大学.

[66]. 国同河, 甲氨蝶呤和来氟米特及雷公藤多甙片小剂量联合应用、治疗类风湿性关节炎的临床观察. 中华现代内科学杂志, 2009. 6(2): 第96-98页.

[67]. 李烨, 甲氨蝶呤和雷公藤多甙联合治疗类风湿关节炎的临床观察. 现代医药卫生, 2007. 23(5): 第639-640页.

[68]. 朱春来与罗观, 甲氨蝶呤和硫酸软骨素联合雷公藤小剂量治疗类风湿关节炎临床疗效观察. 中国社区医师·医学专业, 2012. 14(12): 第135页.

[69]. 莫美丽等, 甲氨蝶呤联合艾拉莫德治疗活动性类风湿关节炎的随机对照试验. 福建医科大学学报, 2018. 52(4): 第245-248页.

[70]. 贾娜·沙里江, 王彦焱与罗莉, 甲氨蝶呤联合锝[~(99)Tc]亚甲基二膦酸盐治疗类风湿关节炎58例临床评价. 中国药业, 2015. 24(24): 第48-50页.

[71]. 沙里江, 贾., 王彦焱与罗莉, 甲氨蝶呤联合锝[99Tc]亚甲基二膦酸盐治疗类风湿关节炎58例临床评价. 中国药业, 2015(24): 第48-50页.

[72]. 贾娜·沙里江, 王彦焱与罗莉, 甲氨蝶呤联合锝[99Tc]亚甲基二膦酸盐治疗类风湿关节炎58例临床评价. 中国药业, 2015. 24(24): 第48-50页.

[73]. 孙玮与刘秀梅, 甲氨蝶呤联合来氟米特或雷公藤多苷治疗抗环瓜氨酸肽抗体阳性的早期类风湿关节炎的疗效比较. 山西医药杂志·上半月, 2010(1): 第59-60页.

[74]. 张晓等, 甲氨蝶呤联合来氟米特或羟基氯喹治疗类风湿关节炎的临床观察. 中华医学杂志, 2004. 84(12): 第1038-1040页.

[75]. 徐丽华, 甲氨蝶呤联合来氟米特及雷公藤多甙治疗类风湿关节炎65例临床观察. 医学信息（下旬刊）, 2010. 23(11): 第64-64页.

[76]. 常胜军, 甲氨蝶呤联合来氟米特及雷公藤多甙治疗类风湿性关节炎61例疗效观察. 医药与保健（中旬版）, 2009. 17(9): 第101-102页.

[77]. 朱琳与陈鹏, 甲氨蝶呤联合雷公藤多苷与单用甲氨蝶呤治疗类风湿关节炎效果比较. 检验医学与临床, 2015. 12(23): 第3568-3570页.

[78]. 秦理, 杨孝兵与蒋峰. 甲氨蝶呤联合雷公藤多苷治疗绝经后类风湿关节炎. in 二零一四年浙江省风湿病学学术年会. 2014. 中国浙江湖州.

[79]. 罗义根等, 甲氨蝶呤联合柳氮磺胺吡啶、雷公藤多甙片治疗类风湿关节炎疗效观察. 长江大学学报·自然科学版(医学)(下旬), 2014. 11(11): 第39-42页.

[80]. 朱芳晓, 周润华与石宇红, 甲氨蝶呤联合羟氯喹或雷公藤多苷治疗抗环瓜氨酸肽抗体阳性的早期类风湿关节炎的临床研究. 中国医药指南, 2012. 10(18): 第416-418页.

[81]. 李念羊, 吴宁宁与张培, 甲氨蝶呤联合曲安奈德治疗类风湿关节炎的效果分析. 中国当代医药, 2013. 20(23): 第85-86,88页.

[82]. 卜晋安, 甲氨蝶呤联合中药治疗类风湿性关节炎22例临床疗效分析. 中国厂矿医学, 2008. 21(4): 第432-433页.

[83]. 周伯炜, 甲氨蝶呤与雷公藤多甙片联合治疗类风湿关节炎的 临床效果及安全性. 家庭医药, 2018(8): 第146-147页.

[84]. 黄静等, 甲氨蝶呤与雷公藤多苷分别联合来氟米特治疗类风湿关节炎的效果比较. 药物评价研究, 2020(1): 第103-106页.

[85]. 李思吟与邓代华, 甲氨蝶呤治疗类风湿性关节炎的应用及最佳剂量分析. 内江科技, 2018. 39(09): 第65-66+48页.

[86]. 樊文萍, 丁素银与贾淑霞, 甲基强的松龙治疗早期类风湿关节炎36例临床效果分析. 医学研究与教育, 2009. 26(3): 第39,41页.

[87]. 刘健, 李华与谌曦, 健脾化湿通络法治疗类风湿关节炎贫血的临床研究. 中西医结合学报, 2006. 4(4): 第348-354页.

[88]. 刘丽敏, 姜酚胶丸治疗类风湿关节炎临床与实验研究, 2012, 贵阳中医学院.

[89]. 高照猛等, 抗苗勒管激素水平评价生育期女性类风湿关节炎患者卵巢储备功能的临床意义. 中国现代医生, 2018. 56(14): 第21-26页.

[90]. 郑建寅等, 矿泉中药浴治疗类风湿性关节炎临床疗效评价. 中国疗养医学, 2000. 9(1): 第15-18页.

[91]. 王启芬, 昆明山海棠根茎不同投药部位对二藤通痹合剂抗炎药效的比较研究, 2015, 广州中医药大学.

[92]. 范仰钢与李国华, 昆明山海棠联合甲氨蝶呤治疗老年起病类风湿关节炎. 现代医药卫生, 2006. 22(4): 第478-480页.

[93]. 林昌松等, 昆仙胶囊联合甲氨蝶呤治疗类风湿关节炎疗效观察. 陕西中医, 2010(8): 第987-990页.

[94]. 蒋建湘, 来氟米特联合甲氨喋呤治疗类风湿关节炎临床观察. 临床合理用药杂志, 2018. 11(16): 第31-32,34页.

[95]. 朱建琴与钱建东, 来氟米特联合甲氨蝶呤治疗类风湿关节炎的疗效观察. 医药前沿, 2016(4): 第192-193页.

[96]. 卢红平, 来氟米特联合甲氨蝶呤治疗难治性类风湿关节炎疗效观察. 现代中西医结合杂志, 2013. 22(18): 第1979-1980页.

[97]. 沈杰与张之澧, 雷公藤多甙合小剂量甲氨蝶呤治疗老年性类风湿性关节炎. 浙江中西医结合杂志, 2002. 12(6): 第334-336页.

[98]. 张小翠, 雷公藤多甙联合来氟米特及甲氨蝶呤治疗类风湿关节炎疗效观察. 山西中医, 2017. 33(9): 第28-29,34页.

[99]. 吴敏与马英淳. 雷公藤多甙联合柳氮磺吡啶治疗老年类风湿关节炎的临床观察. in 第十届全国风湿病学学术会议. 2005.

[100]. 王志强等, 雷公藤多苷不同时间给药联合甲氨蝶呤治疗难治性类风湿关节炎疗效观察. 现代中西医结合杂志, 2020. 29(1): 第11-14,54页.

[101]. 李英, 雷公藤多苷联合氨甲蝶呤与来氟米特治疗类风湿关节炎患者的效果. 中国民康医学, 2020. 32(10): 第85-86页.

[102]. 张跃军, 雷公藤多苷联合甲氨蝶呤治疗类风湿关节炎的临床分析. 中国卫生标准管理, 2020. 11(12): 第101-103页.

[103]. 潘祝平, 林顺平与林旋, 雷公藤多苷联合甲氨蝶呤治疗类风湿关节炎短期疗效观察. 风湿病与关节炎, 2014. 3(3): 第17-20页.

[104]. 林顺平. 雷公藤多苷联合甲氨蝶呤治疗类风湿关节炎疗效评价. in 第17次全国风湿病学学术会议. 2012. 中国云南昆明.

[105]. 李娜与汲泓, 雷公藤多苷联合柳氮磺胺吡啶对活动期类风湿性关节炎相关指标的影响. 辽宁中医药大学学报, 2008. 10(12): 第89-90页.

[106]. 陈继红, 劳志英与何东仪, 雷公藤多苷联合青藤碱治疗类风湿关节炎49例. 上海中医药杂志, 2011. 45(10): 第64-65页.

[107]. 莫菁莲, 王政与张瑞城, 雷公藤多苷联合依那西普治疗老年类风湿关节炎及其对血清炎性因子和骨代谢因子的影响. 中国老年学杂志, 2020. 40(13): 第2810-2812页.

[108]. 王丽华, 雷公藤多苷配伍甲氨蝶呤及来氟米特治疗类风湿关节炎的临床研究. 世界最新医学信息文摘, 2019(62): 第209,211页.

[109]. 张婉, 雷公藤多苷配伍甲氨蝶呤及来氟米特治疗类风湿性关节炎的临床效果分析. 国际免疫学杂志, 2018. 41(2): 第243-246页.

[110]. 陈伏宇, 于广莹与王慧, 雷公藤多苷片、新癀片合用治疗湿热瘀阻型类风湿关节炎80例. 中医临床研究, 2010. 2(22): 第86-87页.

[111]. 谭贵基, 雷公藤多苷片联合甲氨蝶呤治疗类风湿关节炎的疗效观察. 健康之路, 2013. 12(10): 第218-219页.

[112]. 杜金万, 杜川与郑利强, 雷公藤多苷片治疗类风湿关节炎对患者免疫蛋白的影响. 临床合理用药杂志, 2019. 12(13): 第35-37页.

[113]. 宋绍亮与马宏洋, 雷公藤复方预防类风湿关节炎患者依那西普诱导缓解后复发效果观察. 山东医药, 2014. 54(36): 第97-98页.

[114]. 赵钟文等, 雷公藤合剂配合手关节操治疗活动巅类风湿关节炎临床研究. 中医药通报, 2014. 13(4): 第40-44页.

[115]. 赵钟文等, 雷公藤合剂配合手关节操治疗活动期类风湿关节炎临床研究. 中医药通报, 2014. 13(04): 第40-44页.

[116]. 李广涛, 雷公藤甲素和甲氨喋呤治疗类风湿关节炎的生命质量与临床疗效比较. 中国中医药咨讯, 2010(35): 第69,94页.

[117]. 陈德超等, 雷公藤甲素治疗老年类风湿关节炎的临床效果体会. 医药前沿, 2013(24): 第127-128页.

[118]. 李萍, 雷公藤炮制后在临床中的应用. 中国医药指南, 2019. 17(4): 第160页.

[119]. 杨竹, 雷公藤片治疗类风湿性关节炎74例. 中国药业, 2011. 20(14): 第76-77页.

[120]. 余效福与余培红, 雷公藤药酒治疗类风湿性关节炎148例临床研究. 医学信息(西安), 2015(27): 第317页.

[121]. 余效福, 雷公藤药酒治疗难治性类风湿性关节炎48例临床研究. 宁夏医学杂志, 2020. 42(3): 第281-283页.

[122]. 黄少弼, 肖征宇与曾庆馀, 雷公藤与其它慢作用抗风湿药治疗类风湿关节炎65例. 中国医师杂志, 2001. 3(3): 第182-183页.

[123]. 王娟芳, 雷公藤治疗类风湿性关节炎的疗效观察. 内蒙古中医药, 2017. 36(4): 第31页.

[124]. 吴厅与宋欣伟, 类风湿Ⅰ号联合西药治疗湿热阻络型类风湿关节炎临床研究. 新中医, 2019. 51(7): 第163-166页.

[125]. 任璇璇等, 类风湿Ⅰ号丸增加高度活动类风湿关节炎脾气亏虚、湿热阻络证疗效的临床观察. 中华中医药学刊, 2014. 32(3): 第578-581页.

[126]. 胡怀霞, 类风湿关节炎老年患者的用药安全性分析. 医药前沿, 2014(29): 第183-184页.

[127]. 陈小庄与吴苗青, 类风湿性关节炎联合药物治疗体会. 中国热带医学, 2006. 6(3): 第472-473页.

[128]. 赵钟文等, 联合雷公藤合剂治疗湿热痹阻型类风湿关节炎的临床疗效评价. 风湿病与关节炎, 2012. 1(2): 第19-24页.

[129]. 张鸿逵等, 联合药物治疗类风湿性关节炎89例. 山东医药, 1993(12): 第21-22页.

[130]. 李昌与吴燕生, 联合治疗类风湿性关节炎160例临床分析. 中国社区医师·综合版, 2006. 8(2): 第56页.

[131]. 王虹蕾与魏琴, 两种类风湿关节炎治疗方案的疗效观察. 中国误诊学杂志, 2007. 7(9): 第1964-1965页.

[132]. 周玮等, 两种类风湿关节炎治疗方案的疗效及安全性比较. 实用临床医药杂志, 2017. 21(3): 第35-37页.

[133]. 孙冬红与史群, 两种慢作用抗风湿药联合治疗类风湿关节炎临床观察. 中国药物与临床, 2003. 3(3): 第203-204页.

[134]. 郭雪松, 陈肖依与顾永军, 两组联合用药治疗类风湿关节炎的临床观察. 中国现代药物应用, 2008. 2(20): 第4-5页.

[135]. 白雪峰与结小, 蒙药那如-3味丸治疗类风湿性关节炎疗效观察. 北方药学, 2014. 11(2): 第36-37页.

[136]. 魏海艳, 免疫抑制剂结合雷公藤多甙用于类风湿关节炎患者治疗临床研究. 临床医药文献电子杂志, 2018. 5(A4): 第172-173页.

[137]. 黄颖等, 苗药五藤散外敷联合西药治疗急性期寒湿阻络型类风湿关节炎随机对照临床研究. 实用中医内科杂志, 2012. 26(10): 第1-2,12页.

[138]. 黄颖等, 苗药五藤散外敷联合西药治疗急性期寒湿阻络型类风湿关节炎随机对照临床研究. 实用中医内科杂志, 2012. 26(14): 第1-2+12页.

[139]. 林星等, 难治性类风湿关节炎60例分析. 现代诊断与治疗, 2009. 20(3): 第165-166页.

[140]. 王轶等, 帕夫林、MTX及雷公藤对RA临床指标影响的观察. 中国老年保健医学, 2005. 3(4): 第51-53页.

[141]. 陈琳, 马淑云与王新华. 帕夫林白芍总苷与雷公藤多甙治疗类风湿性关节炎的疗效比较. in 首届全国中青年风湿病学学术大会. 2004. 中国合肥.

[142]. 胡晓斌等, 青霉素辅助治疗类风湿关节炎500例临床观察. 医学综述, 2011. 17(16): 第2535-2536页.

[143]. 李伟与王家祎, 曲安奈德辅以甲氨蝶呤治疗类风湿关节炎的临床疗效评价. 世界最新医学信息文摘, 2019(14): 第133页.

[144]. 杨鑫, 祛风除湿、益气养血法治疗类风湿关节炎合并贫血的临床研究, 2013, 南京中医药大学.

[145]. 王京利, 祛湿方熏蒸联合推拿治疗类风湿关节炎随机平行对照研究. 实用中医内科杂志, 2015(3): 第151-153页.

[146]. 马学玉, 忍冬翘薇汤联合免疫抑制剂治疗类风湿性关节炎25例临床观察. 中华实用中西医杂志, 2011. 24(5): 第1-2页.

[147]. 袁作武与周艳华, 散寒清热通痹片治疗类风湿关节炎寒热错杂证80例. 中医研究, 2015. 28(6): 第8-10页.

[148]. 钟岩, 石亚妹与武丽君, 生物制剂联合传统抗风湿药物治疗类风湿关节炎的临床疗效观察. 新疆医学, 2017. 47(7): 第710-713页.

[149]. 张灵与王振亮, 石藤胶囊配合甲氨蝶呤对类风湿性关节35例. 中国中医药现代远程教育, 2010(13): 第174-175页.

[150]. 王振亮与指导姚乃礼, 石藤胶囊治疗类风湿性关节炎30例临床观察. 中华中医药杂志, 2011. 26(3): 第528-530页.

[151]. 王振亮与姚乃礼, 石藤胶囊治疗类风湿性关节炎30例临床观察. 中华中医药杂志, 2011. 26(03): 第528-530页.

[152]. 王振亮, 石藤胶囊治疗类风湿性关节炎30例临床观察. 中华中医药杂志, 2011. 26(3): 第528-530页.

[153]. 游志祥, 使用青霉素辅助治疗类风湿关节炎的效果分析. 当代医药论丛, 2015(10): 第144-145页.

[154]. 周德荣, 搜骨清血法治疗类风湿性关节炎临床疗效观察. 中国中医药信息杂志, 2005. 12(6): 第68-69页.

[155]. 王文锋, 通痹活络汤加减联合甲氨蝶呤治疗类风湿关节炎67例临床观察. 光明中医, 2009(11): 第2143-2144页.

[156]. 陈红明, 通痹雷公藤汤配合马钱胶囊治疗类风湿性关节炎39例观察. 实用中医药杂志, 2009(8): 第514-515页.

[157]. 吴军伟与申涛, 尪痹片治疗类风湿关节炎临床研究. 辽宁中医杂志, 2011. 38(12): 第2392-2393页.

[158]. 宋明霞, 温针灸配合药物治疗类风湿性关节炎临床疗效观察, 2014, 上海中医药大学.

[159]. 沈菁等, 温针灸治疗类风湿性关节炎的临床文献研究. 湖南中医杂志, 2015(9): 第142-143,150页.

[160]. 魏红涛与郭会卿, 乌头合剂治疗类风湿性关节炎26例. 中国民族民间医药杂志, 2017(5): 第95-96页.

[161]. 陈世康与庞甄, 乌头汤联合西药治疗类风湿性关节炎随机平行对照研究. 实用中医内科杂志, 2015(6): 第31-33页.

[162]. 方振玉, 小剂量激素联合甲氨蝶呤及雷公藤多甙治疗RA的临床研究, 2014, 扬州大学.

[163]. 杨利娜, 小剂量激素联合甲氨蝶呤及雷公藤多甙治疗老年类风湿关节炎的疗效观察. 中国老年保健医学, 2018. 16(1): 第79-80页.

[164]. 陶铁铮. 小剂量来氟米特和氨甲蝶呤联合治疗类风湿关节炎. in 第六届中国中西医结合风湿病学术会议. 2006. 中国浙江义乌.

[165]. 邬亚军, 劳志英与张之澧, 小剂量雷公藤多甙和氨甲蝶呤联合治疗类风湿性关节炎的临床观察. 中国中西医结合杂志, 2001. 21(12): 第895-896页.

[166]. 岑忠稳, 小剂量强的松+MTX+雷公藤多苷治疗RA. 医学信息（下旬刊）, 2013. 26(6): 第256页.

[167]. 何伟珍等, 依那西普联合雷公藤多苷治疗老年类风湿关节炎的临床观察. 中国中西医结合杂志, 2014. 34(3): 第267-271页.

[168]. 赵亚利, 依那西普联合雷公藤多苷治疗类风湿关节炎的增效减毒作用效果观察. 中国血液流变学杂志, 2017. 27(2): 第170-171页.

[169]. 郭静波等, 依那西普联合雷公藤饮片治疗类风湿关节炎的临床观察. 解放军医药杂志, 2011. 23(04): 第31-33页.

[170]. 高建华等. 益赛普联合雷公藤多苷治疗老年类风湿关节炎的临床研究. in 全国第十一届中西医结合风湿病学术会议. 2013. 中国陕西西安.

[171]. 孟庆良, 郭会卿与赵一静, 益肾蠲痹汤合甲氨喋呤治疗类风湿性关节炎40例. 中医研究, 2006. 19(8): 第27-29页.

[172]. 何伟珍等. 英夫利昔单抗联合雷公藤多苷治疗老年类风湿关节炎. in 第十六届中国中西医结合风湿病学术年会. 2018. 中国广东广州.

[173]. 陈松, 李明与张俊辉, 硬膜外阻滞治疗类风湿性关节炎的疗效观察. 中外健康文摘, 2011. 08(15): 第228-229页.

[174]. 温慧芬与武丽娟, 云克治疗类风湿关节炎的临床观察. 中国现代药物应用, 2016. 10(14): 第142-143页.

[175]. 李玲等, 云克治疗类风湿性关节炎——附20例分析. 天津医药, 2002(05): 第310-311页.

[176]. 谷文光, 杨成林与巴智文, 镇痛活络酊结合西药治疗类风湿性关节炎临床疗效观察. 中外医疗, 2008. 27(12): 第58-59页.

[177]. 刘旭东等, 正清风痛宁片联合雷公藤多苷片治疗老年活动性类风湿关节炎52例. 中国中医药现代远程教育, 2015(3): 第53-54页.

[178]. 刘旭东等, 正清风痛宁片联合雷公藤多苷片治疗老年活动性类风湿关节炎52例. 中国中医药现代远程教育, 2015. 13(03): 第53-54页.

[179]. 吴凡玉与李红艳, 正清风痛宁治疗骨关节炎临床观察. 中国医学创新, 2009. 6(20): 第116-117页.

[180]. 陶锡东与陆红日, 中西药联合强化治疗重度活动性类风湿关节炎60例. 风湿病与关节炎, 2013. 2(9): 第41-42页.

[181]. 陈哲等, 中西药治疗类风湿关节炎随机对照的临床研究. 中国康复, 2011. 26(6): 第415-417页.

[182]. 何海洲与李伯英, 中西医结合治疗活动期类风湿关节炎. 吉林中医药, 2017. 37(5): 第477-481页.

[183]. 忻霞菲与陈勇, 中西医结合治疗老年类风湿性关节炎. 浙江中西医结合杂志, 2005. 15(5): 第292-293页.

[184]. 黄品良, 中西医结合治疗类风湿性关节炎43例. 湖北中医杂志, 2004. 26(11): 第24页.

[185]. 秦新艳. 中西医综合疗法治疗白种人类风湿关节炎的疗效及安全性评价. in 全国自身免疫性疾病专题研讨会暨第十一次全国风湿病学学术年会. 2006. 中国江苏南京.

[186]. 董焱与曾雅萍, 中药蜡疗联合雷公藤多甙及甲氨蝶呤治疗活动性类风湿关节炎的效果观察. 中国实用医刊, 2020(1): 第109-111页.

[187]. 黄湘颖等, 中药蜡疗联合西药治疗类风湿关节炎31例观察. 浙江中医杂志, 2018. 53(4): 第289-290页.

[188]. 章可谓, 中药内服外贴联合西药治疗类风湿关节炎49例. 中医药学报, 2008. 36(6): 第59-60页.

[189]. 刘晓萌, 中药汽疗熏蒸治疗活动期类风湿关节炎（寒湿痹阻证）的临床疗效观察, 2014, 黑龙江中医药大学.

[190]. 陈晓君, 中药熏蒸辅助治疗活动性类风湿关节炎的临床研究, 2016, 大连医科大学.

[191]. 任世元, 中药熏蒸治疗类风湿性关节炎活动期79例临床观察. 湖南中医杂志, 2018. 34(10): 第64-66页.

[192]. 吴方真等. 中医药优化方案治疗湿热痹阻型活动期RA的临床观察. in 全国第十二届中西医结合风湿病学术会议. 2014. 中国天津.

[193]. 吴方真等, 中医药优化方案治疗湿热痹阻型活动期类风湿关节炎临床观察. 中医药通报, 2014. 13(6): 第40-42,39页.

[194]. 郭燕芬等, 中医药优化方案治疗湿热痹阻型类风湿关节炎的不良反应观察. 风湿病与关节炎, 2014. 3(11): 第15-19页.

[195]. 彭丽岚, 张传俊与侯斌, 诸畀灵治疗类风湿性关节炎疗效观察. 中华临床医学杂志, 2006. 7(10): 第17-19页.

[196]. 刘占全等, 注射用氢化可的松琥珀酸钠治疗早期类风湿关节炎34例. 中国城乡企业卫生, 2008(3): 第45页.

[197]. 钱瑾, 阿达木单抗联合雷公藤多甙对甲氨蝶呤治疗反应不佳活动性类风湿关节炎的效果及安全性分析. 中西医结合心血管病电子杂志, 2019. 7(35): 第33-34页.

[198]. 李娟等, 艾拉莫德与甲氨蝶呤配伍治疗老年类风湿关节炎84例. 陕西医学杂志, 2016. 45(1): 第120-121页.

[199]. 夏楠楠, 陈忠锋与张伟峰, 艾拉莫德与雷公藤多苷片治疗类风湿关节炎的效果观察. 实用中西医结合临床, 2020. 20(11): 第76-77页.

[200]. 李兴锐, 陈茂红与王和融, 白芍总苷合雷公藤多苷治疗类风湿关节炎60例. 安徽中医学院学报, 2011. 30(3): 第16-18页.

[201]. 许武, 白芍总苷胶囊联合雷公藤片治疗类风湿关节炎疗效和安全性分析. 现代诊断与治疗, 2017. 28(13): 第2404-2406页.

**101 primary efficacy point is not ACR20, ACR50, ACR70**

[1]. 徐佳, （5R）-5-羟基雷公藤内酯醇的吸收机制和与甲氨蝶呤联合用药的药物—药物相互作用研究, 2014, 华东理工大学.

[2]. 田亚伟, 1,25(OH)_2D_3在类风湿关节炎治疗中的作用研究, 2018, 河南科技大学.

[3]. 吴庆军等, 5例抗丙氨酰tRNA合成酶抗体阳性患者临床特征. 中华临床免疫和变态反应杂志, 2014. 8(2): 第129-133页.

[4]. 薛光彦, 99Tc-亚甲基二磷酸盐在类风湿关节炎治疗中的临床观察. 吉林医学, 2011. 32(12): 第2360页.

[5]. Wang, J.X. and C.G. Du, A retrospective study of clinical characteristics of interstitial lung disease associated with rheumatoid arthritis in Chinese patients. Medical Science Monitor, 2015. 21: p. 708-715.

[6]. 李宗英, BLyS、MCP-1在类风湿关节炎中的表达及其与肺间质纤维化的关系研究, 2013, 河北医科大学.

[7]. Venkatesha, S.H. and K.D. Moudgil, Celastrol and its role in controlling chronic diseases. 2016. p. 267-289.

[8]. Zeng, R.M., M.Z. Liu and J. Lin, Effects of four anti-rheumatoid arthritis drugs on proliferation of fibroblast-like synoviocytes in vitro. Chinese Journal of Clinical Rehabilitation, 2005. 9(18): p. 67-69.

[9]. Liu, J., H. Li and X. Chen, Effects of traditional Chinese medicine for invigorating spleen to resolve dampness and dredging collaterals on patients with rheumatoid arthritis and anemia. Journal of Chinese Integrative Medicine, 2006. 4(4): p. 348-354.

[10]. 卢文艺, FoxO1介导黄藤酒治疗类风湿关节炎的实验研究, 2019, 长江大学.

[11]. Zheng, K., et al., Hei-Gu-Teng zhuifenghuoluo granule modulates IL-12 signal pathway to inhibit the inflammatory response in rheumatoid arthritis. Journal of Immunology Research, 2018. 2018.

[12]. 田伟兰等, MTX联合不同中药对Ⅱ型胶原诱导关节炎大鼠血清TNF-α及RORγt mRNA表达的影响. 中华中医药杂志, 2016. 31(10): 第4184-4187页.

[13]. Koo, H. and C.D. Morrow, Perturbation of the human gastrointestinal tract microbial ecosystem by oral drugs to treat chronic disease results in a spectrum of individual specific patterns of extinction and persistence of dominant microbial strains. PLoS One, 2020. 15(12): p. e0242021.

[14]. Ma, L., et al., Reduced numbers of regulatory B cells are negatively correlated with disease activity in patients with new-onset rheumatoid arthritis. Clinical Rheumatology, 2014. 33(2): p. 187-195.

[15]. Zhang, F., et al., Rheumatoid arthritis analysis by nash equilibrium game analysis. Journal of Medical Imaging and Health Informatics, 2019. 9(7): p. 1382-1385.

[16]. Zhu, T., et al., Systemic sclerosis-rheumatoid arthritis overlap syndrome complicated with Sweet’s syndrome. Clinical Rheumatology, 2018. 37(8): p. 2281-2284.

[17]. 张珊, 丹参酮ⅡA通过调节中性粒细胞活性治疗类风湿关节炎的研究, 2017, 北京中医药大学.

[18]. 陈晓俊, 杜仲提取物对CIA大鼠的骨保护作用及机制研究, 2017, 上海中医药大学.

[19]. 刘清平, 断藤益母汤对类风湿关节炎患者卵巢功能作用的临床及实验研究.

[20]. 卢军, 断藤益母汤及其组方药物对胶原诱导性关节炎大鼠骨代谢的影响及其机制研究, 2016, 广州中医药大学.

[21]. 陈利锋等, 复方芪芎颗粒对佐剂性关节炎大鼠抗炎作用的实验研究. 华南国防医学杂志, 2015(6): 第415-418页.

[22]. 陈用军与段逸群. 桂枝芍药知母汤抗炎及免疫调节作用机制. in 中华中医药学会皮肤科分会第四次学术年会;全国中医、中西医结合皮肤病诊疗新进展高级研修班. 2007. 中国新疆乌鲁木齐.

[23]. 陈用军, 桂枝芍药知母汤抗炎及免疫调节作用机制的实验研究, 2007, 湖北中医学院.

[24]. 陈利锋等, 黄芪注射液合川芎嗪注射液治疗类风湿关节炎的作用机理研究. 中国中医急症, 2012. 21(6): 第924-925,928页.

[25]. 王林, 基于DAS28、CDAI的类风湿关节炎凝血/纤溶指标与中医证型及其疾病活动度的相关性研究, 2015, 北京中医药大学.

[26]. 刘史佳等, 基于PK-PD模型研究雷公藤治疗类风湿关节炎生物靶标. 中国中药杂志, 2015. 40(2): 第334-338页.

[27]. 雷旭杰, 基于骨代谢水平对断藤益母汤治疗中老年类风湿关节炎的疗效观察, 2018, 广州中医药大学.

[28]. 汪元与刘健, 基于神经内分泌免疫网络学说探讨新风胶囊治疗类风湿关节炎的量效关系. 时珍国医国药, 2010. 21(10): 第2622-2624页.

[29]. 王强, 基于网络药理及分子对接探讨断藤益母汤靶向MAP3K2治疗类风湿关节炎的作用机制, 2019, 广州中医药大学.

[30]. 刘宁涛等, 甲氨喋呤和雷公藤对类风湿关节炎滑膜细胞产生趋化因子的影响. 第四军医大学学报, 2006. 27(12): 第1113-1115页.

[31]. 王威等, 甲氨喋吟联合中药对胶原诱导关节炎大鼠血清VEGF及滑膜VEGF mRNA表达的影响. 中华中医药杂志, 2016. 31(2): 第451-454页.

[32]. 刘星与张华银, 甲氨蝶呤加雷公藤多甙片对类风湿关节炎的治疗作用研究. 当代医学, 2017. 23(29): 第71-72页.

[33]. 雷尚文等, 甲氨蝶呤联合雷公藤多苷对中老年类风湿关节炎患者的疗效及对金属蛋白酶-3的影响. 甘肃医药, 2020. 39(06): 第513-516页.

[34]. 崔毅佳, 王淑梅与金志国, 甲氨蝶呤通过抑制TLR2-NF-κB信号通路减轻类风湿关节炎大鼠滑膜炎的作用研究. 新疆医科大学学报, 2019. 42(2): 第211-216页.

[35]. 铁宁与张桂芝, 甲氨蝶呤与雷公藤多苷片联用对类风湿性关节炎大鼠的治疗作用. 中国中医急症, 2016. 25(4): 第655-657,674页.

[36]. 李丹, 艾浩与李晓明, 金雀异黄素协同雷公藤多苷干预去卵巢大鼠CIA的机制研究. 转化医学电子杂志, 2017. 4(1): 第35-38页.

[37]. 王迪等, 金雀异黄素协同雷公藤多苷治疗去卵巢大鼠类风湿关节炎的药效学研究. 世界科学技术·中医药现代化, 2013. 15(6): 第1343-1347页.

[38]. 黄华, 金乌健骨汤对寒湿型类风湿关节炎的观察及对CIA大鼠IL-17/IL-23的影响, 2013, 贵阳中医学院.

[39]. 唐志宇与梁江, 昆山合剂对类风湿关节炎合并贫血患者滑膜成纤维细胞增殖及MyD88、IL-6表达的研究. 世界科学技术·中医药现代化, 2014(3): 第582-586页.

[40]. 王笑丹, 昆仙胶囊治疗类风湿关节炎临床疗效评价及对CIA大鼠IL-8、γIP-10影响, 2011, 广州中医药大学.

[41]. 陈宗良, 雷公藤多甙对滑膜细胞趋化因子RANTES、MCP-1影响的研究. 中国医疗前沿, 2009(13): 第125,139页.

[42]. 孙凤艳, 雷公藤多甙对类风湿关节炎患者滤泡辅助性T细胞的影响.

[43]. 赵玲, 雷公藤多甙联合丹参酮ⅡA对胶原诱导关节炎大鼠心血管损伤的保护作用及机制探讨, 2008, 河北医科大学.

[44]. 谭晴心与肖琴, 雷公藤多甙联合甲氨蝶呤治疗类风湿关节炎疗效评价及对TNF-α、IL-6的影响. 中国中医药信息杂志, 2010. 17(9): 第7-9页.

[45]. 宋哲, 雷公藤多苷对大鼠胶原诱导关节炎的时间治疗学研究, 2016, 华北理工大学.

[46]. 杨宏伟, 涂胜豪与常栋, 雷公藤多苷对胶原诱导性关节炎大鼠中高迁移率族蛋白B1影响的研究. 中华风湿病学杂志, 2011. 15(8): 第550-552,585页.

[47]. 刘巍与张艳艳, 雷公藤多苷对类风湿关节炎患者成纤维样滑膜细胞α7nAChR及炎症因子的作用. 山东中医杂志, 2019. 38(12): 第1166-1170,1197页.

[48]. 张艳艳. 雷公藤多苷对类风湿关节炎患者成纤维样滑膜细胞胆碱能抗炎通路的作用. in 2017年第五次世界中西医结合大会. 2017. 中国广东广州.

[49]. 孙凤艳等, 雷公藤多苷对类风湿关节炎患者滤泡辅助性T细胞及IL-21的影响. 医学综述, 2016. 22(3): 第566-569页.

[50]. 周明华, 雷公藤多苷对类风湿性关节炎患者血清VEGF、VEGFR2表达水平的影响研究. 中国保健营养, 2016. 26(5): 第304页.

[51]. 罗波等, 雷公藤多苷对佐剂性关节炎模型大鼠关节中核因子κB受体激活剂配基表达的影响. 医药导报, 2006. 25(5): 第395-397页.

[52]. 胡旭君与宋欣伟, 雷公藤多苷联合甲氨蝶呤对干燥综合征NOD小鼠治疗作用及TNF-α、IL-1β、AQP-5的表达. 中华中医药杂志, 2014. 29(7): 第2362-2366页.

[53]. 冯艳广与王晓寒, 雷公藤多苷联合甲氨蝶呤对类风湿关节炎患者TfH细胞及IL-21的影响. 慢性病学杂志, 2017(11): 第1296-1298页.

[54]. 胡文娟, 雷公藤多苷联合甲氨蝶呤对类风湿性关节炎患者血清CRP ESR RF水平的影响. 基层医学论坛, 2020. 24(32): 第4647-4648页.

[55]. 张红与薛中柱, 雷公藤多苷联合甲氨蝶呤治疗类风湿性关节炎短期疗效研究. 吉林中医药, 2018. 38(6): 第660-663页.

[56]. 张秋媛, 雷公藤多苷联合人参皂苷对胶原诱导关节炎大鼠CD4~+CD25~+Foxp3~+调节性T细胞的影响及骨免疫学机制探讨, 2012, 河北医科大学.

[57]. 辛立波, 雷公藤多苷联合人参皂苷对巨噬细胞移动抑制因子诱导大鼠成纤维样滑膜细胞增殖及RANKL/OPG表达的影响, 2011, 河北医科大学.

[58]. 马华, 雷公藤多苷联合人参皂苷对佐剂性关节炎大鼠的骨保护作用及机制探讨, 2010, 河北医科大学.

[59]. 马华等, 雷公藤多苷联合双醋瑞因对类风湿关节炎中IL-1、TNF-α、OPG、RANKL的影响. 中国继续医学教育, 2016. 8(36): 第137-139页.

[60]. 谷敬欣等, 雷公藤多苷片联合甲氨蝶呤对类风湿关节炎合并骨质疏松患者血清骨代谢标志物水平及炎症因子的影响. 现代中西医结合杂志, 2020. 29(22): 第2424-2428页.

[61]. 郇稳等, 雷公藤多苷片联合甲氨蝶呤治疗类风湿关节炎对患者骨代谢及血清炎性因子水平的影响. 中国实用医刊, 2020(03): 第90-91-92-93页.

[62]. 刘敏等, 雷公藤多苷治疗类风湿性关节炎的临床观察及对血清VEGF、VEGFR2表达水平的影响研究. 陕西中医, 2016(1): 第72-74页.

[63]. 卢嘉微等, 雷公藤红素对胶原诱导性关节炎小鼠的免疫作用研究. 南京中医药大学学报, 2018. 34(05): 第491-494页.

[64]. 林娜. 雷公藤活性成分的抗风湿病情改善作用及机理研究. in 第八届全国临床中药学学术年会. 2015. 哈尔滨.

[65]. 罗波等, 雷公藤甲素对佐剂性关节炎大鼠外周血单个核细胞核因子κB受体激活剂配基表达的影响. 华中科技大学学报·医学版, 2006. 35(2): 第265-267页.

[66]. 范文强等, 雷公藤甲素治疗类风湿关节炎的作用机制及安全性分析. 中草药, 2019. 50(16): 第3866-3871页.

[67]. 马俊福等, 雷公藤内酯醇对胶原诱导性关节炎大鼠γ-干扰素和白细胞介素-17A表达的影响. 北京中医药大学学报, 2020. 43(07): 第592-598页.

[68]. 王玉等, 雷公藤内酯醇对佐剂关节炎模型大鼠关节中核因子-κB受体激活剂配基表达的影响. 安徽中医学院学报, 2007. 26(3): 第28-30页.

[69]. 孙文文等. 类风湿关节炎合并肺间质病变的发病相关因素. in 山东省第九次风湿病学学术会议暨风湿免疫疾病生物治疗进展学习班. 2012. 中国山东济南.

[70]. 于风明, 类叶牡丹抗类风湿性关节炎有效部位免疫抑制作用初步研究, 2016, 黑龙江中医药大学.

[71]. 吕邵娃等, 类叶牡丹提取物对大鼠佐剂性关节炎治疗作用及机制研究. 中药新药与临床药理, 2017. 28(2): 第164-171页.

[72]. 许丹, 类叶牡丹有效部位干预胶原诱导型关节炎大鼠的代谢组学研究, 2018, 黑龙江中医药大学.

[73]. 那仁满都拉, 蒙药忠伦阿汤干预活动期RA临床疗效观察及对CIA模型大鼠免疫调节机制的影响, 2015, 北京中医药大学.

[74]. 樊亚红等, 秦息痛片联合雷公藤多苷片、甲氨蝶呤片治疗类风湿关节炎的效果及对CXCL13、miRNA-146a、DNMTs表达水平的影响. 临床医学研究与实践, 2020. 5(35): 第21-23页.

[75]. 王永敏, 青蒿琥酯联合甲氨蝶呤、雷公藤多苷对大鼠佐剂性关节炎及滑膜PANKL、OPG表达的影响, 2009, 河北医科大学.

[76]. 李振彬等. 青蒿琥酯配伍雷公藤多甙对佐剂性关节炎大鼠血清TNF-α、IL-β表达的影响. in 海峡两岸中医药发展大会. 2009. 中国北京.

[77]. 李剑明, 清热活血方对CIA大鼠滑膜组织B细胞浸润程度及IL-21、CXCL13表达的影响, 2019, 中国中医科学院.

[78]. 焦爱军, 李振彬与宋士辉, 三七总皂苷联合雷公藤多苷对胶原诱导关节炎大鼠血管内皮生长因子表达的影响. 解放军医药杂志, 2016. 28(11): 第37-41页.

[79]. 柴万彪等, 桑桂通痹剂对胶原诱导关节炎小鼠病理形态学及血管内皮生长因子、基质金属蛋白酶3的影响. 中国中医药信息杂志, 2011. 18(05): 第33-35页.

[80]. 刘小平等, 芍甘附子汤加味对CIA寒证大鼠Egr2/Egr3及其信号通路表达的影响. 中华中医药杂志, 2018. 33(7): 第2811-2816页.

[81]. 王振亮与指导姚乃礼, 石藤胶囊对类风湿性关节炎患者OPG和RANKL的影响. 辽宁中医杂志, 2011. 38(3): 第422-423页.

[82]. 王振亮与姚乃礼, 石藤胶囊对类风湿性关节炎患者OPG和RANKL的影响. 辽宁中医杂志, 2011. 38(03): 第422-423页.

[83]. 王振亮, 石藤胶囊对类风湿性关节炎患者OPG和RANKL的影响. 辽宁中医杂志, 2011. 38(3): 第422-423页.

[84]. 曾润铭, 刘梦璋与林菁, 四种常用抗类风湿性药物对成纤维样滑膜细胞体外增殖的影响. 中国临床康复, 2005. 9(18): 第67-69页.

[85]. 刘浩等, 通痹合剂2号对胶原诱导型关节炎大鼠外周血CD28和CD152表达以及肿瘤坏死因子α含量的影响. 中西医结合学报, 2008. 6(7): 第744-747页.

[86]. 刘浩, 通痹合剂2号疗效观察及对B7/CD28作用的研究, 2008, 广州中医药大学.

[87]. 陈纪藩等, 通痹灵对CIA大鼠软骨细胞凋亡及其调控基因p53、Bcl-2表达作用的比较研究. 北京中医药大学学报, 2005. 28(5): 第44-47页.

[88]. 张永红, 顽痹清方治疗类风湿关节炎湿热闭阻证的疗效评价及对细胞因子调节作用的研究.

[89]. 赵晓倩, 温经清络益肾方治疗类风湿关节炎寒热错杂证的临床观察及对破骨细胞相关受体的影响, 2019, 南京中医药大学.

[90]. 李育林, 五藤冲剂干预类风湿关节炎的作用机理研究.

[91]. 张北雪, 五种“术”类健脾和抗风湿的药效学和代谢组学研究, 2020, 辽宁中医药大学.

[92]. 刘健等, 新风胶囊对类风湿关节炎贫血的疗效及机制研究. 中国临床保健杂志, 2010. 13(3): 第225-229页.

[93]. 张晓军等, 新风胶囊对佐剂关节炎大鼠滑膜组织缺氧诱导因子-1α、血管内皮生长因子的影响. 中医杂志, 2014. 55(5): 第416-419页.

[94]. 张晓军等, 新风胶囊对佐剂性关节炎大鼠关节滑膜组织中VEGF-A mRNA表达的影响. 世界中西医结合杂志, 2013. 8(12): 第1204-1207页.

[95]. 张育, 炎性关节病的基础与临床系列研究.

[96]. 熊智, 羊踯躅根抗类风湿性关节炎药理作用评价及其化学成分研究, 2012, 上海中医药大学.

[97]. 张舸等, 正清风痛宁对类风湿关节炎RANTES表达的影响. 临床医药实践, 2005. 14(12): 第893-895页.

[98]. 张舸与孙丽娜, 正清风痛宁对类风湿关节炎患者RANTES表达的研究. 中医药导报, 2005. 11(11): 第17-19页.

[99]. 陈哲, 中医证候与代谢组学在雷公藤个体化治疗类风湿关节炎中的研究, 2011, 华中科技大学.

[100]. 陈哲立, 中医证候与代谢组学在雷公藤个体化治疗类风湿关节炎中的研究, 2011, 华中科技大学.

[101]. 张永红, 紫草素对RA滑膜成纤维细胞COX-2 mRNA表达的影响, 2011, 哈尔滨医科大学.

[102]. 刘松涛, 紫草素对RA滑膜成纤维细胞TNF-α、IL-6表达的影响, 2011, 哈尔滨医科大学.

[103]. 铁宁与张桂芝, 甲氨蝶呤与雷公藤多苷片联用对类风湿性关节炎大鼠的治疗作用. 中国中医急症, 2016. 25(4): 第655-657,674页.

[104]. 谭晴心与肖琴, 雷公藤多甙联合甲氨蝶呤治疗类风湿关节炎疗效评价及对TNF-α、IL-6的影响. 中国中医药信息杂志, 2010. 17(9): 第7-9页.

[105]. 胡旭君与宋欣伟, 雷公藤多苷联合甲氨蝶呤对干燥综合征NOD小鼠治疗作用及TNF-α、IL-1β、AQP-5的表达. 中华中医药杂志, 2014. 29(7): 第2362-2366页.

**105 experimental sutdies**

[1]. 史晓飞等, 1,25二羟维生素D 3在老年类风湿关节炎治疗中的作用. 河南科技大学学报·医学版, 2019. 37(3): 第212-215页.

[2]. NCT, A Specification Clinical Application Research of Tripterygium Wilfordii Treating Rheumatoid Arthritis. 2017.

[3]. Landewé, R.B.M. and D. van der Heijde, Comment on: 'Comparison of Tripterygium wilfordii Hook F with methotrexate in the treatment of active rheumatoid arthritis (TRIFRA): A randomised, controlled clinical trial' by Qian-wen et al. Annals of the Rheumatic Diseases, 2014.

[4]. Marcus, D.M., Comparison of Tripterygium wilfordii Hook F with methotrexate in the treatment of rheumatoid arthritis. Ann Rheum Dis, 2014. 73(9): p. e56.

[5]. Yang, J., et al., Effect of vitamin D on the recurrence rate of rheumatoid arthritis. Experimental and Therapeutic Medicine, 2015. 10(5): p. 1812-1816.

[6]. Zhang, W., et al., The safety and effectiveness of a chloroform/methanol extract of Tripterygium wilfordii Hook F (T2) plus methotrexate in treating rheumatoid arthritis. J Clin Rheumatol, 2010. 16(8): p. 375-8.

[7]. Wang, X., et al., Treatment of rheumatoid arthritis with combination of methotrexate and Tripterygium wilfordii: A meta-analysis. Life Sci, 2017. 171: p. 45-50.

[8]. NCT, Tripterygium Wilfordii Hook F and Methotrexate for Postmenopausal Women With Rheumatoid Arthritis. 2019.

[9]. 李井华与高净, 甲氨喋呤(MTX)联合雷公藤多苷片治疗类风湿关节炎的疗效及安全性分析. 中国保健营养, 2017. 27(21): 第279-280页.

[10]. 史晓与陈建军, 甲氨喋呤加雷公藤治疗类风湿性关节炎临床观察. 河北中西医结合杂志, 1997. 6(5): 第727-728页.

[11]. 冯泉, 甲氨喋呤联合雷公藤多苷片治疗类风湿性关节炎的疗效及不良反应分析. 中国现代药物应用, 2017. 11(16): 第131-132页.

[12]. 李培跃, 甲氨喋呤片联合雷公藤多甙片治疗类风湿性关节炎的作用评价. 养生保健指南, 2019(52): 第261页.

[13]. 欧秋娟, 黄存军与廖湘平, 甲氨蝶呤和雷公藤多甙联合对类风湿关节炎的治疗效果评估. 内蒙古中医药, 2016. 35(17): 第50-51页.

[14]. 和雅等, 甲氨蝶呤加雷公藤多甙片对类风湿关节炎的效果对比研究. 泰山医学院学报, 2019. 40(10): 第775-776页.

[15]. 丁丽萍与时永强, 甲氨蝶呤加用雷公藤多甙片治疗类风湿性关节炎的临床效果观察. 医药前沿, 2018. 8(16): 第147页.

[16]. 廖子鸿, 周志华与戴冠东, 甲氨蝶呤加用雷公藤多甙片治疗类风湿性关节炎的临床效果探析. 中国生化药物杂志, 2016. 36(5): 第136-138页.

[17]. 王莺莺, 金美红与王卫斌, 甲氨蝶呤联合雷公藤多甙对类风湿性关节炎干预的整体疗效研究. 中国生化药物杂志, 2017. 37(4): 第385-387页.

[18]. 郭翠敏, 吴佩然与苏佳, 甲氨蝶呤联合雷公藤多甙片的应用于治疗类风湿关节炎的治疗效果观察. 医学美学美容, 2018. 27(13): 第29页.

[19]. 唐佳, 甲氨蝶呤联合雷公藤多甙片在类风湿性关节炎治疗中的效果观察. 特别健康, 2020(27): 第84-85页.

[20]. 靳广书, 甲氨蝶呤联合雷公藤多甙片治疗老年类风湿性关节炎疗效观察.

[21]. 范俊, 甲氨蝶呤联合雷公藤多甙片治疗类风湿性关节炎的疗效观察. 浙江临床医学, 2018. 20(5): 第834-835,838页.

[22]. 李爽, 甲氨蝶呤联合雷公藤多甙片治疗类风湿性关节炎的疗效及安全性评价. 中国疗养医学, 2016. 25(5): 第527-529页.

[23]. 丁丽萍与时永强, 甲氨蝶呤联合雷公藤多甙片治疗类风湿性关节炎的临床效果观察. 医药前沿, 2018. 8(16): 第73-74页.

[24]. 周辉, 甲氨蝶呤联合雷公藤多甙片治疗类风湿性关节炎的临床研究. 饮食保健, 2020. 7(7): 第49页.

[25]. 陈珊, 甲氨蝶呤联合雷公藤多甙片治疗类风湿性关节炎的有效性. 心电图杂志(电子版), 2017(04): 第57-58页.

[26]. 闫琳毅与陆利, 甲氨蝶呤联合雷公藤多甙片治疗类风湿性关节炎疗效及安全性研究. 中国现代医生, 2015. 53(8): 第90-92页.

[27]. 雷尚文等, 甲氨蝶呤联合雷公藤多苷对中老年类风湿关节炎患者的治疗效果. 中国实用医药, 2020. 15(13): 第141-143页.

[28]. 张利, 甲氨蝶呤联合雷公藤多苷片治疗老年类风湿关节炎的临床疗效及安全性分析. 中国现代药物应用, 2020. 14(24): 第216-218页.

[29]. 朱琳等, 甲氨蝶呤联合雷公藤多苷片治疗老年类风湿关节炎的临床疗效及随访分析. 世界中西医结合杂志, 2020. 15(2): 第339-343,347页.

[30]. 李燕青, 郭春连与卢家淇, 甲氨蝶呤联合雷公藤多苷片治疗类风湿性关节炎患者的疗效及不良反应分析. 内科, 2019. 14(4): 第424-426页.

[31]. 刘海燕, 甲氨蝶呤联合雷公藤多苷治疗类风湿关节炎的临床效果观察. 临床合理用药杂志, 2019. 12(2): 第13-14页.

[32]. 王雪凤与栾照家, 甲氨蝶呤联合雷公藤多苷治疗类风湿性关节炎的临床疗效观察. 国际医药卫生导报, 2015. 21(17): 第2598-2600页.

[33]. 高登文, 甲氨蝶呤联合小剂量来氟米特、雷公藤多甙片治疗类风湿性关节炎的疗效及安全性. 泰山医学院学报, 2017. 38(5): 第524-525页.

[34]. 董霞等, 老年类风湿关节炎治疗中雷公藤甲素的应用价值. 中国保健营养（下旬刊）, 2013. 23(7): 第3909-3910页.

[35]. 邓媛等, 雷公藤对类风湿关节炎疗效及IgA、IgG、RF变化研究. 中华中医药学刊, 2020. 38(2): 第234-236页.

[36]. 张文等. 雷公藤多甙联合甲氨蝶呤治疗类风湿关节炎的安全性及有效性研究. in 第15次全国风湿病学学术会议. 2010. 西安.

[37]. 冯江江, 雷公藤多甙联合甲氨蝶呤治疗类风湿关节炎的疗效分析. 中西医结合心血管病电子杂志, 2019. 7(25): 第194+196页.

[38]. 李广科, 雷公藤多甙联合甲氨蝶呤治疗类风湿关节炎的疗效研究. 齐齐哈尔医学院学报, 2017. 38(17): 第2007-2008页.

[39]. 周飞等, 雷公藤多甙联合甲氨蝶呤治疗类风湿关节炎的临床疗效及机制探讨. 中南医学科学杂志, 2018. 46(3): 第257-259,266页.

[40]. 杨春燕, 李梦霞与徐笑笑, 雷公藤多甙联合甲氨蝶呤治疗类风湿关节炎患者的临床效果观察. 临床合理用药杂志, 2019. 12(11): 第69-70页.

[41]. 张晓利与谭静, 雷公藤多甙联合甲氨蝶呤治疗类风湿关节炎临床观察. 中国医药导刊, 2012(z2): 第602-603页.

[42]. 陆栋, 雷公藤多甙联合甲氨蝶呤治疗类风湿性关节炎的疗效及安全性观察. 中国医学工程, 2016. 24(11): 第96-97页.

[43]. 赵法来与刘士同, 雷公藤多甙联合甲氨蝶呤治疗类风湿性关节炎临床疗效观察. 首都食品与医药, 2020. 27(7): 第83页.

[44]. 李凌汉, 麦培根与陈宝红, 雷公藤多甙联合免疫抑制剂治疗类风湿关节炎疗效及对炎性因子的影响. 现代中西医结合杂志, 2017. 26(10): 第1088-1090页.

[45]. 于得泓, 战艺与石红, 雷公藤多甙片联合甲氨喋呤治疗对类风湿关节炎患者红细胞沉降率、C反应蛋白及类风湿因子的影响. 中国医药科学, 2019. 9(16): 第90-92,157页.

[46]. 庄铭城, 王小燕与陈培嘉, 雷公藤多甙片联合甲氨喋呤治疗类风湿关节炎的临床疗效及对炎症因子的影响分析. 中国医药科学, 2018. 8(18): 第46-49页.

[47]. 徐光平, 徐海燕与于久秀, 雷公藤多甙片联合甲氨蝶呤治疗31例类风湿性关节炎患者的临床疗效观察. 中国农村卫生, 2017(18): 第93-94页.

[48]. 侯宏理与马琳琳, 雷公藤多甙片联合甲氨蝶呤治疗活动性类风湿关节炎的临床研究. 内蒙古中医药, 2017. 36(17): 第46页.

[49]. 侯宏理与马琳琳, 雷公藤多甙片联合甲氨蝶呤治疗活动性类风湿性关节炎的临床研究. 内蒙古中医药, 2017. 36(17): 第43页.

[50]. 谷敬欣等, 雷公藤多甙片联合甲氨蝶呤治疗类风湿性关节炎的疗效观察. 健康大视野, 2020(1): 第68-69页.

[51]. 王淼, 雷公藤多甙片联合甲氨蝶呤治疗类风湿性关节炎临床研究. 医药论坛杂志, 2016(7): 第144-145页.

[52]. 陈芍等, 雷公藤多甙片与甲氨喋呤联合治疗类风湿关节炎的临床疗效及对炎症因子的影响. 现代生物医学进展, 2017. 17(4): 第713-716页.

[53]. 刘艳秋, 雷公藤多甙片与甲氨蝶呤联合应用方案治疗类风湿性 关节炎的临床评估. 中国伤残医学, 2019. 27(7): 第68-70页.

[54]. 谢春花, 雷公藤多苷+甲氨蝶呤治疗类风湿性关节炎的效果及不良反应发生率影响评价. 临床医药文献电子杂志, 2019. 6(80): 第164+171页.

[55]. 李媛, 雷公藤多苷和甲氨蝶呤联合治疗类风湿性关节炎的临床研究. 中国处方药, 2019. 17(11): 第107-109页.

[56]. 刘翠莲与蔡文虹, 雷公藤多苷联合甲氨蝶呤对老年类风湿关节炎患者血清中细胞因子水平的影响. 中国处方药, 2019. 17(6): 第93-94页.

[57]. 王德志, 雷公藤多苷联合甲氨蝶呤治疗类风湿关节炎的疗效观察. 中国保健营养, 2017. 27(22): 第315-316页.

[58]. 刘君勇与罗莉容, 雷公藤多苷联合甲氨蝶呤治疗类风湿关节炎的临床价值研究. 中国处方药, 2019. 17(3): 第67-68页.

[59]. 王银娥, 雷公藤多苷联合甲氨蝶呤治疗类风湿关节炎的临床价值研究. 健康必读, 2018(22): 第51-52页.

[60]. 毕丹艳等, 雷公藤多苷联合甲氨蝶呤治疗类风湿关节炎的临床疗效研究. 中国临床药理学杂志, 2016. 32(10): 第880-882页.

[61]. 马喜喜, 雷公藤多苷联合甲氨蝶呤治疗类风湿关节炎的临床疗效研究. 健康之路, 2018. 17(10): 第29页.

[62]. 陈鹏等, 雷公藤多苷联合甲氨蝶呤治疗类风湿关节炎的随机对照试验. 安徽中医学院学报, 2011. 30(6): 第28-32页.

[63]. 张卫华等, 雷公藤多苷联合甲氨蝶呤治疗类风湿关节炎的效果分析. 临床医学, 2019. 39(6): 第92-93页.

[64]. 刘醉红, 雷公藤多苷联合甲氨蝶呤治疗类风湿关节炎的药代动力学研究. 健康之路, 2015. 14(10): 第24页.

[65]. 王在红, 杨金玲与王露, 雷公藤多苷联合甲氨蝶呤治疗类风湿关节炎患者的效果. 中国民康医学, 2020. 32(20): 第74-76页.

[66]. 陈曾凤等, 雷公藤多苷联合甲氨蝶呤治疗类风湿关节炎活动期患者的疗效及对血清CD62p、CD41的影响. 现代生物医学进展, 2018. 18(20): 第3909-3912,3921页.

[67]. 袁毅, 雷公藤多苷联合甲氨蝶呤治疗类风湿关节炎效果观察. 临床医学, 2018. 38(1): 第100-101页.

[68]. 高鹏, 霍爱鑫与刘宇宏, 雷公藤多苷联合甲氨蝶呤治疗类风湿性关节炎的疗效. 西部医学, 2017. 29(11): 第1511-1515页.

[69]. 田金菊与俞襄玲, 雷公藤多苷联合甲氨蝶呤治疗类风湿性关节炎的疗效. 名医, 2019(06): 第231页.

[70]. 李雪娇, 雷公藤多苷联合甲氨蝶呤治疗类风湿性关节炎的疗效. 糖尿病天地, 2020. 17(9): 第90,93页.

[71]. 陈媛清, 雷公藤多苷联合甲氨蝶呤治疗类风湿性关节炎的疗效观察. 中国保健营养, 2018. 28(4): 第74-75页.

[72]. 王露, 雷公藤多苷联合甲氨蝶呤治疗类风湿性关节炎的临床疗效. 中国实用医刊, 2018. 45(10): 第117-119页.

[73]. 张文娟, 雷公藤多苷联合甲氨蝶呤治疗类风湿性关节炎短期疗效及对患者外周血炎性因子的影响. 世界最新医学信息文摘, 2019(51): 第129-130页.

[74]. 王胜男等, 雷公藤多苷联合甲氨蝶呤治疗类风湿性关节炎短期疗效及对外周血IL-6、IL-10和TNF-α水平的影响. 中国生化药物杂志, 2017. 37(9): 第202-204页.

[75]. 沈杰与张之澧. 雷公藤多苷联合小剂量甲氨蝶呤治疗老年性类风湿关节炎临床观察. in 第四次全国雷公藤学术会议. 2004. 中国上海.

[76]. 姚晓颖, 潘昉与周杰, 雷公藤多苷片辅助治疗类风湿关节炎的临床分析. 心理医生, 2018. 24(24): 第107-108页.

[77]. 王海波, 崔永虹与刘杰, 雷公藤多苷片联合甲氨蝶呤片治疗类风湿性关节炎临床分析. 临床合理用药杂志, 2016. 9(35): 第52-53页.

[78]. 王亚芳, 宋爱凤与王梅, 雷公藤多苷片联合甲氨蝶呤治疗类风湿性关节炎的疗效. 中华养生保健, 2020. 38(9): 第56-58页.

[79]. 王慧娟, 雷公藤多苷片联合甲氨蝶呤治疗类风湿性关节炎的临床价值分析. 四川解剖学杂志, 2018(4): 第92-93页.

[80]. 高飞等, 雷公藤多苷片联合甲氨蝶呤治疗类风湿性关节炎的临床价值观察. 健康大视野, 2019(6): 第34页.

[81]. 龙洁等, 雷公藤多苷片联合甲氨蝶呤治疗类风湿性关节炎的效果. 中国医药导报, 2019. 16(7): 第71-75页.

[82]. 龙朝阳等, 雷公藤多苷片治疗类风湿关节炎老年患者的临床疗效. 内蒙古中医药, 2019. 38(09): 第72-73页.

[83]. 王翰洲等, 雷公藤多苷片治疗类风湿关节炎作用于IL-23/IL-17轴相关细胞因子的系统评价和meta分析. 中国医药导报, 2020. 17(12): 第128-132,160页.

[84]. 郑冰, 雷公藤多苷与甲氨蝶呤联合治疗类风湿关节炎临床疗效和安全性分析. 医药前沿, 2017. 7(15): 第159-160页.

[85]. 李松伟等, 雷公藤多苷治疗类风湿关节炎肺间质病变的临床研究. 中华中医药学刊, 2017. 35(7): 第1662-1664页.

[86]. 应振华, 雷公藤合MTX治疗类风湿性关节炎28例疗效初步观察. 浙江中西医结合杂志, 1997. 7(6): 第352-353页.

[87]. 张鸽, 雷公藤甲素治疗老年类风湿关节炎临床治疗观察. 家庭医药·就医选药, 2020(2): 第77页.

[88]. 王萍, 雷公藤联合甲氨蝶呤治疗类风湿性关节炎的效果比较. 中国医药指南, 2019. 17(5): 第2-3页.

[89]. 于守杰与贾倩, 雷公藤片联合甲氨蝶呤对类风湿关节炎的疗效观察. 基层医学论坛, 2020. 24(22): 第3155-3157页.

[90]. 何小宇等, 雷公藤片治疗类风湿关节炎的疗效分析. 慢性病学杂志, 2017(5): 第575-576页.

[91]. 王晋英, 雷公藤治疗类风湿关节炎的疗效. 健康之路, 2018. 17(10): 第318页.

[92]. 陈锦然, 类风湿性关节炎采用雷公藤多苷与甲氨蝶呤联合治疗的效果. 实用中西医结合临床, 2019. 19(07): 第145-146页.

[93]. 李婧婷, 类风湿性关节炎患者予以甲氨喋呤、雷公藤多甙片联合用药疗效研究. 中国保健营养, 2017. 27(24): 第240-241页.

[94]. 高健, 联合使用雷公藤、甲氨蝶呤对类风湿关节炎(RA)的疗效观察. 世界最新医学信息文摘, 2018. 18(A0): 第103-104页.

[95]. 赵伟伟, 评价雷公藤多苷联合甲氨蝶呤治疗类风湿关节炎的疗效及安全性. 首都食品与医药, 2018. 25(22): 第38页.

[96]. 翟相举, 探究甲氨蝶呤联合雷公藤多甙片应用于类风湿关节炎的治疗效果. 中国实用医药, 2019. 14(21): 第107-108页.

[97]. 刘亚婧等, 探讨雷公藤多苷与甲氨蝶呤联合治疗类风湿性关节炎(RA)的近期疗效及安全性. 健康大视野, 2019(19): 第70页.

[98]. 陈士军, 朱卫民与田培军, 小剂量雷公藤总苷联合甲氨蝶呤对类风湿关节炎患者临床症状、炎性因子及关节功能的影响. 临床误诊误治, 2020. 33(3): 第52-56页.

[99]. 孙鹏丽等, 自拟蠲痹汤加味联合甲氨蝶呤治疗类风湿性关节炎疗效观察及护理. 中外健康文摘, 2011. 08(1): 第350-351页.

[100]. 王淼, 雷公藤多甙片联合甲氨蝶呤治疗类风湿性关节炎临床研究. 医药论坛杂志, 2016(7): 第144-145页.

[101]. 陈芍等, 雷公藤多甙片与甲氨喋呤联合治疗类风湿关节炎的临床疗效及对炎症因子的影响. 现代生物医学进展, 2017. 17(4): 第713-716页.

**91 irrelevant studies**

[1]. 刘瑜与陈瑾, 26例成人Still病回顾性分析. 皮肤性病诊疗学杂志, 2019. 26(1): 第12-15页.

[2]. 杨爱华与李强, 28例类风湿关节炎的治疗分析. 医药前沿, 2013(26): 第198-198页.

[3]. 吴庆军等, 5例抗丙氨酰 tRNA 合成酶抗体阳性患者临床特征. 中华临床免疫和变态反应杂志, 2014(2): 第129-133页.

[4]. Grzegorzewska, A.E., et al., Disseminated cutaneous Kaposi sarcoma in a patient receiving triptolide/tripdiolide for rheumatoid arthritis. Medical Science Monitor, 2012. 18(8): p. CS67-CS71.

[5]. Li, X., Y. Jia and Z. Li, Status of rheumatology practice and professional training courses in rural areas of China—an ILAR project. Clinical Rheumatology, 2017. 36(1): p. 213-216.

[6]. 赖晋智与方理刚, 第42例：临床表现心悸、胸闷、不能平卧. 中国心血管杂志, 2015(4): 第299-300页.

[7]. 罗家昂等, 多中心网状组织细胞增生症合并类风湿性关节炎1例. 疑难病杂志, 2017. 16(4): 第407-408页.

[8]. 赵梅花等, 风湿性多肌痛2例并文献复习. 海南医学, 2019. 30(7): 第892-895页.

[9]. 郭春霞, 齐静与赵敏, 过量服用甲氨蝶呤致严重口腔溃疡并发真菌感染1例. 华北煤炭医学院学报, 2006. 8(1): 第3页.

[10]. 徐婧, 石连杰与张学武, 肌内注射甲氨蝶呤治疗类风湿关节炎合并短肠综合征1例. 中华医学杂志, 2017. 97(17): 第1352-1353页.

[11]. 徐婧, 石连杰与张学武, 肌内注射甲氨蝶呤治疗类风湿关节炎合并短肠综合征一例. 中华医学杂志, 2017. 97(17): 第1352-1353页.

[12]. 田杨, 张永如与张淑慎, 激素冲击治疗类风湿性关节炎并发多脏器受损1例. 广东医学, 2001. 22(6): 第495页.

[13]. 朱芳晓与周润华. 甲氨蝶呤联合羟氯喹或雷公藤多苷治疗抗环瓜氨酸肽抗体阳性的早期类风湿关节炎的临床研究. in 中华医学会第十六次全国风湿病学学术会议. 2011. 长沙.

[14]. 马志敏, 邓丽丽与罗彦玲, 甲氨蝶呤致骨髓抑制1例. 中国药学杂志, 1999(03): 第50页.

[15]. 张蕊等, 甲氨蝶呤致再生障碍性贫血1例及分析. 华西医学, 2009. 24(7): 第1887-1888页.

[16]. 王培光等, 巨大型环状肉芽肿1例. 中国皮肤性病学杂志, 2008. 22(10): 第631-632页.

[17]. 王培光等, 巨大型环状肉芽肿1例. 中国皮肤性病学杂志, 2008. 22(10): 第631-632页.

[18]. 林昌松, 梁江与陈秀敏. 口服昆仙胶囊致食管溃疡1例. in 全国第十届中西医结合风湿病学术会议. 2012. 中国四川成都.

[19]. 张文等. 雷公藤多苷联合甲氨蝶呤治疗类风湿关节炎的安全性和有效性. in 中华医学会第十六次全国风湿病学学术会议. 2011. 长沙.

[20]. 闫磊, 戚务芳与李媛, 类风湿关节炎伴血管炎合并神经损害1例. 中国免疫学杂志, 2017. 33(12): 第1874-1876页.

[21]. 蒲梦君等, 类风湿关节炎合并纯红细胞再生障碍性贫血一例. 中华临床医师杂志（电子版）, 2013(10): 第4639-4640页.

[22]. 蒋琼. 类风湿关节炎合并骨筋膜室综合征1例. in 2015年浙江省风湿病学学术年会. 2015. 中国浙江金华.

[23]. 郭颖, 逄崇杰与巩路, 类风湿关节炎合并重痘肌无力1例. 中华风湿病学杂志, 2012. 16(10): 第718页.

[24]. 郭颖, 逄崇杰与巩路, 类风湿关节炎合并重症肌无力一例. 中华风湿病学杂志, 2012(10): 第718页.

[25]. 林永与袁会兰, 类风湿关节炎与主动脉夹层并存一例报告. 临床误诊误治, 2010. 23(S2): 第62-63页.

[26]. 朱辉军等, 类风湿性关节炎并血管炎1例. 临床医学, 2009. 29(12): 第110页.

[27]. 邬丽娜等, 良性溃疡并发胃结肠瘘1例. 中华消化内镜杂志, 2010(7): 第385-386页.

[28]. 邬丽娜等, 良性溃疡并发胃结肠瘘一例. 中华消化内镜杂志, 2010(07): 第385-386页.

[29]. 王安琪等, 刘喜德温清并用治疗类风湿关节炎医案3则. 新中医, 2017. 49(12): 第191-192页.

[30]. 王文琴. 硫酸羟氯喹致葡萄膜炎2例. in 2012年浙江省风湿病学学术年会. 2012. 中国浙江嘉兴.

[31]. 张会敏, 张迪展与周桂芝, 毛囊黏蛋白病一例. 实用皮肤病学杂志, 2016. 9(6): 第408-409页.

[32]. 李迎伟等, 强直性脊柱炎合并急性髓细胞白血病2例. 临床荟萃, 2012. 27(20): 第1818-1819页.

[33]. 蒋昕钰与殷凯生, 沙美特罗替卡松粉吸入剂致关节痛1例. 药物流行病学杂志, 2015(10): 第633-634页.

[34]. 刘岳, 王芳与黄慈波, 嗜酸性粒细胞增多症并高滴度类风湿因子的类风湿关节炎1例. 中华风湿病学杂志, 2015. 19(7): 第483-484,插2页.

[35]. 刘岳, 王芳与黄慈波, 嗜酸性粒细胞增多症并高滴度类风湿因子的类风湿关节炎一例. 中华风湿病学杂志, 2015. 19(07): 第483-484+506页.

[36]. 张铎, 张莉与戴军有, 糖尿病伴白塞病肾损害1例. 中国中西医结合肾病杂志, 2014(6): 第545-545页.

[37]. 郭云柯, 陆燕与纪伟, 误诊为干燥综合征的 IgG4相关性米库列兹病1例郭云柯，陆燕，纪伟. 广东医学, 2015(1): 第26-26页.

[38]. 马喜喜等, 依那西普治疗炎性关节病无效转换为英夫利西单抗后有效二例. 中华临床医师杂志(电子版), 2014. 8(09): 第1800-1802页.

[39]. 顾镭等, 重组人Ⅱ型肿瘤坏死因子受体-抗体融合蛋白治疗类风湿关节炎出现巩膜炎1例. 中华风湿病学杂志, 2009(10): 第723-724,插2页.

[40]. 顾镭等, 重组人Ⅱ型肿瘤坏死因子受体-抗体融合蛋白治疗类风湿关节炎出现巩膜炎一例. 中华风湿病学杂志, 2009(10): 第723-724+730页.

[41]. 徐惠萍, 齐文成与戚务芳, 重组人肿瘤坏死因子受体-抗体融合蛋白治疗难治性成人斯蒂尔病1例. 中华内科杂志, 2010. 49(12): 第1063-1064页.

[42]. 吕倩雯, 比较雷公藤和甲氨蝶呤治疗类风湿关节炎的有效性和安全性, 2013, 中国医学科学院;清华大学医学部;北京协和医学院.

[43]. 李芯, 雷公藤治疗类风湿关节炎的疗效及安全性评估, 2012, 北京协和医学院.

[44]. 范天宇与刘学理, 2010－2011年某三甲医院治疗类风湿关节炎药物使用分析. 中国当代医药, 2012. 19(31): 第178-180页.

[45]. Hou, Z., et al., Leprosy mimicking connective tissue disease - A challenge for rheumatologists. International Journal of Clinical and Experimental Medicine, 2016. 9(9): p. 18040-18046.

[46]. Xu, C., et al., Societal costs of rheumatoid arthritis in China: A hospital-based cross-sectional study. Arthritis Care and Research, 2014. 66(4): p. 523-531.

[47]. 吕倩雯与张烜, 比较雷公藤多甙和甲氨蝶呤对治疗活动性类风湿关节炎的有效性与安全性. 中华内科杂志, 2015(6): 第537页.

[48]. 益西拉姆等, 从病例分析RA药物的临床应用. 西藏科技, 2017(2): 第53-56页.

[49]. 姜凤玲, 基于《患者报告的临床结局量表》对RA治疗方案的评价研究, 2020, 长春中医药大学.

[50]. 周磊等, 甲氨蝶呤治疗类风湿性关节炎的应用及最佳剂量研究. 心理月刊, 2020. 15(08): 第169页.

[51]. 健康观察. 健康管理, 2014(05): 第5-9页.

[52]. 龚小东, 雷公藤多苷和甲氨蝶呤联合治疗类风湿性关节炎效果如何? 特别健康, 2020(27): 第245页.

[53]. 赵旭颖等, 雷公藤多苷联合硫酸羟氯喹治疗类风湿关节炎致急性粒细胞缺乏后的治疗探讨. 中国医刊, 2020. 55(10): 第1135-1138页.

[54]. 赵钟文等. 雷公藤合剂配合手关节操治疗活动期RA临床研究. in 全国第十二届中西医结合风湿病学术会议. 2014. 中国天津.

[55]. 蒋旭, 类风湿关节炎相关性间质性肺疾病危险因素及临床特征研究, 2020, 郑州大学.

[56]. 苏暄与陈明雁, 临床研究如何曲折前进?——访雷公藤多甙比较甲氨蝶呤临床试验主要设计者张烜教授. 中国医药科学, 2014. 4(8): 第4-6页.

[57]. 张楠与王剑, 母女先后患带状疱疹和水痘. 兵团医学, 2015. 46(4): 第封3-封4页.

[58]. 王栩, 我院2012－2014年口服抗类风湿性关节炎药物使用调查分析. 中国乡村医药, 2016. 23(7): 第44-46页.

[59]. 黄建林, 有类风关,怀孕用药有讲究. 家庭医生, 2017(1): 第11页.

[60]. 陈兴等, 治疗类风湿性关节炎的民间偏方. 世界最新医学信息文摘, 2015. 15(56): 第6-8页.

[61]. 中成药或将改变类风湿治疗策略. 河北中医, 2014. 36(4): 第564页.

[62]. 韩曼, 中国类风湿关节炎患者报告的临床结局量表优化及应用研究, 2017, 中国中医科学院.

[63]. Huang, Y., et al., Drug-herb interactions between commonly-used drugs and traditional Chinese medicine herbs in rheumatoid arthritis: A systematic review. International Journal of Rheumatic Diseases, 2015. 18: p. 99.

[64]. Sun, Y., et al., Effect of downregulation of serum MMP-3 levels by traditional Chinese medicine ingredients combined with methotrexate on the progression of bone injury in patients with rheumatoid arthritis: A protocol for a systematic review and meta-analysis. Medicine (Baltimore), 2020. 99(43): p. e22841.

[65]. Liu, Y., et al., Extracts of Tripterygium wilfordii hook F in the treatment of rheumatoid arthritis: A systemic review and meta-analysis of randomised controlled trials. Evidence-based Complementary and Alternative Medicine, 2013. 2013.

[66]. Yin, C., et al., Meta-analysis of tripterygium glycosides combined with methotrexate in the treatment of rheumatoid arthritis. Chinese Journal of Tissue Engineering Research, 2019. 23(35): p. 5710-5717.

[67]. Zhou, Y.Y., et al., The effectiveness and safety of Tripterygium wilfordii Hook. F extracts in rheumatoid arthritis: A systematic review and meta-analysis. Frontiers in Pharmacology, 2018. 9(APR).

[68]. Wang, H.L., et al., Tripterygium wilfordii Hook F versus conventional synthetic disease-modifying anti-rheumatic drugs as monotherapy for rheumatoid arthritis: A systematic review and network meta-analysis. BMC Complementary and Alternative Medicine, 2016. 16(1).

[69]. Lv, Q.W., et al., TwHF versus methotrexate in the treatment of rheumatoid arthritis: response to Landewe's comment on the TRIFRA study. Ann Rheum Dis, 2014. 73(10): p. e63.

[70]. 黄丽军等, 蜂针治疗类风湿关节炎Meta分析. 中华中医药学刊, 2017. 35(5): 第1211-1215页.

[71]. 赵文甲, 刘维与吴沅皞, 桂枝芍药知母汤治疗类风湿关节炎疗效及安全性的Meta分析. 中国老年学杂志, 2018. 38(18): 第4435-4440页.

[72]. 王晓月等, 雷公藤多苷(甙)片单用或联合甲氨蝶呤对类风湿关节炎临床表现改善作用的Meta分析. 中国中药杂志, 2019. 44(16): 第3533-3541页.

[73]. 李泰贤等, 雷公藤多苷(甙)片单用或联合甲氨蝶呤治疗类风湿关节炎实验室指标改善作用的Meta分析. 中国中药杂志, 2019. 44(16): 第3542-3550页.

[74]. 陈文佳等, 雷公藤多苷(甙)片单用或联用甲氨蝶呤治疗类风湿关节炎临床疗效RCT研究的Meta分析. 中国中药杂志, 2020. 45(04): 第791-797页.

[75]. 杨俊等, 雷公藤多苷(甙)片对类风湿关节炎促炎细胞因子影响的系统评价. 中国中药杂志, 2020. 45(04): 第764-774页.

[76]. 李逸群等, 雷公藤多苷(甙)片治疗类风湿关节炎的安全性系统评价. 中国中药杂志, 2020. 45(04): 第775-790页.

[77]. 尹聪等, 雷公藤多苷联合甲氨蝶呤治疗类风湿关节炎的Meta分析. 中国组织工程研究, 2019. 23(35): 第5710-5717页.

[78]. 朱光昭等, 雷公藤多苷片治疗类风湿关节炎骨破坏的系统评价和Meta分析. 中国中药杂志, 2019(15): 第3358-3364页.

[79]. 王晓宇等. 雷公藤联合甲氨蝶呤治疗类风湿性关节炎的meta分析及生物信息学研究. in “农业健康与环境”组学大数据整合生物信息学研讨会. 2017. 中国内蒙古通辽.

[80]. 王晓宇, 雷公藤联合甲氨蝶呤治疗类风湿性关节炎的meta分析及生物信息学研究, 2017, 浙江中医药大学.

[81]. 王胜男, 雷公藤治疗类风湿关节炎的系统评价, 2011, 福建医科大学.

[82]. 王胜男, 雷公藤治疗类风湿性关节炎的系统评价, 2011, 福建医科大学.

[83]. 左进红, 中成药治疗类风湿性关节炎的网状Meta分析, 2018, 广州中医药大学.

[84]. 杨梅玉等, 中药治疗类风湿关节炎的有效性与安全性评价. 北京中医药, 2009. 28(7): 第497-500页.

[85]. 姜林娣, 赵耐青与倪立青, 类风湿关节炎二线药物不良反应回顾性调查. 中华流行病学杂志, 2002. 23(3): 第213-217页.

[86]. 姜林娣, 梅振武与倪立清, 类风湿关节炎二线药物用药决策分析. 中国药物与临床, 2001. 1(1): 第20-24页.

[87]. 余步云等, 类风湿关节炎疗效的追踪观察. 中国药物与临床, 2001. 1(1): 第13-15页.

[88]. 李静等, 基于文本挖掘方法探索雷公藤的用药规律. 中国中医基础医学杂志, 2013(4): 第447-448,452页.

[89]. 孟庆良等, 基于文本挖掘技术析甲氨蝶呤与中医治疗联合应用的规律. 中国中医基础医学杂志, 2014. 20(12): 第1665-1667页.

[90]. 王妍, 雷公藤与甲氨蝶呤、黄芪与当归联用治疗RA机制的生物信息学分析, 2012, 中国中医科学院.

[91]. 徐卫东等, 利用文本挖掘技术分析治疗类风湿关节炎中成药和西药使用基本规律. 辽宁中医杂志, 2012. 39(3): 第425-427页.

**16 reviews**

[1]. Baljit, K., et al., A never-ending story of rheumatoid arthritis. International Journal of Pharmacy and Pharmaceutical Sciences, 2020. 12(7): p. 10-17.

[2]. Li, Z.G., A new look at rheumatology in China - Opportunities and challenges. Nature Reviews Rheumatology, 2015. 11(5): p. 313-317.

[3]. Lu, A.P., Z.X. Bian and K.J. Chen, Bridging the traditional chinese medicine pattern classification and biomedical disease diagnosis with systems biology. Chinese Journal of Integrative Medicine, 2012. 18(12): p. 883-890.

[4]. Ho, L.J. and J.H. Lai, Chinese herbs as immunomodulators and potential disease-modifying antirheumatic drugs in autoimmune disorders. Current Drug Metabolism, 2004. 5(2): p. 181-192.

[5]. Zhang, C., et al., Clinical trials of integrative medicine for rheumatoid arthritis: Issues and recommendations. Chinese Journal of Integrative Medicine, 2015. 21(6): p. 403-407.

[6]. Rogers, G.B., Germs and joints: The contribution of the human microbiome to rheumatoid arthritis. Nature Medicine, 2015. 21(8): p. 839-841.

[7]. 刘汉顺, RA中医证型与影像学相关性的研究, 2008, 贵阳中医学院.

[8]. Viegener, U., Rheumatoid arthritis: Traditional Chinese Medicine preparation convinces in study. Pharmazeutische Zeitung, 2014. 159(18).

[9]. Uehleke, B., Rheumatoid arthritis: Tripterygium wilfordii is not inferior to methotrexate. Zeitschrift fur Phytotherapie, 2015. 35(6): p. 273-274.

[10]. Chang, C., Unmet needs in the treatment of autoimmunity: From aspirin to stem cells. Autoimmunity Reviews, 2014. 13(4-5): p. 331-346.

[11]. 巴鑫, 陈哲与涂胜豪, 从表观遗传学角度看雷公藤治疗类风湿关节炎的研究进展. 中华风湿病学杂志, 2018. 22(11): 第785-788页.

[12]. 李金全, 运乃茹与温学红, 雷公藤多苷在常见自身免疫性疾病中的临床应用及安全性. 医学综述, 2016. 22(19): 第3850-3854页.

[13]. 杨荣, 张姝与王永福, 雷公藤在常见结缔组织病中的应用研究进展. 包头医学院学报, 2015. 31(8): 第154-156页.

[14]. 李鹏与方剑乔, 雷公藤制剂治疗类风湿性关节炎的临床概况. 内蒙古中医药, 2008(11): 第70-71页.

[15]. 许海艳等, 类风湿性关节炎治疗方法的研究进展. 南昌大学学报(医学版), 2020. 60(05): 第97-102页.

[16]. 刘星, 中西医治疗类风湿性关节炎研究进展. 解放军医药杂志, 2012. 24(12): 第52-55页.

**7 chinese and english repeated**

[1]. Wu, Y.J., Z.Y. Lao and Z.L. Zhang, [Clinical observation on small doses Tripterygium wilfordii polyglycoside combined with methotrexate in treating rheumatoid arthritis]. Zhongguo Zhong Xi Yi Jie He Za Zhi, 2001. 21(12): p. 895-6.

[2]. Wang, X.Y., et al., [Clinical symptoms effect of Tripterygium Glycosides Tablets alone or combined with methotrexate in treatment of rheumatoid arthritis: a Meta-analysis]. Zhongguo Zhong Yao Za Zhi, 2019. 44(16): p. 3533-3541.

[3]. Lü, A.P., et al., [Correlations of clinical symptoms and treatment efficacy in patients with rheumatoid arthritis treated with Chinese herbal drugs or Western medicine]. Zhong Xi Yi Jie He Xue Bao, 2005. 3(6): p. 432-7.

[4]. Li, T.X., et al., [Meta-analysis of laboratory index of Tripterygium Glycosides Tablets in treatment of rheumatoid arthritis]. Zhongguo Zhong Yao Za Zhi, 2019. 44(16): p. 3542-3550.

[5]. Chen, W.J., et al., [Meta-analysis of RCT studies on clinical efficacy of single administration of Tripterygium Glycosides Tablets or combined administration with methotrexate against rheumatoid arthritis]. Zhongguo Zhong Yao Za Zhi, 2020. 45(4): p. 791-797.

[6]. Jiang, L., N. Zhao and L. Ni, [Retrospective study of adverse events in patients with rheumatoid arthritis treated with second-line drugs]. Zhonghua Liu Xing Bing Xue Za Zhi, 2002. 23(3): p. 213-7.

[7]. Yang, J., et al., [Systematic reviews of effects of Tripterygium Glycosides Tablets on pro-inflammatory factors in rheumatoid arthritis]. Zhongguo Zhong Yao Za Zhi, 2020. 45(4): p. 764-774.

**7 non-RA**

[1]. Chou, C.T., Alternative therapies: What role do they have in the management of lupus? Lupus, 2010. 19(12): p. 1425-1429.

[2]. 许建民等, 风湿性多肌痛住院患者38例临床诊治分析. 中华老年多器官疾病杂志, 2012. 11(12): 第893-896页.

[3]. 许杰州等, 缓作用药在强直性脊柱炎治疗中的地位. 山西医药杂志, 2000. 29(1): 第22-24页.

[4]. 李松伟等. 雷公藤多甙治疗类风湿关节炎肺间质病变的临床研究. in 2016年河南省风湿病学学术年会. 2016. 河南开封.

[5]. 高利霞, 雷公藤多苷联合甲氨蝶呤片治疗类风湿关节炎肺间质病变效果观察. 白求恩医学杂志, 2020. 18(2): 第143-145页.

[6]. 贾宁, 原发性干燥综合征合并非霍奇金淋巴瘤, 2008, 中国协和医科大学.

[7]. 梁善皓, 中医药治疗强直性脊柱炎的系统评价及用药分析, 2013, 南方医科大学.
