# Supplementary material for: Effects and Safety of the Tripterygium Glycoside Adjuvant Methotrexate Therapy in Rheumatoid Arthritis: A Systematic Review and Meta-Analysis
Source: Evid Based Complement Alternat Med. 2022 Mar 24;2022:1251478. doi: 10.1155/2022/1251478 (PMC8970871; doi:10.1155/2022/1251478)
Supplement: Supplementary Materials — Supplementary 1. Supplementary Information 1: items regarding the PRISMA checklist for network meta-analysis. Supplementary Information 2: detailed search strategies. Supplementary Information 3: a list of all excluded papers. Supplementary 2Supplementary Information 4: quality assessment using the GRADE approach. Supplementary Figure 1: forest plots for the secondary outcomes of TG adjuvant MTX therapy. Supplementary Figure 2: forest plots for the secondary outcomes of a three-month course of TG adjuvant MTX therapy at a dose of 30 mg/day. Supplementary Figure 3: forest plots for the secondary outcomes of the different courses and doses of TG adjuvant MTX therapy. Supplementary Figure 4: forest plots for the safety of TG adjuvant MTX therapy. Supplementary Figure 5: forest plots for the safety of a three-month course of TG adjuvant MTX therapy at the dose of 30 mg/day. Supplementary Figure 6: forest plots for the safety of the different courses and doses of TG adjuvant MTX therapy. [file 1251478.f1.zip › 1251478.f1/Supplementary Information 4 (2).docx]

**Supplementary Information 4: Quality assessment using the GRADE approach.**

| **Outcome** | **Participants (studies) Follow-up** | **Risk of bias** | **Inconsistency** | **Indirectness** | **Imprecision** | **Publication bias** | **Overall quality of evidence** | **Relative effect** (95% CI) |
| --- | --- | --- | --- | --- | --- | --- | --- | --- |
|  |  |  |  |  |  |  |  |  |
|  |  |  |  |  |  |  |  |  |
| **Primary outcomes** | | | | | | | | |
| **ACR20 - Course of treatment-3 months** | 406 (4 RCTs) | serious^a^ | not serious | not serious | serious^b^ | undetected | ⊕⊕㊀㊀  LOW | **RR 0.99**  (0.87 to 1.12) |
| **ACR20 - Course of treatment-6 months** | 456 (4 studies) | serious^a^ | not serious | not serious | serious^b^ | undetected | ⊕⊕㊀㊀  LOW | **RR 1.27**  (1.13 to 1.42) |
| **ACR50 - Course of treatment-3 months** | 268 (3 studies) | serious^a^ | not serious | not serious | serious^b^ | undetected | ⊕⊕㊀㊀  LOW | **RR 1.1**  (0.89 to 1.35) |
| **ACR50 - Course of treatment-6 months** | 456 (4 studies) | serious^a^ | not serious | not serious | serious^b^ | undetected | ⊕⊕㊀㊀  LOW | **RR 1.41**  (1.2 to 1.65) |
| **ACR70 - Course of treatment-6 months** | 376 (3 studies) | serious^a^ | not serious | not serious | serious^b^ | undetected | ⊕⊕㊀㊀  LOW | **RR 1.65**  (1.18 to 2.31) |
| **Secondary outcomes** | | | | | | | | |
| **SJC - Course of treatment-3 months** | 436 (5 studies) | serious^a^ | not serious | not serious | serious^b^ | undetected | ⊕⊕㊀㊀  LOW | **MD -4.13**  (-4.79 to 3.47) |
| **SJC - Course of treatment-6 months** | 456 (4 studies) | serious^a^ | not serious | not serious | serious^b^ | undetected | ⊕⊕㊀㊀  LOW | **MD -1.71**  (-2.24 to 1.17) |
| **TJC - Course of treatment-3 months** | 436 (5 studies) | serious^a^ | not serious | not serious | serious^b^ | undetected | ⊕⊕㊀㊀  LOW | **MD -3.10**  (-3.85 to 2.35) |
| **TJC - Course of treatment-6 months** | 456 (4 studies) | serious^a^ | not serious | not serious | serious^b^ | undetected | ⊕⊕㊀㊀  LOW | **MD -2.20**  (-2.90 to 1.51) |
| **ESR - Course of treatment-3 months** | 436 (5 studies) | serious^a^ | not serious | not serious | serious^b^ | undetected | ⊕⊕㊀㊀  LOW | **MD -24.18**  (-25.82 to 22.54) |
| **ESR - Course of treatment-6 months** | 456 (4 studies) | serious^a^ | not serious | not serious | serious^b^ | undetected | ⊕⊕㊀㊀  LOW | **MD -13.44**  (-14.93 to 11.95) |
| **CRP - Course of treatment-3 months** | 436 (5 studies) | serious^a^ | not serious | not serious | serious^b^ | undetected | ⊕⊕㊀㊀  LOW | **MD -1.05**  (-1.26 to 0.84) |
| **CRP - Course of treatment-6 months** | 456 (4 studies) | serious^a^ | not serious | not serious | serious^b^ | undetected | ⊕⊕㊀㊀  LOW | **MD -0.64**  (-0.84 to 0.45) |
| **RF - Course of treatment-3 months** | 238 (2 studies) | serious^a^ | not serious | not serious | serious^b^ | undetected | ⊕⊕㊀㊀  LOW | **MD -16.98**  (-25.94 to -8.02) |
| **RF - Course of treatment-6 months** | 180 (2 studies) | serious^a^ | not serious | not serious | serious^b^ | undetected | ⊕⊕㊀㊀  LOW | **MD -64.04**  (-68.72 to 59.36) |
| **Adverse effects** | | | | | | | | |
| **3 months+30 mg/d** | 504 (1 study) | serious^a^ | not serious | not serious | serious^b^ | undetected | ⊕⊕㊀㊀  LOW | **RR 0.58**  (0.22 to 1.53) |
| **3 months+60 mg/d** | 336 (2 studies) | serious^a^ | not serious | not serious | serious^b^ | undetected | ⊕⊕㊀㊀  LOW | **RR 2.73**  (0.67 to 11.21) |
| **6 months+30 mg/d** | 820 (2 studies) | serious^a^ | not serious | not serious | serious^b^ | undetected | ⊕⊕㊀㊀  LOW | **RR 1.05**  (0.58 to 1.89) |
| **6 months+60 mg/d** | 966 (2 studies) | serious^a^ | not serious | not serious | serious^b^ | undetected | ⊕⊕㊀㊀  LOW | **RR 0.87**  (0.69 to 1.11) |
| **CI:** Confidence interval; **RR:** Risk ratio. | | | | | | | | |
| **GRADE Working Group grades of evidence**  **High quality:** Further research is very unlikely to change our confidence in the estimate of effect.  **Moderate quality:** Further research is likely to have an important impact on our confidence in the estimate of effect and may change the estimate.  **Low quality:** Further research is very likely to have an important impact on our confidence in the estimate of effect and is likely to change the estimate.  **Very low quality:** We are very uncertain about the estimate. | | | | | | | | |
| **Explanation:**   1. Downgraded one level for part of unclear or high risk of bias. 2. Downgraded one level for imprecision: very small size or too wide 95% CI. | | | | | | | | |

p
