# Supplementary figures and images for: Effects and Safety of the Tripterygium Glycoside Adjuvant Methotrexate Therapy in Rheumatoid Arthritis: A Systematic Review and Meta-Analysis
Source: Evid Based Complement Alternat Med. 2022 Mar 24;2022:1251478. doi: 10.1155/2022/1251478 (PMC8970871; doi:10.1155/2022/1251478)

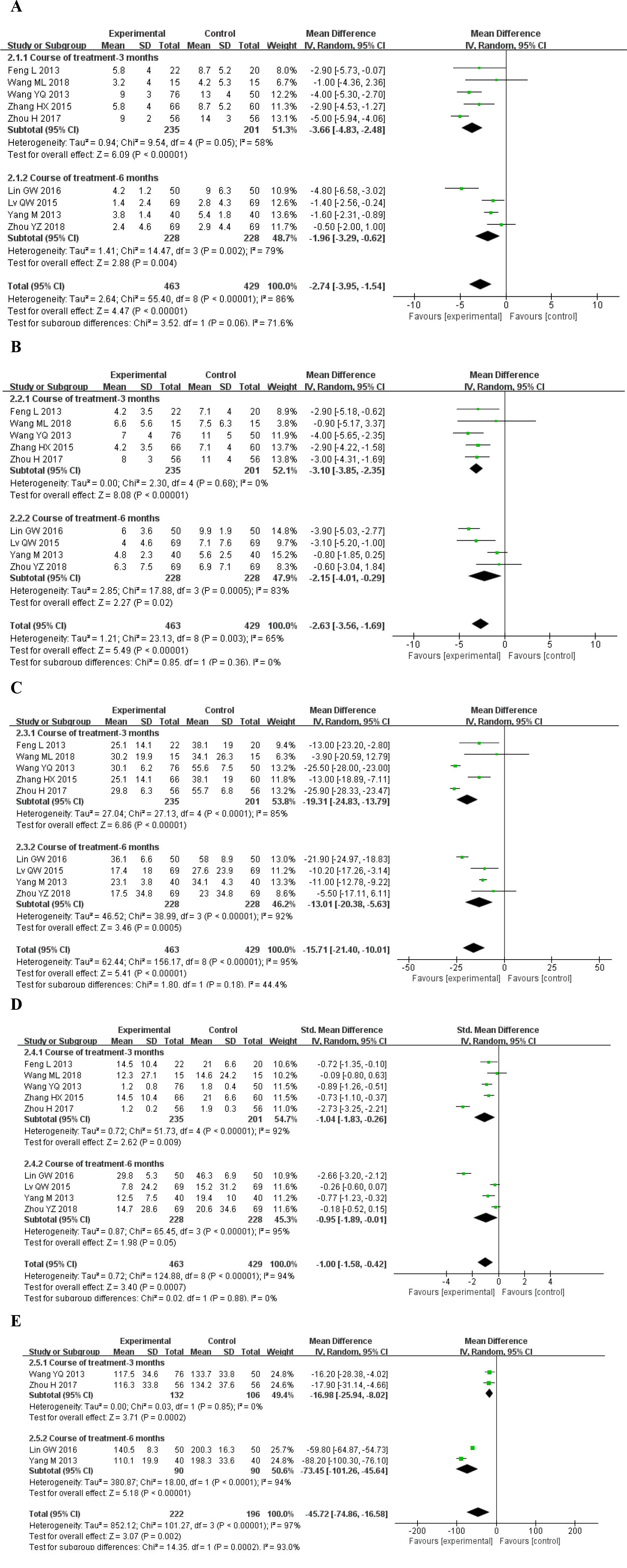

Supplement: Supplementary Materials — Supplementary 1. Supplementary Information 1: items regarding the PRISMA checklist for network meta-analysis. Supplementary Information 2: detailed search strategies. Supplementary Information 3: a list of all excluded papers. Supplementary 2Supplementary Information 4: quality assessment using the GRADE approach. Supplementary Figure 1: forest plots for the secondary outcomes of TG adjuvant MTX therapy. Supplementary Figure 2: forest plots for the secondary outcomes of a three-month course of TG adjuvant MTX therapy at a dose of 30 mg/day. Supplementary Figure 3: forest plots for the secondary outcomes of the different courses and doses of TG adjuvant MTX therapy. Supplementary Figure 4: forest plots for the safety of TG adjuvant MTX therapy. Supplementary Figure 5: forest plots for the safety of a three-month course of TG adjuvant MTX therapy at the dose of 30 mg/day. Supplementary Figure 6: forest plots for the safety of the different courses and doses of TG adjuvant MTX therapy. [file 1251478.f1.zip › 1251478.f1/Supplement Fig 1 (1).jpg]

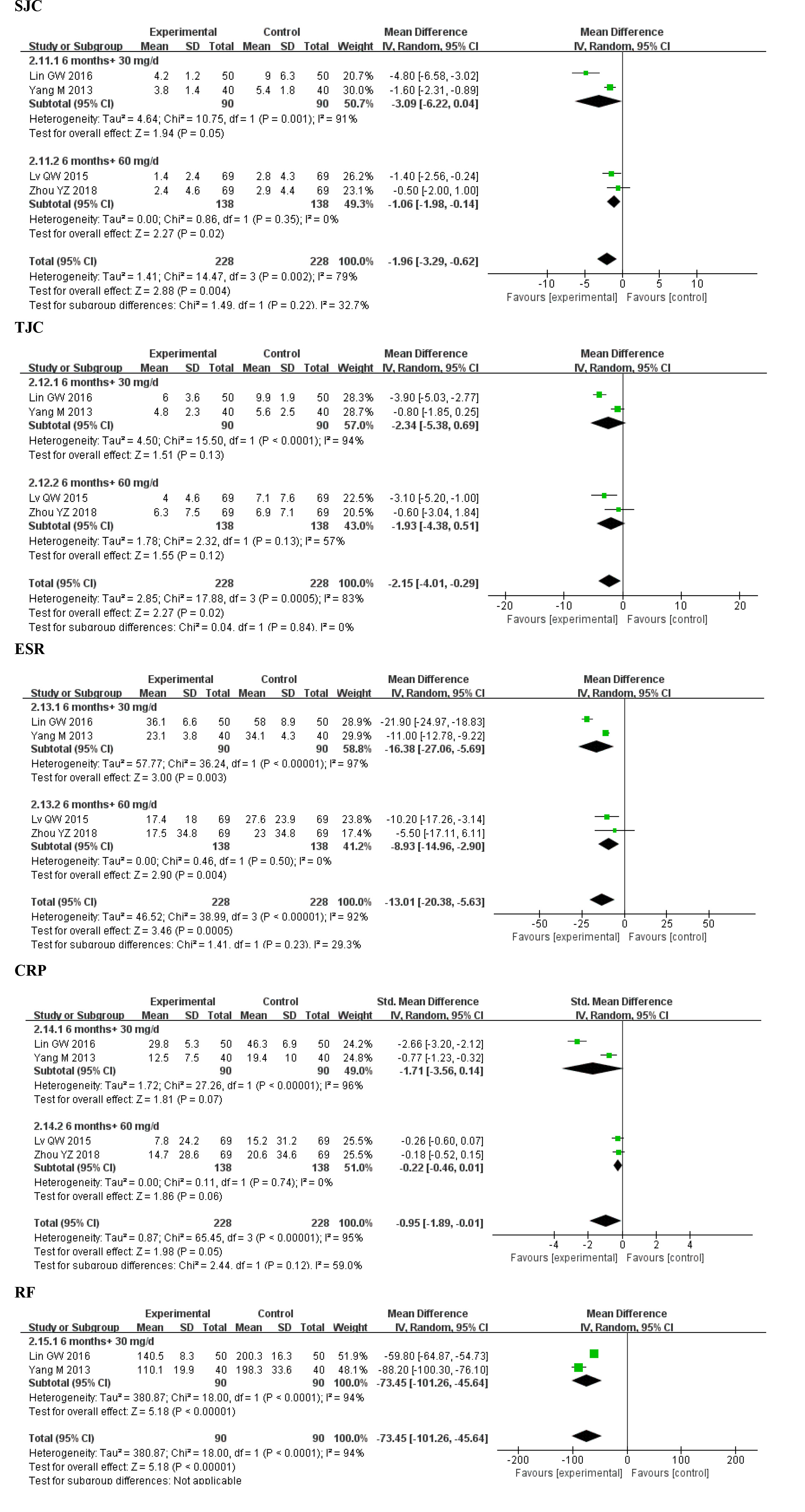

Supplement: Supplementary Materials — Supplementary 1. Supplementary Information 1: items regarding the PRISMA checklist for network meta-analysis. Supplementary Information 2: detailed search strategies. Supplementary Information 3: a list of all excluded papers. Supplementary 2Supplementary Information 4: quality assessment using the GRADE approach. Supplementary Figure 1: forest plots for the secondary outcomes of TG adjuvant MTX therapy. Supplementary Figure 2: forest plots for the secondary outcomes of a three-month course of TG adjuvant MTX therapy at a dose of 30 mg/day. Supplementary Figure 3: forest plots for the secondary outcomes of the different courses and doses of TG adjuvant MTX therapy. Supplementary Figure 4: forest plots for the safety of TG adjuvant MTX therapy. Supplementary Figure 5: forest plots for the safety of a three-month course of TG adjuvant MTX therapy at the dose of 30 mg/day. Supplementary Figure 6: forest plots for the safety of the different courses and doses of TG adjuvant MTX therapy. [file 1251478.f1.zip › 1251478.f1/Supplement Fig 3 (1).jpg]

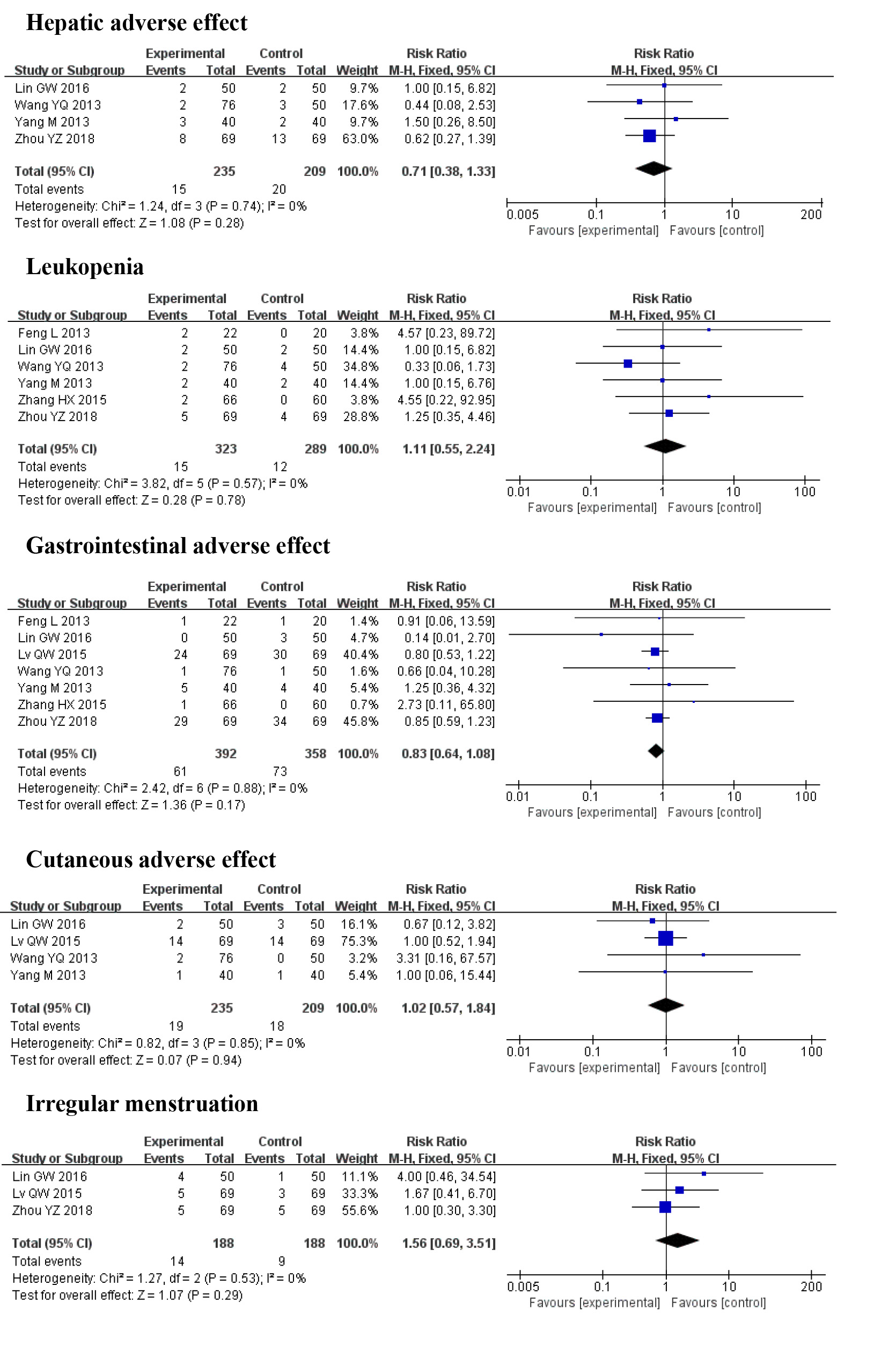

Supplement: Supplementary Materials — Supplementary 1. Supplementary Information 1: items regarding the PRISMA checklist for network meta-analysis. Supplementary Information 2: detailed search strategies. Supplementary Information 3: a list of all excluded papers. Supplementary 2Supplementary Information 4: quality assessment using the GRADE approach. Supplementary Figure 1: forest plots for the secondary outcomes of TG adjuvant MTX therapy. Supplementary Figure 2: forest plots for the secondary outcomes of a three-month course of TG adjuvant MTX therapy at a dose of 30 mg/day. Supplementary Figure 3: forest plots for the secondary outcomes of the different courses and doses of TG adjuvant MTX therapy. Supplementary Figure 4: forest plots for the safety of TG adjuvant MTX therapy. Supplementary Figure 5: forest plots for the safety of a three-month course of TG adjuvant MTX therapy at the dose of 30 mg/day. Supplementary Figure 6: forest plots for the safety of the different courses and doses of TG adjuvant MTX therapy. [file 1251478.f1.zip › 1251478.f1/Supplement Fig 4 (1).jpg]

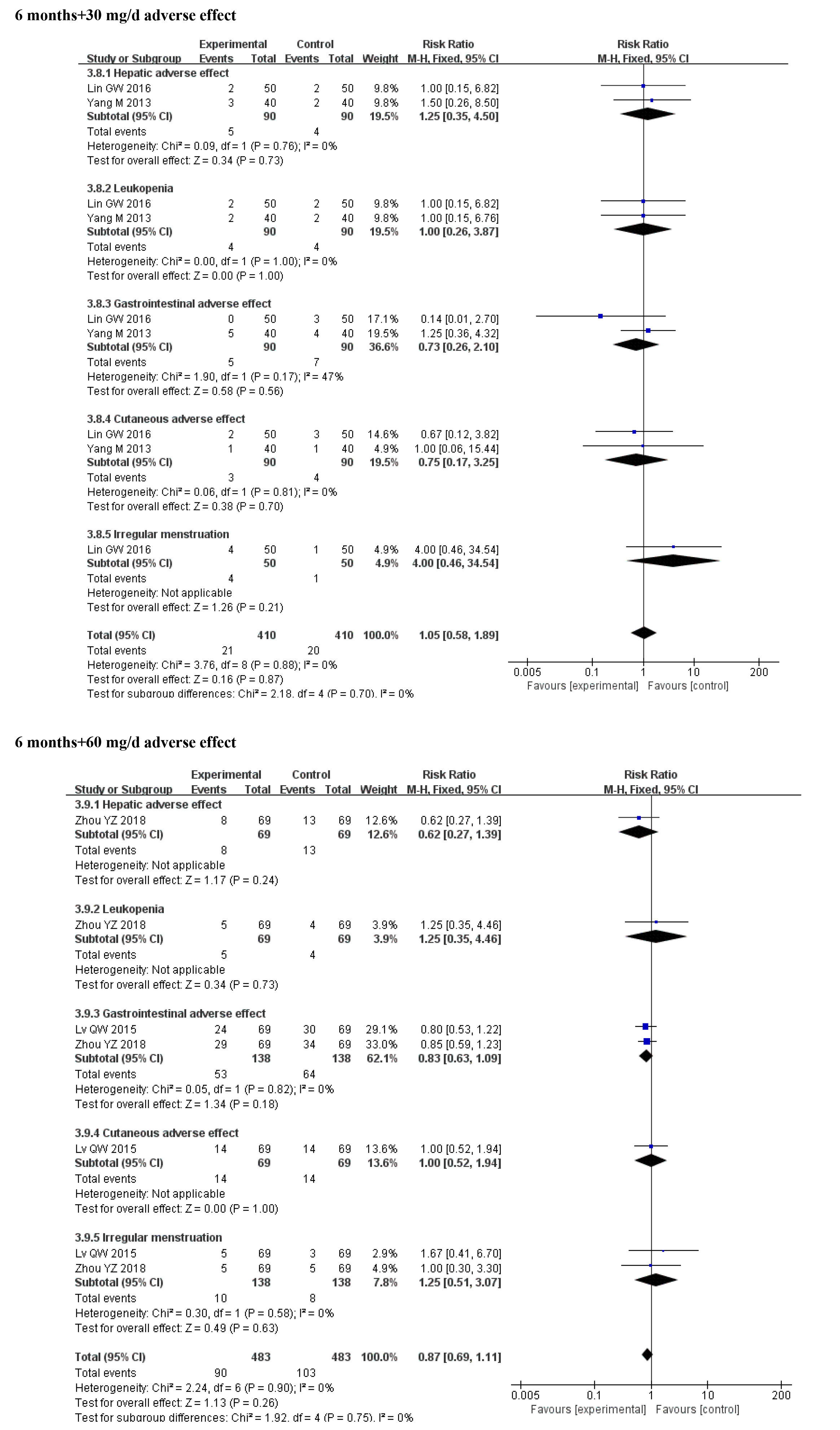

Supplement: Supplementary Materials — Supplementary 1. Supplementary Information 1: items regarding the PRISMA checklist for network meta-analysis. Supplementary Information 2: detailed search strategies. Supplementary Information 3: a list of all excluded papers. Supplementary 2Supplementary Information 4: quality assessment using the GRADE approach. Supplementary Figure 1: forest plots for the secondary outcomes of TG adjuvant MTX therapy. Supplementary Figure 2: forest plots for the secondary outcomes of a three-month course of TG adjuvant MTX therapy at a dose of 30 mg/day. Supplementary Figure 3: forest plots for the secondary outcomes of the different courses and doses of TG adjuvant MTX therapy. Supplementary Figure 4: forest plots for the safety of TG adjuvant MTX therapy. Supplementary Figure 5: forest plots for the safety of a three-month course of TG adjuvant MTX therapy at the dose of 30 mg/day. Supplementary Figure 6: forest plots for the safety of the different courses and doses of TG adjuvant MTX therapy. [file 1251478.f1.zip › 1251478.f1/Supplement Fig 6 (1).jpg]

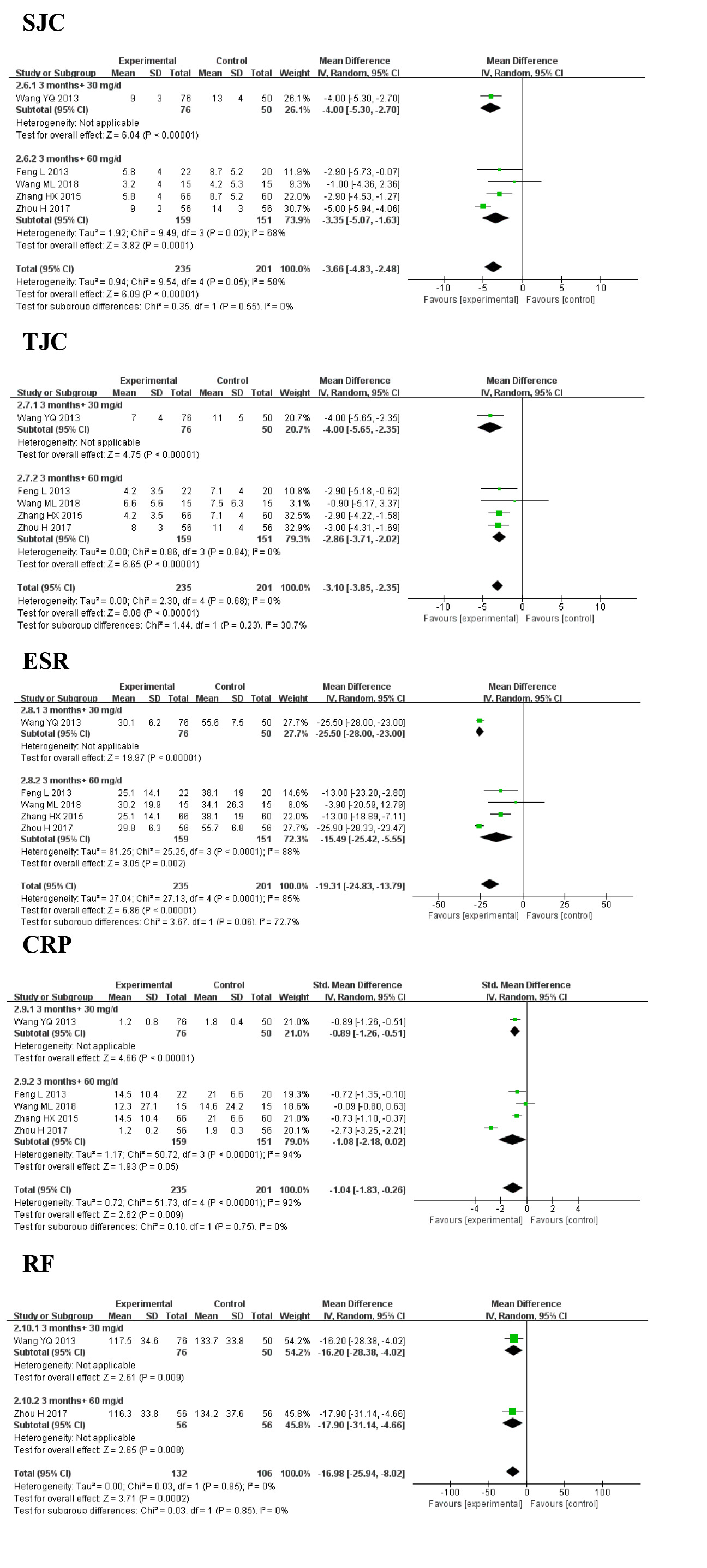

Supplement: Supplementary Materials — Supplementary 1. Supplementary Information 1: items regarding the PRISMA checklist for network meta-analysis. Supplementary Information 2: detailed search strategies. Supplementary Information 3: a list of all excluded papers. Supplementary 2Supplementary Information 4: quality assessment using the GRADE approach. Supplementary Figure 1: forest plots for the secondary outcomes of TG adjuvant MTX therapy. Supplementary Figure 2: forest plots for the secondary outcomes of a three-month course of TG adjuvant MTX therapy at a dose of 30 mg/day. Supplementary Figure 3: forest plots for the secondary outcomes of the different courses and doses of TG adjuvant MTX therapy. Supplementary Figure 4: forest plots for the safety of TG adjuvant MTX therapy. Supplementary Figure 5: forest plots for the safety of a three-month course of TG adjuvant MTX therapy at the dose of 30 mg/day. Supplementary Figure 6: forest plots for the safety of the different courses and doses of TG adjuvant MTX therapy. [file 1251478.f1.zip › 1251478.f1/Supplementary Fig 2 (2).jpg]

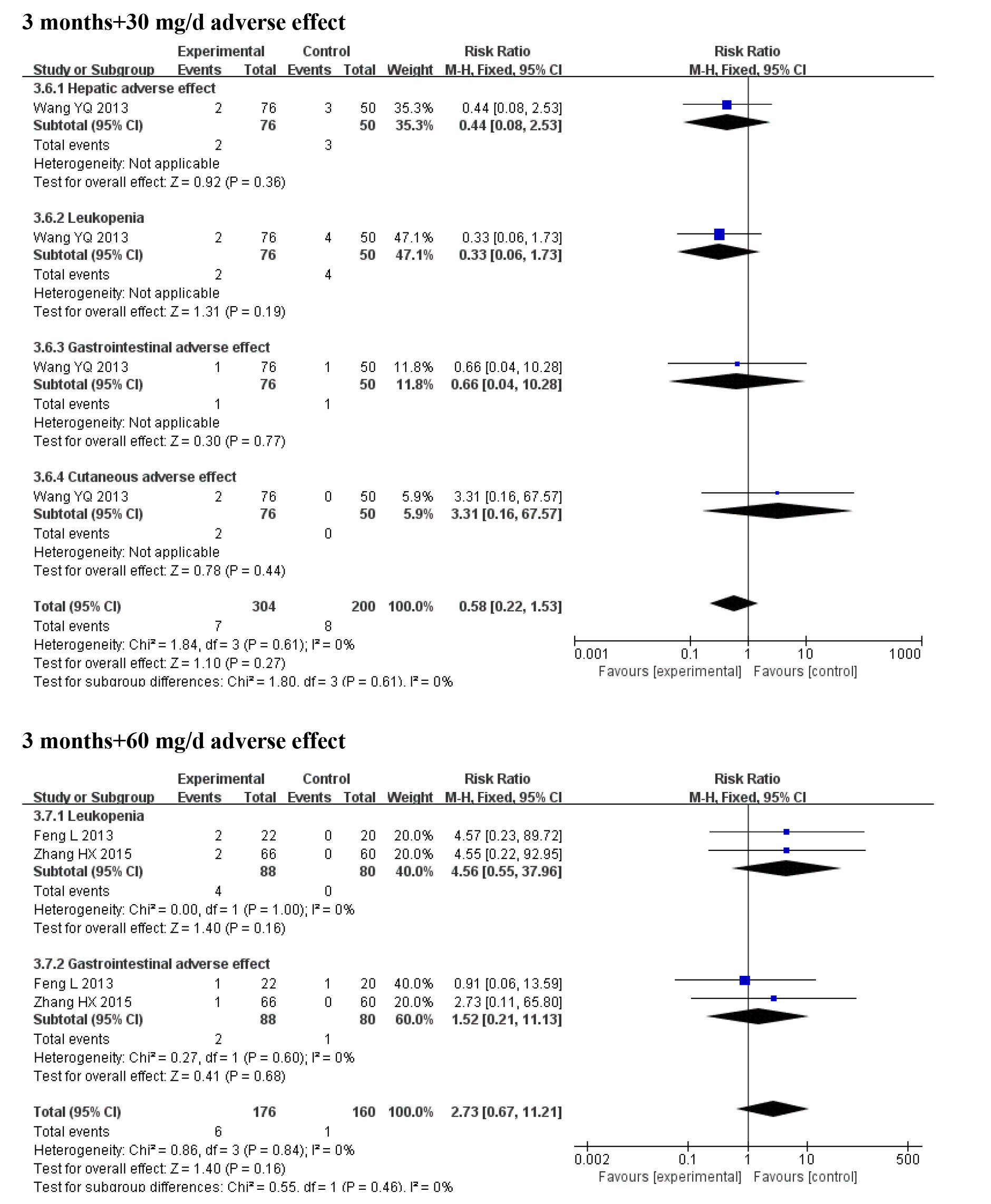

Supplement: Supplementary Materials — Supplementary 1. Supplementary Information 1: items regarding the PRISMA checklist for network meta-analysis. Supplementary Information 2: detailed search strategies. Supplementary Information 3: a list of all excluded papers. Supplementary 2Supplementary Information 4: quality assessment using the GRADE approach. Supplementary Figure 1: forest plots for the secondary outcomes of TG adjuvant MTX therapy. Supplementary Figure 2: forest plots for the secondary outcomes of a three-month course of TG adjuvant MTX therapy at a dose of 30 mg/day. Supplementary Figure 3: forest plots for the secondary outcomes of the different courses and doses of TG adjuvant MTX therapy. Supplementary Figure 4: forest plots for the safety of TG adjuvant MTX therapy. Supplementary Figure 5: forest plots for the safety of a three-month course of TG adjuvant MTX therapy at the dose of 30 mg/day. Supplementary Figure 6: forest plots for the safety of the different courses and doses of TG adjuvant MTX therapy. [file 1251478.f1.zip › 1251478.f1/Supplementary Fig 5 (1).jpg]
